# Nocturnal features and daytime characteristics in narcolepsy: reliability and diagnostic relevance for NT1 Versus NT2

**DOI:** 10.1093/sleep/zsag180

**Published:** 2026-07-07

**Authors:** Emily Schlafly, Marta Karas, Yishu Gong, Lucie Barateau, Brian Tracey, Dmitri Volfson, Yves Dauvilliers

**Affiliations:** Takeda Development Center Americas, Inc., Cambridge, MA, United States; Takeda Development Center Americas, Inc., Cambridge, MA, United States; Takeda Development Center Americas, Inc., Cambridge, MA, United States; Sleep-Wake Disorders Center, Department of Neurology, Gui de Chauliac Hospital, CHU Montpellier, France; National Reference Network for Narcolepsy, Montpellier, France; Institute for Neurosciences of Montpellier (INM), INSERM, University of Montpellier, Montpellier, France; Takeda Development Center Americas, Inc., Cambridge, MA, United States; Takeda Development Center Americas, Inc., Cambridge, MA, United States; Sleep-Wake Disorders Center, Department of Neurology, Gui de Chauliac Hospital, CHU Montpellier, France; National Reference Network for Narcolepsy, Montpellier, France; Institute for Neurosciences of Montpellier (INM), INSERM, University of Montpellier, Montpellier, France

**Keywords:** narcolepsy, narcolepsy–clinical assessment, EEG analysis, REM sleep, statistics, nocturnal polysomnography, maintenance of wakefulness test, test–retest reliability, hypnodensity

## Abstract

**Study Objectives:**

Assess test–retest reliability of nighttime sleep and daytime maintenance of wakefulness tests (MWTs) over a short time interval in drug-free individuals with narcolepsy; evaluate the diagnostic utility of features with good reliability.

**Methods:**

We analyzed the placebo arms from clinical trials in narcolepsy type 1 (NT1; n = 17; NCT05687903) and narcolepsy type 2 (NT2; n = 19; NCT05687916). Participants visited the clinic three times, four weeks apart, which included nocturnal polysomnography (nPSG) and MWT the following day. From the nPSG and daytime MWT, we derived 440 features and assessed test–retest reliability, visit effects, and NT1-NT2 differences.

**Results:**

Quantitative electroencephalography (qEEG) showed high reliability, with the top 10 features showing reliability >0.88; the 10 most reliable hypnogram features ranged from 0.68–0.78. qEEG and MWT sleep onset latencies (SOLs) were less reliable in NT2. N1 percentage, stage shift index, and wake after sleep onset were reliable and differed between diagnoses (r = 0.54–0.75; higher in NT1, *p* < .05). MWT SOLs at 2 p.m. and 4 p.m. demonstrated good reliability in NT1 (r = 0.89, 0.87) and differed between diagnoses (shorter in NT1; *p* = .037, .010). Hypnodensity Wake–REM mixtures were reliable (r = 0.83) and differed between diagnoses (higher in NT1, *p* = .001).

**Conclusions:**

qEEG features showed the strongest reliability but rarely distinguished diagnoses. Measures of sleep fragmentation and wakefulness reliably differentiated NT1 from NT2 and are practical for clinical assessments and trials. Hypnodensity features, though not yet used clinically, are reliable and may offer diagnostic value. NT2 exhibited greater variability in nighttime sleep and daytime wakefulness across visits.

## Graphical abstract

**Graphical abstract:**
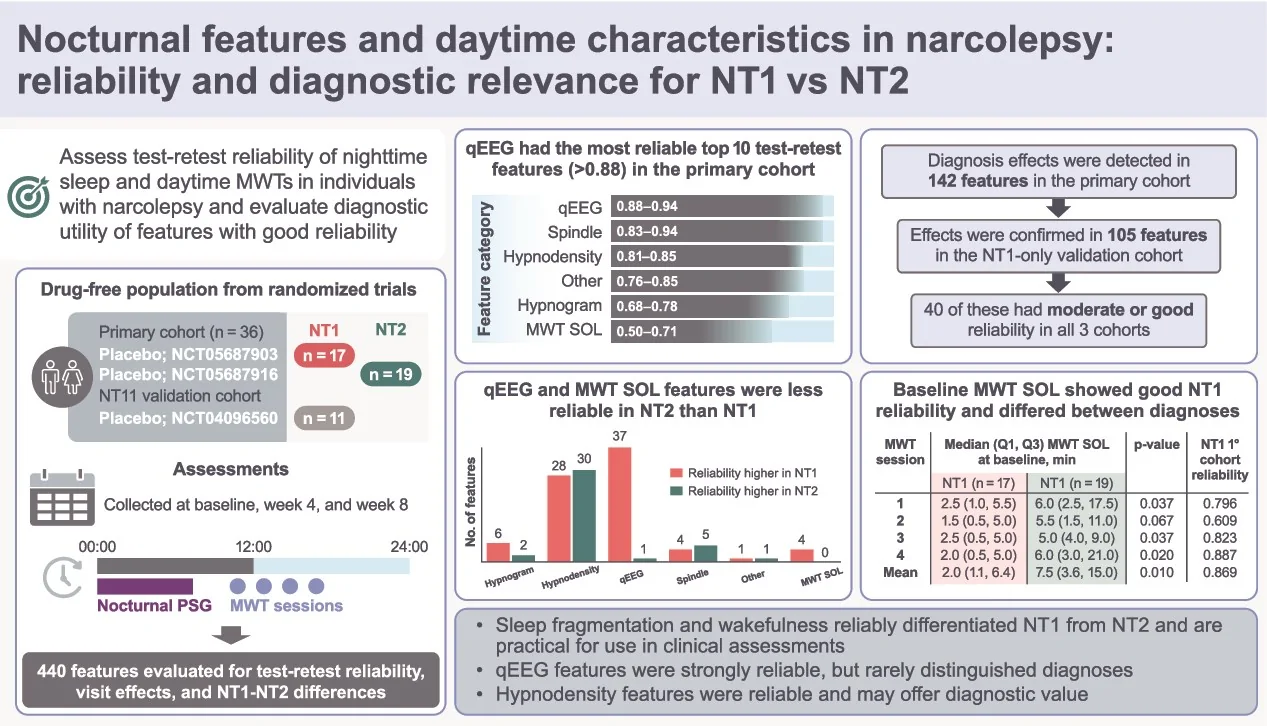

**Statement of Significance**

This study addresses an important gap by characterizing visit-to-visit reliability of sleep and wakefulness endpoints in narcolepsy type 1 (NT1) and type 2 (NT2). Specifically, reliability is assessed by comparing nocturnal polysomnography (nPSG) and maintenance of wakefulness test (MWT) results of individuals from the placebo arms of randomized control trials. A wide variety of features are assessed, including those derived from manual sleep staging, quantitative electroencephalography and spindles analysis, and hypnodensities generated from automated sleep staging. Feature reliability was validated in an independent clinical trial. Improved reliability estimates should aid in clinical interpretation and differential diagnosis, as well as informing the design of future interventional studies.

## Introduction

Narcolepsy is classified into two disorders: narcolepsy type 1 (NT1), or narcolepsy with cataplexy, and narcolepsy type 2 (NT2), known as narcolepsy without cataplexy [1]. Both NT1 and NT2 are characterized by excessive daytime sleepiness; what differentiates them is the presence of cataplexy, a sudden, emotionally triggered loss of muscle tone, which is pathognomonic for NT1 [1]. NT1 occurs due to the loss of hypothalamic orexin (hypocretin) neurons resulting in low or undetectable cerebrospinal fluid (CSF) orexin levels. In contrast, the etiology of NT2 remains unclear; while the majority of individuals with NT2 have normal orexin CSF levels, a small subgroup has intermediate levels [2–4].

Recently, improved characterization of nocturnal polysomnography (nPSG) has allowed better differentiation of individuals with NT1 and NT2 from other individuals with hypersomnolence and controls. This differentiation is enabled by multiple sleep metrics, including sleep architecture, sleep stage transition probabilities, hypnodensities, sleep spindles, and quantitative electroencephalogram (qEEG) features [5–10]. However, to date, limited research has addressed whether nocturnal sleep characteristics that differentiate people with NT1 and NT2 remain consistent over time within individuals. This question is especially relevant in clinical trials, where understanding the reliability of these features helps determine whether post-treatment changes reflect medication effects or inherent within-participant variability. Additionally, few studies have evaluated test–retest reliability of daytime sleepiness and wakefulness [11–19], especially in central disorders of hypersomnolence [14, 18, 20]. To the best of our knowledge, these studies assessed daytime sleepiness using the multiple sleep latency test (MSLT) with inconsistent results; none have evaluated test–retest reliability in maintenance of wakefulness tests (MWTs).

In this study, we aimed (1) to assess the test–retest reliability of sleep parameters (nocturnal features and daytime characteristics) by analyzing repeated PSG and MWTs from individuals with NT1 and NT2 enrolled in the placebo arms of two randomized controlled trials, ensuring stable and standardized conditions, (2) to compare test–retest reliability in NT1 versus NT2 for the same parameters, and (3) to assess the relevance of individual sleep features in differentiating between NT1 and NT2 populations.

## Materials and methods

### Study design and participants

The study population and nPSG data analyzed in this study were previously reported and included individuals with NT1 and NT2 [21–23]. We analyzed two cohorts of clinical trial participants with narcolepsy—a primary cohort consisting of participants with NT1 and NT2 randomized in NCT05687903 and NCT05687916, and a validation cohort of participants with NT1 randomized in NCT04096560 [21, 22]. In the primary cohort, participants underwent nPSG with MWTs (4 sessions, at 10 a.m., 12 noon, 2 p.m., and 4 p.m.) at baseline, Week 4, and Week 8; in the validation cohort, study protocols were the same, except that participants underwent nPSG only at baseline and Week 8. In both cohorts, participants were aged 18–70 years with a clear diagnosis previously confirmed by nPSG and MSLT, performed within the last 10 years. Among inclusion criteria, all participants had an Epworth Sleepiness Scale score ≥10 at the baseline visit, had four or more partial or complete episodes of cataplexy per week during screening (for NT1), and agreed to discontinue all medications for narcolepsy, if applicable. Washout duration requirement was at least 5 half-lives of any medication. Participants were not allowed to freely add, remove, or change medications during the treatment period. Further inclusion and exclusion criteria have been described previously [21, 22]. In the primary cohort, participants took part in the study between January and December 2023. In the validation cohort, participants took part in the study between May 2020 and November 2021. All participants resided in North America, Europe, Japan, and Australia (primary cohort only). The present work includes only data from participants in the placebo arms of these trials.

The trials were approved by regulatory authorities in each country and the institutional review board or ethics committee at each site and were performed in accordance with the trial protocols, the Declaration of Helsinki, and the International Council for Harmonisation E6 Good Clinical Practice guidelines. Written informed consent was obtained from all participants. This work follows the Strengthening the Reporting of Observational Studies in Epidemiology (STROBE) guidelines.

### Data collection and preprocessing

Sleep was manually scored at a central facility (Clinilabs) following American Academy of Sleep Medicine (AASM) standards. To promote consistency across study visits, recordings from each participant were scored by a single scorer (i.e. a one-to-one scorer-to-participant assignment). Hypnogram-derived nPSG features and daytime MWT sleep onset latencies (SOLs) were derived from manual scoring, with the remaining features being computed from raw sensor data. qEEG features were computed using custom Python code described previously [10]. Spindles and eye movement features were computed using Yet Another Sleep Algorithm (YASA) (YASA version 0.6.5, parameters in Supplementary Methods) [24]. Hypnodensity features were computed using an updated version of the neural network previously described by Stephansen et al. 2018 [9], and available at https://github.com/Stanford-STAGES/sleep-staging/tree/r1.0.0 [25].

### Features analyzed

We characterized 440 features derived from nPSG and daytime MWT and described in prior publications [5, 8–10]. We grouped features into six groups (hypnogram, hypnodensity, qEEG, spindles, MWT, and other, i.e. eye and leg movements) to understand the characteristics of each aspect of sleep.

*Visually scored hypnogram (37 features; nPSG).* We analyzed seven whole-night features, including the sleep onset latency, rapid eye movement (REM) latency (the latency between sleep onset and the first epoch of REM stage sleep), total sleep time (TST), the number of arousals, the stage shift index (the number of times the hypnogram changes from one sleep stage to another, including wake, divided by total time in bed), the proportion of time in non-REM (NREM) sleep (count of NREM epochs divided by the number of scored epochs), and the number of direct sudden onset REM periods (dREMs) defined as at least five epochs of wake or N1 followed by at least two epochs of REM [6, 8]. We counted the number of dREMs throughout the entire extent of the PSG as described previously by Karas et al. 2025 [8], including any that occurred during the first 15 minutes. We also analyzed the proportion of time in each individual sleep stage (five features), the mean bout duration of each individual sleep stage (five features), and transition rates between different stages (20 features = 5 source stages × 5 destination stages, omitting five transitions that occurred very rarely [N1-to-N3, N3-to-REM, N3-to-N1, REM-to-N3, Wake-to-N3]).

*Hypnodensity (248 features; nPSG).* We analyzed eight measures for each of 31 sleep stages and sleep stage mixtures (geometric mean of each combination of sleep stages). These measures were defined in detail by Stephansen et al. 2018 [9]. Briefly, the hypnodensities and hypnodensity mixtures were: four accumulation times (time points at which 5%, 10%, 30%, 50% of the total value has accumulated); prevalence (analogous to proportion of time in each stage), prevalence weighted by time to 50% accumulation, the maximum achieved value, and Shannon entropy.

*qEEG (120 features; nPSG).* We computed four measures across six frequency bands and all five sleep stages [8, 10]. Each measure was computed for each frequency band in each 30-second epoch; then we computed the mean for each sleep stage. The four measures were the mean power, the baseline power (the 25th percentile of the power), the peak-to-baseline power ratio, and the frequency at which the peak power was detected. Frequency bands were delta (0.5–4 Hz), theta (4–8 Hz), alpha (8–12 Hz), sigma (12–16 Hz), beta (16–30 Hz), and gamma (30–47 Hz); sleep stages were Wake, N1, N2, N3, and REM as determined by the visually scored hypnogram.

*Sleep spindles (26 features; nPSG).* Spindles were detected using YASA version 0.6.5 [24] in two frequency bands (high frequency spindles with frequencies in 12–15 Hz and low frequency spindles with frequencies in 9–12 Hz) and two stages (N2 and N3 based on visually scored hypnogram). We analyzed five measures: mean absolute power, mean relative power, density (count per minutes in stage), mean duration, and mean frequency. In N3, we additionally analyzed the percent of spindles coupled to slow-waves, the mean amplitude of the corresponding slow-wave (where detected) at the spindle peak, and mean amplitude of the z-scored slow-wave signal at the spindle peak (Supplementary Methods).

*Eye movements during REM sleep (two features; nPSG).* Eye movements were detected using YASA version 0.6.5 [24] with minor modifications. Briefly, electrooculography channels were filtered to between 0.5 and 5 Hz, then centered relative to the median and scaled by the scaled median absolute deviation (sMAD), 1.4826 times the median absolute deviation [26]. Putative eye movements were detected by identifying peaks in the negative product of the channels; a detection was declared if the absolute amplitude on both channels was between 2 and 20 sMAD. Outliers were detected based on the interquartile range of the amplitudes and rise/fall rates; events with amplitudes or rise/fall rates more than 1.5 times below the first quartile or above the third quartile were excluded. The two measures we considered here were eye movement density (the number of eye movements per minute in REM sleep) and the average duration of individual eye movements in manually scored stage REM sleep.

*Periodic limb movements during sleep (PLMS) (two features; nPSG).* PLMS were annotated at the time of scoring according to AASM standards. We included two measures characterizing PLMS: the total number of PLMS bouts (where a bout is defined per the AASM as a series of individual limb movements, occurring in series of at least four consecutive movements, with 5–90 seconds between consecutive movements), and the number of PLMS per hour.

*MWT SOLs (five features; daytime).* SOL (in minutes) at each of the four sessions (10 a.m., 12 noon, 2 p.m., and 4 p.m.) and the mean across the four sessions.

#### Feature transformation

To transform features, we performed a two-pass procedure: we performed a preliminary transformation to identify and remove outliers, then repeated the procedure once outliers were removed.

Within each feature, observations, denoted *y* below, were centered and scaled relative to the median (C) and interquartile range (${S}_1$), respectively, then transformed using a modulus transform to reduce the skew, and finally scaled by the standard deviation (${S}_2$) so that variance and standard deviation were equal to 1:


$${y}^{\ast }=\left(y-C\right)/{S}_1 \quad \text{Robust standardization}$$

$${z}^{\ast }=\mathit{\operatorname{sign}}\left({y}^{\ast}\right)\mathit{\ln}\left(\left|{y}^{\ast}\right|+1\right) \quad{\text{Modulus transformation}}$$

$$z={z}^{\ast }/{S}_2 \quad{\mathrm{Standardization}}$$

To avoid fitting the model to observations that exert disproportionate influence, we excluded transformed data points identified as prediction outliers based on studentized leave-one-out cross-validation residuals (*rstudent* function from the *stats* package in R) [27, 28]. If any participant was left with only a single observation, this was also excluded. Within each feature, at most 11 observations were excluded as outliers (≈11%) and in more than 75% of features, four or fewer observations were excluded. After excluding outliers, we recomputed ${y}^{\ast }$, ${z}^{\ast }$, and *z* using updated values of *C*, ${S}_1$, and ${S}_2$.

For the validation cohort, we followed the same procedure where we removed outliers in a first pass, then repeated the transformation after outliers were excluded. However, in the second pass, we used the centering and scaling parameters ($C,{S}_1,{S}_2$) from the primary cohort.

### Test–retest reliability

For each feature, we had observations from multiple participants at three different visits. We computed reliability ${r}_{xx}$ as the proportion of total variance not attributable to error:


$${r}_{xx}=1-\frac{\sigma_{\mathrm{\varepsilon}}^2}{V_X}$$

where ${\mathrm{\sigma}}_{\mathrm{\varepsilon}}^2$ is residual error variance and ${\mathrm{V}}_{\mathrm{X}}$ is total variance [29]. We estimated ${\sigma}_{\epsilon}^2$ for each feature by fitting linear mixed effects models (LMMs) with participant-level random intercepts to the transformed observations. Since the transformed features were standardized to have total variance equal to one (${V}_X=1$), estimated reliability is given by 1 - ${\hat{\mathrm{\sigma}}}_{\mathrm{\varepsilon}}^2$.

When all of the variance is attributable to residual error and participant-specific differences, ${r}_{xx}$ is equivalent to the intraclass correlation coefficient (ICC), another measure commonly used in test–retest reliability analyses [30–33]. We therefore adopted thresholds used to interpret ICC: values between 0.5 and 0.75 were interpreted as moderate reliability and values greater than 0.75 were interpreted as good reliability [34, 35]. For the present work, we chose to use ${r}_{xx}$ rather than ICC because ICC expresses the proportion of total variance attributable specifically to between-participant differences, and its value is strongly influenced by the amount of heterogeneity in the study population. As a result, ICC can be low even when measurement error is minimal if individuals do not differ substantially on the feature studied. In contrast, ${r}_{xx}$ isolates the contribution of the residual error and does not conflate measurement reliability with population variability. Given that our objective was to evaluate within-participant reliability across repeated measurements rather than to quantify between-participant discriminability, ${r}_{xx}$ provided a more appropriate reliability metric for this analysis.

### Statistical analysis

To estimate reliability with confidence intervals, we fit random effects models with participant-level random intercepts to the transformed observations. We first estimated reliability in the combined population by fitting models to data from all participants in the primary cohort; we then estimated reliability in each diagnosis subgroup and in the validation cohort by fitting separate models to participants from each group. To compare reliability between NT1 and NT2, we used Levene’s test for homogeneity of variances. To examine diagnosis effects, we fit an LMM with fixed effects for diagnosis (NT2 versus NT1) and age, and random intercepts for participants.

To examine study visit (i.e. baseline, Week 4, and Week 8) effects, we fit an LMM with fixed effects for study visit where Week 4 and Week 8 were grouped into a single “on-placebo” category. For the sleep stage proportions, we also assessed changes from baseline in each quarter of the night. To do so, we computed the proportion of time in each sleep stage in each quarter of the night and refit the LMMs substituting the quarter-night observations as the dependent variable.

In our tables, we report original *p*-values as well *p*-values adjusted using the Benjamini-Hochberg false discovery rate (FDR) correction [36], similar to Karas et al., 2025 [8]. The FDR correction is applied within each task (comparison of reliability, test for night effect, test for diagnosis effect) and feature category listed above. Given the exploratory nature of this study, our results focus on findings based on uncorrected *p*-values. All analyses are evaluated against a significance threshold of *α =.05*. Statistical tests were performed in R (version 4.3.3).

## Results

### Study population

Overall, 47 participants from the placebo arms were analyzed for this study (28 NT1, 19 NT2). In the primary cohort, we analyzed 17 participants with NT1 (aged 19–57 years, 11 women; NCT05687903) and 19 participants with NT2 (aged 24–69 years, 16 women; NCT05687916; Table 1). All participants had MWT sessions at all three visits. Some nPSG recordings were excluded due to file read errors, resulting in three nights of nPSG from 14/17 participants with NT1 and 17/19 participants with NT2, respectively. To replicate our findings, we additionally analyzed a validation cohort comprising 11 participants with NT1 from a separate clinical trial (aged 21–47 years, five women; NCT04096560; Table 1), with PSG and MWT sessions available at baseline and Week 8. No medication additions or changes were documented in the analyzed participants during the study period, consistent with the protocol inclusion criteria. No participants reported illness coinciding with nPSG recordings. Minor adverse events were reported in a small number of participants but were consistent with those commonly encountered in routine clinical settings.

**Table 1:** Participant demographics and sleep architecture characteristics at baseline

	NT1 (n = 17)	NT2 (n = 19)	NT1 validation (n = 11)
**Demographics**			
Female, n (%)	11 (64.7)	16 (84.2)	5 (45.5)
Sites, n	12	12	9
Mean age (range), years	36.4 (19, 57)	39.3 (24, 69)	35.0 (21, 47)
White, n (%)	14 (82.4)	11 (57.9)	6 (54.5)
**Sleep architecture characteristics, median (IQR)**			
TST, min	437 (407, 463)	420 (389, 442)	397 (374, 408)
Sleep efficiency, %	84.8 (75.2, 92.6)	88.2 (81.0, 91.6)	83.1 (78.0, 84.7)
N1, %	6.66 (4.93, 10.3)	6.43 (5.73, 8.70)	9.98 (8.12, 14.7)
N2, %	46.1 (39.4, 57.4)	52.2 (48.1, 53.9)	35.0 (33.1, 42.4)
N3, %	12.1 (8.81, 15.1)	7.29 (2.60, 10.0)	14.6 (12.0, 17.0)
REM, %	14.6 (12.7, 18.0)	17.4 (15.9, 19.2)	18.5 (18.0, 20.5)
Sleep latency, min	4.50 (1.50, 8.12)	10.5 (3.00, 20.5)	2.50 (0.75, 4.50)
REM latency, min	76.0 (16.9, 108.0)	78.5 (57.0, 110.0)	46.5 (10.5, 55.0)
WASO, min	51.0 (31.5, 93.9)	45.5 (32.5, 61.0)	78.0 (70.5, 104.0)
Nocturnal SOREMPs, n (%)	4 (28.6)	1 (5.9)	3 (27.3)

IQR, interquartile range; NT1, narcolepsy type 1; NT2, narcolepsy type 2; REM, rapid eye movement; SOREMP, sleep onset rapid eye movement period; TST, total sleep time; WASO, wake after sleep onset.

### Reliability of sleep features in the combined population

We characterized test–retest reliability in the 440 features described above. Across all features analyzed, 106 had good reliability (${r}_{xx}$ > 0.75). The most reliable features from each category are listed in Table 2; reliability estimates for all features are provided in Supplementary Table 1. qEEG and spindle features showed the highest reliability, while features describing sleep architecture (hypnogram and hypnodensity features) tended to be lower (Table 2).

**Table 2:** Top 10 most reliable test–retest features within each feature category (combined primary cohort; n = 36)

	Hypnogram		Hypnodensity		qEEG		Spindle		Other		MWT	
Rank	Feature	R (95%CI)	Feature	R (95%CI)	Feature	R (95%CI)	Feature	R (95%CI)	Feature	R (95%CI)	Feature	R (95%CI)
1	N3 → N2	0.78 (0.68, 0.84)	Wake-N1-REM Prev	0.85 (0.79, 0.90)	N2 theta, baseline	0.94 (0.91, 0.96)	N2 LowF, Freq	0.94 (0.91, 0.95)	PLMI	0.85 (0.78, 0.89)	Mean SOL	0.71 (0.58, 0.79)
2	N3 → N3	0.77 (0.67, 0.84)	Wake-N1-N2-REM AT50(w)	0.85 (0.78, 0.89)	REM alpha, peak amp	0.94 (0.91, 0.95)	N2 HighF, RMS	0.93 (0.91, 0.95)	PLM bouts	0.84 (0.77, 0.89)	4 p.m. SOL	0.56 (0.37, 0.68)
3	N1 → N2	0.74 (0.63, 0.81)	Wake-N1-REM AT50(w)	0.84 (0.77, 0.89)	N2 gamma, peak freq	0.93 (0.89, 0.95)	N2 LowF, RMS	0.93 (0.90, 0.95)	REM EM Dens	0.82 (0.74, 0.87)	2 p.m. SOL	0.55 (0.36, 0.68)
4	SSI	0.73 (0.62, 0.81)	Wake-N1-N3-REM Prev	0.84 (0.77, 0.88)	N2 alpha, peak amp	0.93 (0.89, 0.95)	N3 LowF, Freq	0.91 (0.87, 0.94)	REM EM Duration	0.76 (0.65, 0.83)	12 noon SOL	0.54 (0.35, 0.67)
5	N3 %	0.72 (0.60, 0.80)	Wake-REM AT50(w)	0.84 (0.76, 0.88)	N3 theta, baseline	0.93 (0.89, 0.95)	N3, LowF, RMS	0.91 (0.86, 0.93)			10 a.m. SOL	0.50 (0.29, 0.63)
6	N2 bout duration	0.72 (0.59, 0.80)	Wake-REM Prev	0.83 (0.76, 0.88)	REM sigma, peak amp	0.92 (0.88, 0.94)	N2 HighF, Dens	0.87 (0.81, 0.91)				
7	N1 → N1	0.70 (0.57, 0.78)	Wake-N2-REM Prev	0.81 (0.73, 0.87)	Wake sigma, peak freq	0.91 (0.87, 0.94)	N3 HighF, RMS	0.87 (0.80, 0.91)				
8	N2 → N2	0.69 (0.55, 0.78)	Wake-N2-REM AT50(w)	0.81 (0.73, 0.86)	N3 alpha, peak freq	0.91 (0.87, 0.94)	N3 HighF, Dens	0.86 (0.78, 0.90)				
9	N1 %	0.68 (0.54, 0.77)	Wake-N1-N3-REM AT50(w)	0.81 (0.72, 0.87)	REM alpha, baseline	0.89 (0.85, 0.92)	N2 LowF, RelPower	0.85 (0.79, 0.89)				
10	N2 %	0.68 (0.54, 0.77)	Wake-N1 AT50(w)	0.81 (0.72, 0.86)	REM beta, peak amp	0.89 (0.83, 0.92)	N3 HighF, Freq	0.83 (0.75, 0.88)				

Amp, amplitude; AT50(w), timing-weighted prevalence; CI, confidence interval; Dens, density; EM, eye movement; Freq, frequency; PLM, periodic limb movement; PLMI, periodic limb movement index; Prev, prevalence; REM, rapid eye movement; RMS, root mean square amplitude; SOL, sleep onset latency; SSI, stage shift index.

#### Reliability in qEEG features

Out of 120 qEEG features, 92% had at least moderate reliability (${r}_{xx}$ between 0.5 and 0.75, N = 110) and 52% had good reliability (${r}_{xx}$ > 0.75, N = 62). The peak-to-baseline amplitude ratio, baseline, and frequency measures showed good reliability in a majority of frequency bands and sleep stages; the mean power measure more often had only moderate reliability. Measurements during REM sleep were particularly reliable, with good reliability in all bands apart from delta, which typically had only poor to moderate reliability (Supplementary Figure 1). To compare frequency bands, we ranked the reliability in each frequency band and found that alpha tends to be the most reliable, followed by theta and sigma.

#### Reliability in sleep spindles

We examined reliability in sleep spindles in N2 and N3 sleep in two frequency bands (12–15 Hz, 9–12 Hz) and found good reliability in 15/26 features (58%). Absolute power and density demonstrated good reliability across both frequency bands and in both stages (Supplementary Figure 2). Spindle duration had good reliability for 12–15 Hz spindles during N2 sleep, but duration of low-frequency spindles and spindles during N3 had poor reliability. Features of N2 spindles were more reliable than features of N3 spindles. Features related to the slow-wave (amplitude and coupling) tended to have lower reliability than other features.

#### Reliability in sleep architecture

Among the 37 hypnogram features, 25 features (68%) had moderate reliability and 2 (5%) had good reliability; out of 248 hypnodensity features, 71 features (29%) had moderate reliability and 23 (9%) had good reliability. In both hypnogram and hypnodensity categories, a substantial number of features had poor reliability (29% and 62%, respectively), suggesting that within this population sleep architecture is highly variable from night to night.

#### Reliability in other features

Eye movement and PLMS features all had good reliability; MWT SOL had moderate or poor reliability.

### Comparison of reliability of sleep features in NT1 and NT2 populations

We found that reliability differed between NT1 and NT2 in 119 features (80 more reliable in NT1; Levene’s test, *p* < .05; Supplementary Table 2). Among these features, the following trends stood out: (1) hypnodensity features related to REM sleep onset time were more reliable in NT2 than NT1; (2) qEEG features were more reliable in NT1 than NT2; (3) MWT SOL were more reliable in NT1 than NT2.

#### Hypnodensity features

There were 58 hypnodensity features with differences in reliability between NT1 and NT2 (28 features with higher reliability in NT1; Supplementary Table 2). Many of the features were related to the onset of REM stage or mixtures that included REM (e.g. AT05 R, AT10 W•R) and indicate that REM onset was more reliable in NT2 than in NT1.

#### qEEG features

There were 37 qEEG features with higher reliability in NT1 than in NT2 and 1 feature with higher reliability in NT2 than in NT1 (Supplementary Table 2). The one feature with higher reliability in NT2 was the frequency associated with peak theta power during REM sleep. The 37 features with higher reliability in NT1 were most pronounced in the beta and delta frequency bands, but spanned all measures, frequency bands, and sleep stages (Supplementary Figure 3).

#### MWT SOL

MWT SOL was more reliable in NT1 than in NT2 (three out of four time points and the four-test mean); in each feature, reliability in NT1 was estimated at ≥0.74, while reliability in NT2 was ≤0.51.

#### Validating reliability of nPSG and MWT features in NT1 in a separate cohort

To validate our findings, we analyzed data from a separate cohort with NT1 monitored on two nights during a different clinical trial (NCT04096560). Comparing reliability estimates from the validation cohort to the NT1 population in the primary cohort, we found the correlation between reliability estimates across all features was 0.54 (95% confidence interval: 0.47, 0.60). Features with good reliability (>0.75) in the primary cohort had good reliability in the validation cohort at better-than-chance levels (82% > 0.75, 44% expected by chance, *p* < .001; 95% > 0.5, 66% expected by chance, *p* < .001; one-sided Fisher’s exact test). Out of 130 features with good reliability in the primary cohort, only six features had poor reliability (<0.5) in the validation cohort (hypnodensity: N1 AT50(w), W•N1•R max; qEEG: N3 mean beta, N3 mean gamma; MWT: 10 a.m. test and four-test mean SOL).

### Effect of study visit

We assessed whether factors related to the study visit (i.e. first [baseline], second [Week 4], or third [Week 8] visit) influenced any of the measured outcomes. Such effects could reflect, for example, placebo effects or drug washout effects on nPSG variables, including REM and NREM sleep latencies and the proportion of time spent in each sleep stage based on manually scored hypnograms, as well as SOL during the daytime MWT.

We found no study visit effects in any of the MWT SOL measurements, or sleep architecture features in NT1 (“on-placebo” versus baseline, two-tailed *t-*test, alpha = .05; Figure 1). In participants with NT2, we detected an increase in TST (effect size = 0.52, 18.4 minutes, *p* = .004), a decrease in the proportion of time awake (effect size = -0.49, -3.41%, *p* = .018), an increase in the proportion of N3 (effect size = 0.39, 2.03%, *p* = .003), and an increase of the proportion of REM sleep (effect size = 0.49, 2.05%, *p* = .037). In NT2, we also detected a decrease in SOL that was significant only at Week 8 compared with baseline (effect size = -0.577, -5.16 minutes, *p* = .005).

**Figure 1:**
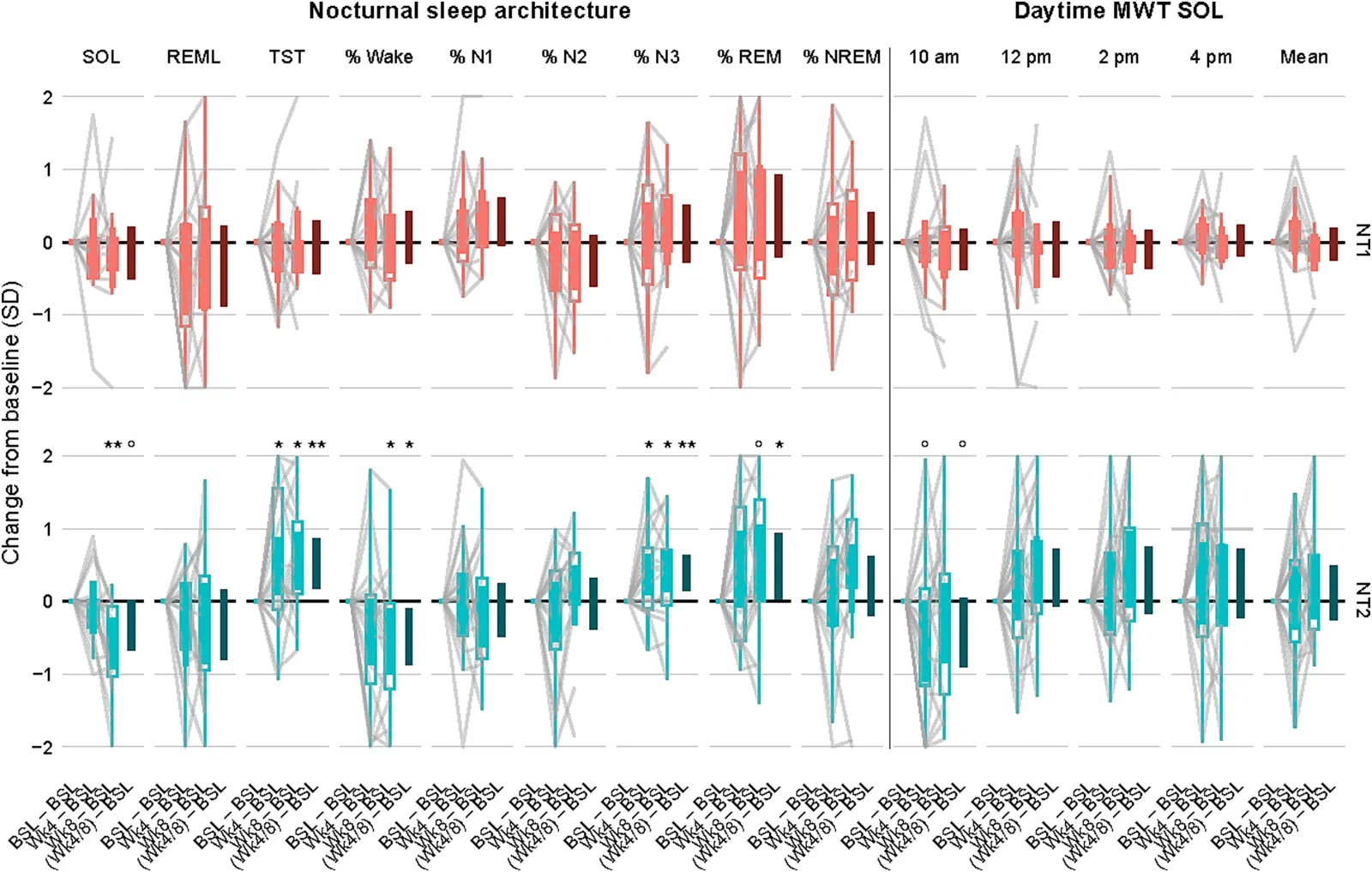
Study-visit effects in commonly measured sleep features during daytime and nighttime. IQR, interquartile range; MWT, maintenance of wakefulness test; NREM, non-rapid eye movement; NT1, narcolepsy type 1; NT2, narcolepsy type 2; REM, rapid eye movement; REML, latency from sleep onset to stage REM; SOL, sleep onset latency; TST, total sleep time. Values are shown as changes from baseline computed based on transformed observations (see Methods). Boxplots indicate median, first, and third quartiles; whiskers extend no further than the end of the box plus 1.5 IQR. Thin lines indicate values observed in each participant. Thick bands indicate 95% confidence interval of model-estimated contrasts with baseline: lighter shaded bands correspond to models with a three-level visit factor (baseline, Week 4, Week 8), and darker shaded bands correspond to models with a two-level visit factor (baseline versus “on-placebo”). °*p* < .1, ^*^p < .05, ^**^p < .01, ^***^p < .001; *p*-values corresponding to models with a three-level visit factor are adjusted within each feature using the Dunnett’s method for two tests.

To test the hypothesis that sleep stage proportions would show more pronounced study visit effects early in the night, we segmented the night into quarters and refit LMMs to the observations from each quarter. In NT1, the proportion of N1 sleep increased during the first quarter (effect size = 0.471, 1.44%, *p* = .050) and the proportion of NREM sleep decreased during the second quarter (effect size = -0.573, -3.74 %, *p* = .043; Supplementary Figure 4). In participants with NT2, the decrease in proportion of time awake was significant in the first quarter of the night (effect size = -0.882, -6.97%, *p* = .001) while the increase in proportion of time in N3 was significant in the second quarter of the night (effect size = 0.607, 3.35%, *p* = .023). We also detected a decrease in proportion of time in N1 during quarter 3 in Week 4 (effect size = -0.56, -1.77%, *p* = .048), however the effect was not replicated at Week 8.

In the remaining features (hypnogram stage transition rates, hypnodensity, qEEG, spindle, leg/eye movements), study visit effects in the whole-night features were observed in 23 features in the NT1 cohort and 13 features in the NT2 cohort (Supplementary Table 3). In NT1, three features had highly significant effects (*p* < .01): peak frequency in the sigma band during N1 (effect size = -0.348, -0.146 Hz, *p* < .001) and during Wake (effect size = -0.21, -0.102 Hz, *p* = .007), and entropy of the Wake-N3 hypnodensity mixture (effect size = 0.771, 0.0333 nats, *p* = .003). In NT2, two features had a highly significant effect: peak frequency in the theta band during N1 (effect size = 0.31, 0.154 Hz, *p* = .008) and the transition rate from N2 to Wake (effect size = -0.495, -0.00632 per epoch, *p* = .007).

### Reliable sleep features differentiating NT1 and NT2 populations

We detected diagnosis effects (differences between NT1 and NT2 within the primary cohort) in 142 features (two-tailed *t-*test, *p* < .05). To validate the detections, we compared measurements from the validation cohort (comprising only participants with NT1) to NT2 measurements from the primary cohort. Diagnosis effects were confirmed in 105 of 142 features (one-sided *t-*test, *p* < .05).

Of the features with confirmed diagnosis effects, 40 had moderate or good reliability in all three cohorts (Table 3). This set includes 8 hypnogram features, 27 hypnodensity features, 4 qEEG features, and eye movement density during REM sleep. The hypnogram features indicate N2 proportion and bout duration were reduced while wake after sleep onset (WASO) and N1 proportion were increased in NT1 relative to NT2. Hypnodensity features similarly indicated that in NT1, the N2 prevalence was reduced, while wake and N1 prevalence were increased. We also saw that the timing of Wake was important: prevalence-weighted timing of Wake had a larger effect size and smaller *p*-value than prevalence of Wake alone (without incorporating timing information). This indicated that Wake persisted later into the night in the NT1 group than the NT2 group. The hypnodensity features involving stage mixtures primarily indicated that mixtures involving Wake or N1 occurred with higher prevalence and later timing in NT1 than NT2. The 4 qEEG features that differentiated NT1 from NT2 were spread across different frequency bands and stages. The feature with the most significant effect indicated the peak delta power during N2 was lower in NT1 than in NT2 (effect size = -0.838, -1.01 μV^2^/Hz, *p* = .005). Finally, eye movement density during REM sleep was higher in NT1 than in NT2 (effect size = 0.791, 1.62 /min, *p* = .012).

**Table 3:** Reliable features that differentiate NT1 from NT2

	Diagnosis effect			Reliability	
Feature	NT1 effect (SD)	*P*-value	False discovery rate adjusted *P*-value	NT1	NT2
**Hypnogram (n = 8)**					
N2 bout duration	–0.94	.001	.011	0.73	0.71
N1 percentage	0.78	.010	.042	0.75	0.63
N2 percentage	–0.85	.003	.014	0.72	0.65
SOL	–0.64	.036	.103	0.72	0.64
SSI	0.92	.003	.014	0.74	0.73
WASO	1.05	.000	.001	0.58	0.54
N2 → N2	–1.10	.000	.001	0.54	0.83
N2 → Wake	1.18	.000	.000	0.62	0.59
**Hypnodensity (n = 27)**					
Wake prevalence	1.05	.000	.003	0.72	0.76
Wake AT50(w)	1.23	.000	.000	0.62	0.61
N1 prevalence	1.18	.000	.002	0.85	0.71
N2 prevalence	–0.99	.000	.003	0.54	0.70
Wake-N1 prevalence	1.14	.000	.002	0.83	0.74
Wake-N1 AT50	0.92	.001	.005	0.67	0.55
Wake-N1 AT50(w)	1.35	.000	.000	0.73	0.87
N1-REM prevalence	1.13	.000	.002	0.69	0.84
N1-REM AT50(w)	1.10	.000	.002	0.65	0.70
Wake-N2 AT50(w)	0.89	.006	.026	0.75	0.74
Wake-REM prevalence	1.03	.001	.005	0.86	0.81
Wake-REM AT50(w)	1.19	.000	.002	0.84	0.83
N3-REM prevalence	–0.69	.019	.059	0.68	0.53
N1-N2-REM AT50(w)	0.78	.011	.039	0.70	0.75
N2-N3-REM prevalence	–0.84	.004	.023	0.64	0.61
N2-N3-REM AT50(w)	–0.66	.031	.081	0.76	0.57
Wake-N1-N2 prevalence	0.92	.004	.020	0.73	0.67
Wake-N1-N2 AT50(w)	1.25	.000	.001	0.67	0.80
Wake-N1-N3 prevalence	0.64	.042	.104	0.81	0.72
Wake-N1-N3 AT50(w)	0.85	.007	.029	0.63	0.81
Wake-N1-REM prevalence	1.22	.000	.002	0.87	0.84
Wake-N1-REM AT50(w)	1.27	.000	.001	0.86	0.83
Wake-N2-REM AT50(w)	0.83	.011	.039	0.77	0.84
Wake-N1-N2-REM prevalence	0.90	.006	.026	0.78	0.76
Wake-N1-N2-REM AT50(w)	1.02	.002	.012	0.87	0.83
Wake-N1-N3-REM prevalence	0.70	.034	.085	0.84	0.84
Wake-N1-N3-REM AT50(w)	0.83	.012	.043	0.86	0.77
**Other (n = 1)**					
REM EM density	0.79	.012	.047	0.79	0.85
**qEEG (n = 4)**					
N1 theta, mean	0.66	.043	.352	0.78	0.76
N2 delta, peak amp	–0.84	.005	.266	0.77	0.72
N1 sigma, peak amp	–0.63	.044	.352	0.71	0.68
REM theta, peak amp	–0.81	.011	.266	0.84	0.76

AT50(w), timing-weighted prevalence; EM, eye movement; NT1, narcolepsy type 1; NT2, narcolepsy type 2; qEEG, quantitative electroencephalogram; REM, rapid eye movement; SD, standard deviation; SOL, sleep onset latency; SSI, stage shift index; WASO, wake after sleep onset.

Features with a diagnosis effect (*t-*test; *p* < .05) and moderate (reliability > 0.5) or good (reliability > 0.75) in all cohorts.

MWT test times had validated diagnosis effects and moderate or good reliability in NT1 at 12 noon, 2 p.m., and 4 p.m. (Supplementary Table 1). The 10 a.m. test time had poor reliability in the validation cohort, and all four test times had poor reliability in NT2. We did not identify any spindle features with validated diagnosis effects.

### Daytime MWT SOL

The model-based analysis above suggested that SOL during daytime MWT assessments were both reliable and aided in differentiating NT1 from NT2 (i.e. showed a diagnosis effect). However, to accommodate model fitting, observations were transformed to normalize the distributions, with effect sizes expressed as standard deviations. For clinical applicability, we additionally report raw MWT SOL values and median visit-to-visit changes in minutes (Table 4), complementing the model-based standardized effect sizes described above.

**Table 4:** MWT sleep latency at baseline and change in MWT sleep latency at Week 4 versus baseline

	Baseline SOL (minutes)			׀∆SOL ׀(minutes)		
Characteristic median (Q1, Q3)	NT1 (n = 17)	NT2 (n = 19)	*P*-value ^*^	NT1 (n = 17)	NT2 (n = 19)	*P*-value ^*^
10 a.m.	2.5 (1.0, 5.5)	6.0 (2.5, 17.5)	.037	1.0 (0.5, 4.0)	4.5 (2.0, 15.0)	.012
12 noon	1.5 (0.5, 5.0)	5.5 (1.5, 11.0)	.067	2.0 (0.5, 7.0)	5.0 (2.0, 9.5)	.2
2 p.m.	2.5 (0.5, 5.0)	5.0 (4.0, 9.0)	.037	1.5 (1.0, 4.0)	3.0 (1.5, 4.5)	.2
4 p.m.	2.0 (0.5, 5.0)	6.0 (3.0, 21.0)	.020	1.5 (0.5, 3.0)	5.0 (3.0, 14.5)	.047
Mean	2.0 (1.1, 6.4)	7.5 (3.6, 15.0)	.010	1.0 (0.5, 5.0)	5.5 (30., 10.0)	.029

MWT, maintenance of wakefulness test; NT1, narcolepsy type 1; NT2, narcolepsy type 2; SOL, sleep onset latency; ׀∆SOL׀, absolute value of change in SOL between baseline and Week 4.

^*^Wilcoxon rank sum test.

In the data analyzed here, the median SOL in NT1 was less than 3 minutes at all test times compared with at least 5 minutes for NT2 (baseline visit; *p* < .05 at 10 a.m., 2 p.m., 4 p.m., 4-test mean; Wilcoxon rank sum test; Table 4). The median absolute change in MWT SOL between the visits at baseline and Week 4 was at most 2 minutes in NT1 compared with at least 3 minutes in NT2 (*p* < .05 at 10 a.m., 4 p.m., 4-test mean; Wilcoxon rank sum test; Table 4).

We also evaluated the consistency of MWT SOL using illustrative threshold-based classifications. We evaluated two commonly used reference points: 8 minutes, which originates from earlier guideline interpretations and MSLT-based definitions [37], and 20 minutes, which has been used to reflect expected wakefulness in normative MWT studies [38]. Using a cutoff of 8 minutes [39], a majority of participants with NT1 had SOLs below the cutoff at all three visits and fewer than 30% had “mixed” results (SOLs below the cutoff at some visits and above the cutoff at others); among NT2 participants, at least 39% had mixed results (Figure 2A). Using a cutoff of 20 minutes, a majority of both participants with NT1 and NT2 had SOLs below the cutoff at all three visits, but again, more participants with NT2 than NT1 had mixed results (at most 12% NT1 with mixed results versus at least 28% NT2 with mixed results; Figure 2B). Individual observations of SOL during MWTs are given in Supplementary Figure 5. One participant with NT2 was excluded from this analysis as they did not have MWTs at 2 p.m. or 4 p.m. during the second visit.

**Figure 2:**
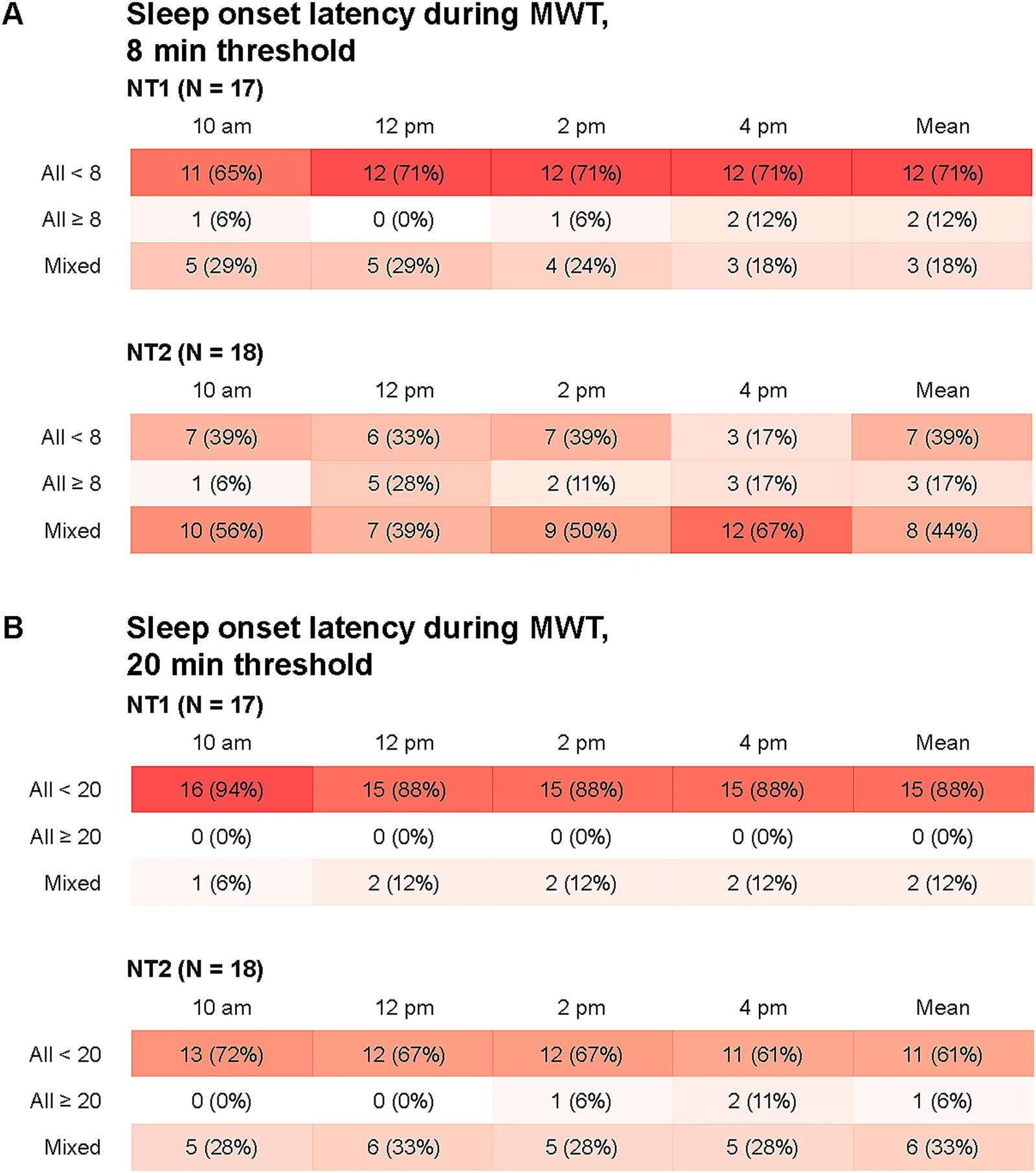
Maintenance of wakefulness test sleep onset latency threshold consistency. MWT, maintenance of wakefulness test; NT1, narcolepsy type 1; NT2, narcolepsy type 2; SOL, sleep onset latency. (A) Classification consistency based on an illustrative cutoff of 8 minutes for NT1 (top section) and NT2 (bottom section). The top row of each section shows the proportion of participants with MWT SOL <8 minutes at all three visits; the middle row shows the proportion of participants with MWT SOL ≥8 minutes at all three visits; the bottom row shows the proportion of participants with mixed classification (sometimes above threshold, sometimes below threshold). The shading indicates the percentage of participants in each category with lighter shades indicating a lower percentage and darker shades indicating a higher percentage. (B) Same as A, using an illustrative cutoff of 20 minutes.

In the validation cohort, participants took the MWT on only two visits (at baseline and Week 8). Consistent with the original NT1 cohort, approximately 70% of participants in the validation cohort had MWT SOLs below 8 minutes at both visits; approximately 90% of participants had MWT SOLs below 20 minutes at both visits (Supplementary Figure 6).

## Discussion

Our study addresses an important gap in the literature, namely the limited availability of data comprising multiple nighttime sleep and daytime MWT recordings over a short time interval in drug-free individuals with narcolepsy. Results from participants in placebo arms of clinical trials showed that spectral analyses of EEG features (spindles, qEEG) had the highest test–retest reliability but little differentiation between diagnoses (NT1 versus NT2). In contrast, aspects of nocturnal sleep architecture (e.g. proportion of time in Wake or N1) and MWT SOL showed good differentiation between diagnoses, confirming their utility in clinical practice, although these features are not as reliable as the spectral features. Hypnodensity Wake-REM mixtures showed good reliability and differed by diagnosis of NT1 versus NT2, suggesting that these features may be valuable for differential diagnosis. We also showed that reliability is lower in NT2 compared with NT1 in qEEG features and daytime MWT SOL, suggesting that nighttime sleep and daytime wakefulness in this population are less consistent from one visit to another.

We identified several features related to nocturnal sleep architecture that differed between NT1 and NT2 and had good test–retest reliability in both diagnoses. Consistent with previous studies, these features indicated people with NT1 had more WASO, spent a greater percentage of time in N1, had more fragmented sleep, and spent a lower percentage of time in N2 with shorter N2 bouts compared with people with NT2 [5, 10, 40, 41]. We also saw evidence that Wake persists later into the night in NT1 compared with NT2 (as measured by time-weighted hypnodensity prevalence measures), suggesting that nocturnal wakefulness in NT2 may reflect trouble falling asleep, while in NT1, sleep disruptions continue throughout the night.

MWT SOLs are key features of clinical interest, and their reliability has not been characterized before. The MWT showed good differentiation between NT1 and NT2 diagnoses, with moderate-to-good reliability in NT1. In NT2, the mean MWT SOL was less reliable than in NT1. The 4 p.m. MWT test time (8 hours post-dose) was the most stable for NT1, suggesting that afternoon testing is the most reliable. We did not detect significant visit effects in either diagnosis group, indicating first-visit effects are minimal.

To aid clinical interpretation, we additionally evaluated the consistency of MWT SOL using threshold-based classifications. Proposed thresholds for pathological responses vary across studies, and no universally accepted cutoff exists for the MWT. In the 2005 AASM practice parameters, abnormal mean sleep latency was defined as at least one epoch <8 minutes and values between 8 and 40 minutes were considered of uncertain significance [37]; however, this threshold was not retained in the subsequent 2021 update [42]. Thus, while a cutoff of 8 minutes is sometimes applied in clinical contexts, its origin lies in earlier guideline interpretations and in definitions established for the MSLT, and its applicability to MWT remains uncertain. In contrast, a normative study of MWT sleep latency reported a threshold of 20 minutes reflects expected wakefulness in healthy individuals [38].

In this context, we present threshold-based analyses primarily as an interpretive framework rather than a definitive classification of abnormality. Consistent with the continuous analyses, threshold-based analyses indicated participants with NT2 more often exhibited SOLs on different sides of the threshold at different visits, reflecting greater within-subject variability. These findings highlight that reliance on fixed thresholds may be particularly limited in NT2, and that continuous SOL values and changes over time may provide a more informative assessment of daytime wakefulness and its reliability.

These results support the use of MWT assessments, particularly in the afternoon, as endpoints in clinical trials of participants with NT1. Due to less reliable results with NT2, further analyses are warranted to confirm the best use of MWT in clinical trials for this diagnosis. In addition, the different levels of MWT reliability in NT1 and NT2 should be considered in sensitivity analyses during clinical trial design.

Our analysis of study visit effects showed limited evidence of systematic changes across visits for most features. We did not detect significant visit effects in MWT SOL or the majority of nocturnal sleep measures, suggesting that study visit-related factors did not materially influence the estimation of test–retest reliability. These findings support the interpretation that observed variability primarily reflects within-participant variability rather than systematic adaptation across visits.

In NT2, modest changes consistent with a first-night effect were observed, including increased TST and reduced wakefulness after the baseline night. First-night effects are well described in sleep laboratory studies [43–45], including in NT1 [43], although we did not observe similar effects in NT1 here. Differences in prior laboratory exposure, intrinsic sleep regulation, or sensitivity to environmental factors may contribute to this pattern, but could not be evaluated in the present study. These findings should be interpreted cautiously given the modest sample size.

REM sleep abnormalities are a hallmark feature of narcolepsy, with REM sleep latency less than 15 minutes being a diagnostic criteria for both NT1 and NT2 in ICSD-3-TR [39]. However, REM sleep latency derived from visually scored hypnograms showed poor reliability in both NT1 and NT2. In contrast, REM sleep latency estimated from hypnodensities (defined as the time to 5% cumulative REM sleep) demonstrated poor reliability in NT1 but good reliability in NT2 (REM AT05, Supplementary Table 2). The discrepancy between visually scored and hypnodensity-derived REM latency reliability may reflect differences in how each metric captures REM onset. In narcolepsy, REM sleep scoring can be particularly challenging due to instability of sleep architecture, frequent transitions between wake, NREM, and REM sleep, and the presence of dissociated states reflecting state boundary dyscontrol [43, 46–49]. As a result, identification of REM onset based on visually scored hypnograms—which depends on a single discrete epoch—can be sensitive to staging ambiguity [50, 51]. Consistent with this, previous work has recommended requiring a sustained period of REM sleep (e.g. >30 seconds) to improve reproducibility of REM identification and reduce interpretation bias in ambiguous cases [52].

In contrast, hypnodensity-derived REM onset (time to 5% cumulative REM probability) captures a gradual probabilistic accumulation of REM features and does not rely on a single discrete staging decision, making it less sensitive to staging ambiguity. Although both NT1 and NT2 are characterized by early REM episodes, REM sleep in NT2 is generally less disrupted than in NT1 [48, 49], with lower REM eye movement density and more preserved muscle atonia [53, 54]. This may explain why hypnodensity-based REM onset measures showed higher test–retest stability in NT2 in our data, though these findings should be interpreted cautiously and warrant further investigation.

More recently, dREMs have been identified as a biomarker of NT1 [6, 9, 52]. As observed for REM sleep latency, the number of dREMs also showed poor night-to-night reliability. Although strong test–retest reliability is generally expected for biomarkers, the instability of these two established NT1 markers suggests that diagnostically relevant information may arise from features that are intrinsically dynamic. The variability in nocturnal SOREMPs and dREMs may reflect both genuine biological fluctuation, similar to the episodic nature of cataplexy, and methodological influences, including sensitivity to scoring decisions and variability in REM microstructure. Episodes of cataplexy are decisive for NT1 diagnosis but do not occur on a predictable daily schedule. Similarly, a single-night sleep study may miss a rare or irregular REM intrusion such as a SOREMP or direct REM episode. Unlike cataplexy, which individuals can describe to a physician, nocturnal REM intrusions are imperceptible without objective monitoring, underscoring a need for repeated or longitudinal measurement. As wearable sleep-tracking technologies advance and multi-night home sleep testing becomes more feasible, it may be possible to capture these infrequent REM events outside the laboratory, potentially enabling earlier detection despite their inherent night-to-night variability.

Among the top features in terms of reliability and diagnostic potential between NT1 and NT2 were hypnodensity features related to prevalence and timing of mixtures involving wake and REM. These features may offer an alternative measure of REM sleep onset that relies on a probabilistic description of sleep and thus naturally describes sleep dissociation. Currently, automated sleep staging that often generates hypodensities is not commonly used in the clinic. However, this work suggests that automated staging for characterizing mixed-states or sleep-state uncertainty could provide valuable information to clinicians.

Our study also showed that reliability was lower in NT2 compared with NT1 across multiple feature domains, suggesting that nighttime sleep and daytime wakefulness in this population are less consistent from one visit to another. CSF orexin measurements were not systematically available in the NT2 cohort, precluding assessment of reliability as a function of orexin level. This is particularly relevant given that individuals with NT2 may exhibit a range of CSF orexin levels, from normal to intermediate concentrations [2–4, 55], which may contribute to the greater variability observed in this group. In some cases, individuals with a NT2 phenotype are actually individuals with NT1, orexin deficient and without cataplexy. Future studies evaluating test–retest reliability within subgroups defined by orexin level could help clarify whether underlying biological heterogeneity contributes to the increased variability seen in NT2.

There are several limitations of this study. First, the primary cohort comprised a relatively small sample of 36 individuals. The limited population means the results may exhibit cohort-specific tendencies. To combat this, we validated our results in a separate cohort of individuals with NT1 who participated in the placebo arm of an earlier clinical trial. However, this validation cohort comprised only participants with NT1. An equivalent validation cohort of participants with NT2, which was unfortunately not available at the time of study, would have provided a more robust population with which to validate all results.

Second, the population includes only NT1 and NT2 diagnoses, and no healthy control participants. Because our estimates of reliability depend on the total variance in the observations, the limited population, which does not include healthy controls or individuals with other sleep disorders, will show more limited variance than a broader population, thus impacting reliability estimates. Future investigations could test the reproducibility of these results, for example by analyzing placebo arms from additional clinical trials in narcolepsy and other sleep disorders.

Third, participants were enrolled according to defined inclusion/exclusion criteria and may represent a more homogenous population than would be seen in the clinic. Thus, broadening the scope to include individuals on medication, with a wider set of clinical characteristics, or with different intervals between visits could assess reliability under conditions more common in the clinic.

Finally, given the number of features evaluated (440 total, with 142 showing diagnosis effects at *p* < .05) relative to the sample size of 36 participants, findings should be interpreted cautiously despite the application of the Benjamini-Hochberg false discovery rate correction within each feature category and analysis task. The present results are intended as hypothesis-generating and should be confirmed in larger cohorts. Although no illness coinciding with nPSG recordings was identified, minor adverse events were reported in a small number of participants and may have contributed to night-to-night variability in some measures.

In conclusion, we showed that spectral features (spindles, qEEG) had the best reliability but rarely differed between NT1 and NT2. In contrast, SOL from MWT and selected hypnodensity features had good reliability and differed by diagnosis, indicating that these features may be particularly useful in differential diagnosis of NT1 versus NT2. We also showed that reliability is lower in NT2 (versus NT1) across multiple features, suggesting that sleep in the NT2 population is less consistent visit-to-visit.

## Supplementary Material

Supplementary-material_zsag180

## Data Availability

Deidentified data and materials supporting the findings of this study are available from the corresponding author upon reasonable request following publication.
