# Supplementary material for: Nocturnal features and daytime characteristics in narcolepsy: reliability and diagnostic relevance for NT1 Versus NT2
Source: Sleep. 2026 Jul 7;49(10):zsag180. doi: 10.1093/sleep/zsag180 (PMC13658287; doi:10.1093/sleep/zsag180)
Supplement: Supplementary file 1 [file supplementary-material_zsag180.zip › Test_-_retest_reproducibility_supplementary_material_2Apr2026_zsag180.docx]

**Supplementary Materials**

**Nocturnal Features and Daytime Characteristics in Narcolepsy: Reliability and Diagnostic Relevance for NT1 vs NT2**

Emily Schlafly, Marta Karas, Yishu Gong, Lucie Barateau, Brian Tracey, Dmitri Volfson, and Yves Dauvilliers^*^

*****Corresponding author. Yves Dauvilliers, National Reference Centre for Narcolepsy, CHU Gui de Chauliac, Montpellier, France; 80, avenue Augustin Fliche, 34295 Montpellier Cedex 5, France. Phone: +33467337478. Email: [ydauvilliers@yahoo.fr](mailto:ydauvilliers@yahoo.fr)

**Supplementary Methods:** YASA parameters	3

**Supplementary Table 1.** Test-retest reliability in all features.	4

**Supplementary Table 2.** Comparison of test-retest reliability in NT1 vs NT2 for different features (N=119, Levene’s test, p<0.05).	25

**Supplementary Table 3.** Study visit effects for hypnodensity, qEEG, spindle, and movement features.	27

**Supplementary Figure 1.** qEEG feature reliability estimated from the combined NT1 and NT2 cohorts.	30

**Supplementary Figure 2.** Spindle feature reliability estimated from the combined NT1 and NT2 cohorts.	31

**Supplementary Figure 3.** Comparison of qEEG feature reliability in NT1 versus NT2.	32

**Supplementary Figure 4.** Change from baseline in hypnogram stage percentages in each quarter-night.	33

**Supplementary Figure 5.** MWT SOL in each participant and test.	35

**Supplementary Figure 6.** Maintenance of wakefulness test sleep onset latency in the NT1 validation cohort.	37

**Supplementary Methods:**

YASA parameters

Eye movements were detected using YASA with default parameter settings. Spindles were detected using YASA with default settings except for the broadband range parameter. Because YASA’s decision rule includes a relative power criterion computed over a user-defined broadband range, we restricted this range to 5–30 Hz (rather than 1–30 Hz) to minimize the influence of low-frequency slow-wave activity that is prominent in N3, and which can inflate broadband power estimates, thereby altering the relative power in the spindle band.

We obtained slow-wave features (coupling and amplitude) following the procedure outlined in the Luna toolbox (<http://zzz.bwh.harvard.edu>). We bandpass filtered the signal to 0.3-1.5 Hz. We then computed the instantaneous amplitude of the slow-wave component and a robust z-scored variant of the slow-wave component. We declared a slow-wave coupled to a spindle if the absolute amplitude of the z-scored component exceeded 2 at the time of the spindle peak.

**Supplementary Table 1.** Test-retest reliability in all features.

| **Hypnogram features** | | | | | | | | | | | | | | | | | |
| --- | --- | --- | --- | --- | --- | --- | --- | --- | --- | --- | --- | --- | --- | --- | --- | --- | --- |
|  | **Feature** |  | **Statistics** | | **Reliability** | | | |  | **Diagnosis effect** | | | |  | **Study visit effect** | | |
|  |  |  | Median [IQR] | Δ | Est. (95% CI) | *p* | FDR | Val. |  | NT1 eff. (SD) | *p* | FDR | Val. |  | Est. | *p* | FDR |
| Whole-night | | | | | | | | | | | | | | | | | |
|  | % NREM | NT1: NT2: | 66.1 [60.1, 75.9] 65.2 [63.2, 70.5] | 3.71 5.03 | 0.707 (0.493, 0.816) 0.510 (0.193, 0.681) | **0.049** | 0.178 | 0.898 |  | -0.18 | 0.476 |  | — |  | 0.05 0.21 | 0.793 0.311 |  |
|  | REM latency, min |  | 76.0 [16.9, 108] 78.5 [57.0, 110] | 43.0 19.0 | 0.273 (-0.245, 0.540) 0.358 (-0.077, 0.586) | 0.576 |  | 0.403 |  | -0.29 | 0.263 |  | — |  | -0.33 -0.32 | 0.233 0.186 |  |
|  | SOL, min |  | 4.50 [1.50, 8.12] 10.5 [3.00, 20.5] | 3.00 3.50 | 0.719 (0.509, 0.825) 0.643 (0.404, 0.770) | 0.148 |  | 0.943 |  | -0.64 | **0.036** | 0.103 | ✓ |  | -0.15 -0.32 | 0.398 0.071 |  |
|  | SSI, Pr |  | 0.150 [0.131, 0.183] 0.133 [0.126, 0.176] | 0.0289 0.0188 | 0.738 (0.543, 0.835) 0.732 (0.555, 0.826) | 0.731 |  | 0.785 |  | 0.92 | **0.003** | **0.014** | ✓ |  | 0.03 -0.18 | 0.888 0.273 |  |
|  | TST, min |  | 437 [407, 463] 420 [389, 442] | 17.5 16.0 | 0.697 (0.475, 0.810) 0.569 (0.287, 0.720) | 0.143 |  | 0.934 |  | -0.09 | 0.757 |  | — |  | -0.07 0.52 | 0.710 **0.004** | 0.083 |
|  | WASO, min |  | 51.0 [31.5, 93.9] 45.5 [32.5, 61.0] | 33.2 19.5 | 0.581 (0.270, 0.737) 0.538 (0.207, 0.698) | 0.766 |  | 0.845 |  | 1.05 | **0.000** | **0.001** | ✓ |  | -0.12 -0.47 | 0.590 **0.018** | 0.133 |
|  | dREM, N |  | 1.00 [0.00, 2.75] 1.00 [0.00, 3.00] | 2.00 1.00 | 0.416 (-0.007, 0.632) 0.390 (-0.014, 0.607) | 0.202 |  | 0.524 |  | 0.67 | **0.013** | **0.047** | ✓ |  | 0.48 -0.13 | 0.052 0.594 |  |
| N1 | | | | | | | | | | | | | | | | | |
|  | % | NT1: NT2: | 6.66 [4.93, 10.3] 6.43 [5.73, 8.70] | 1.42 2.10 | 0.749 (0.564, 0.843) 0.626 (0.380, 0.757) | 0.200 |  | 0.540 |  | 0.78 | **0.010** | **0.042** | ✓ |  | 0.28 -0.12 | 0.102 0.532 |  |
|  | Bout duration, min |  | 0.838 [0.817, 0.943] 0.871 [0.756, 1.05] | 0.116 0.126 | 0.586 (0.294, 0.738) 0.726 (0.542, 0.824) | 0.223 |  | 0.115 |  | 0.16 | 0.599 |  | — |  | 0.40 0.14 | 0.052 0.398 |  |
|  | N1→N1, Pr |  | 0.404 [0.388, 0.470] 0.426 [0.339, 0.525] | 0.0753 0.0909 | 0.639 (0.385, 0.772) 0.758 (0.598, 0.844) | 0.319 |  | 0.330 |  | 0.16 | 0.601 |  | — |  | 0.36 0.13 | 0.061 0.378 |  |
|  | N1→N2, Pr |  | 0.354 [0.332, 0.431] 0.333 [0.291, 0.387] | 0.0574 0.0721 | 0.807 (0.664, 0.879) 0.683 (0.477, 0.794) | 0.296 |  | 0.299 |  | -0.18 | 0.576 |  | — |  | -0.23 0.14 | 0.132 0.415 |  |
|  | N1→REM, Pr |  | 0.0794 [0.0569, 0.101] 0.0755 [0.0164, 0.129] | 0.0287 0.0456 | 0.728 (0.527, 0.829) 0.370 (-0.035, 0.589) | **0.005** | 0.077 | 0.915 |  | -0.52 | 0.069 |  | — |  | -0.15 0.35 | 0.386 0.138 |  |
|  | N1→Wake, Pr |  | 0.0929 [0.0635, 0.126] 0.0882 [0.0492, 0.140] | 0.0443 0.0692 | 0.677 (0.440, 0.797) 0.080 (-0.371, 0.399) | **0.006** | 0.077 | 0.614 |  | 0.58 | **0.024** | 0.082 | ✓ |  | 0.11 -0.61 | 0.581 **0.027** | 0.165 |
| N2 | | | | | | | | | | | | | | | | | |
|  | % | NT1: NT2: | 46.1 [39.4, 57.4] 52.2 [48.1, 53.9] | 5.95 5.21 | 0.719 (0.513, 0.823) 0.646 (0.416, 0.770) | 0.347 |  | 0.970 |  | -0.85 | **0.003** | **0.014** | ✓ |  | -0.26 -0.03 | 0.156 0.860 |  |
|  | Bout duration, min |  | 4.73 [3.79, 6.14] 6.29 [4.71, 7.52] | 0.738 1.09 | 0.725 (0.525, 0.827) 0.713 (0.525, 0.814) | 0.716 |  | 0.962 |  | -0.94 | **0.001** | **0.011** | ✓ |  | -0.03 0.26 | 0.868 0.116 |  |
|  | N2→N1, Pr |  | 0.0216 [0.0189, 0.0297] 0.0245 [0.0103, 0.0271] | 0.0113 0.0103 | 0.563 (0.238, 0.726) 0.421 (0.068, 0.629) | 0.315 |  | 0.141 |  | 0.77 | **0.006** | **0.027** | ✓ |  | 0.26 -0.07 | 0.253 0.781 |  |
|  | N2→N2, Pr |  | 0.895 [0.869, 0.920] 0.920 [0.894, 0.935] | 0.0147 0.0175 | 0.537 (0.197, 0.709) 0.826 (0.709, 0.888) | **0.001** | **0.049** | 0.756 |  | -1.10 | **0.000** | **0.001** | ✓ |  | -0.10 0.09 | 0.673 0.496 |  |
|  | N2→N3, Pr |  | 0.0279 [0.0202, 0.0332] 0.0226 [0.0133, 0.0300] | 0.00728 0.00650 | 0.452 (0.017, 0.662) 0.729 (0.542, 0.827) | 0.055 |  | 0.00 |  | 0.44 | 0.155 |  | — |  | 0.11 0.13 | 0.655 0.427 |  |
|  | N2→REM, Pr |  | 0.00871 [0.00604, 0.0113] 0.00998 [0.00598, 0.0133] | 0.00217 0.00342 | 0.518 (0.174, 0.695) 0.729 (0.543, 0.827) | 0.102 |  | 0.00 |  | -0.08 | 0.791 |  | — |  | -0.22 0.08 | 0.331 0.632 |  |
|  | N2→Wake, Pr |  | 0.0324 [0.0216, 0.0516] 0.0257 [0.0187, 0.0348] | 0.0102 0.0123 | 0.615 (0.334, 0.759) 0.595 (0.328, 0.737) | 0.738 |  | 0.800 |  | 1.18 | **0.000** | **0.000** | ✓ |  | 0.07 -0.49 | 0.743 **0.007** | 0.084 |
| N3 | | | | | | | | | | | | | | | | | |
|  | % | NT1: NT2: | 12.1 [8.81, 15.1] 7.29 [2.60, 10.0] | 4.11 2.50 | 0.639 (0.380, 0.772) 0.790 (0.653, 0.863) | 0.055 |  | 0.830 |  | 0.37 | 0.231 |  | — |  | 0.12 0.39 | 0.550 **0.003** | 0.083 |
|  | Bout duration, min |  | 4.08 [2.81, 6.14] 3.72 [1.43, 5.37] | 2.59 2.23 | 0.403 (-0.024, 0.623) 0.641 (0.395, 0.770) | 0.100 |  | 0.315 |  | 0.14 | 0.614 |  | — |  | 0.05 0.26 | 0.840 0.159 |  |
|  | N3→N2, Pr |  | 0.108 [0.0734, 0.153] 0.120 [0.0946, 0.347] | 0.0436 0.0672 | 0.753 (0.569, 0.846) 0.807 (0.667, 0.878) | 0.421 |  | 0.755 |  | -0.56 | 0.068 |  | — |  | -0.07 -0.18 | 0.668 0.212 |  |
|  | N3→N3, Pr |  | 0.875 [0.821, 0.919] 0.865 [0.623, 0.905] | 0.0513 0.0638 | 0.738 (0.541, 0.837) 0.802 (0.659, 0.875) | 0.488 |  | 0.812 |  | 0.47 | 0.123 |  | — |  | 0.01 0.22 | 0.946 0.120 |  |
|  | N3→Wake, Pr |  | 0.0168 [0.00745, 0.0233] 0.00498 [0.00, 0.0247] | 0.00886 0.0108 | 0.270 (-0.266, 0.540) 0.318 (-0.170, 0.567) | 0.813 |  | 0.374 |  | 0.46 | 0.081 |  | — |  | 0.15 -0.25 | 0.599 0.335 |  |
| REM | | | | | | | | | | | | | | | | | |
|  | % | NT1: NT2: | 14.6 [12.7, 18.0] 17.4 [15.9, 19.2] | 5.78 4.63 | 0.222 (-0.280, 0.508) 0.362 (-0.073, 0.588) | 0.213 |  | 0.698 |  | -0.50 | **0.044** | 0.115 | ✗ |  | 0.36 0.49 | 0.204 **0.036** | 0.192 |
|  | Bout duration, min |  | 5.53 [4.64, 9.44] 6.72 [4.28, 10.7] | 1.30 2.79 | 0.703 (0.469, 0.817) 0.397 (-0.013, 0.612) | **0.017** | 0.122 | 0.800 |  | -0.38 | 0.191 |  | — |  | 0.12 -0.15 | 0.527 0.525 |  |
|  | REM→N1, Pr |  | 0.0291 [0.0150, 0.0412] 0.0326 [0.0133, 0.0485] | 0.0121 0.0132 | 0.607 (0.322, 0.752) 0.460 (0.109, 0.649) | 0.349 |  | 0.852 |  | -0.29 | 0.314 |  | — |  | -0.27 0.10 | 0.191 0.637 |  |
|  | REM→N2, Pr |  | 0.00656 [0.00572, 0.0185] 0.0117 [0.00, 0.0164] | 0.0124 0.00350 | 0.225 (-0.294, 0.509) 0.614 (0.360, 0.749) | **0.020** | 0.122 | 0.184 |  | -0.14 | 0.605 |  | — |  | -0.17 0.05 | 0.562 0.779 |  |
|  | REM→REM, Pr |  | 0.912 [0.898, 0.947] 0.926 [0.883, 0.953] | 0.0119 0.0345 | 0.690 (0.459, 0.806) 0.542 (0.234, 0.704) | 0.117 |  | 0.666 |  | -0.40 | 0.181 |  | — |  | 0.06 0.15 | 0.757 0.477 |  |
|  | REM→Wake, Pr |  | 0.0413 [0.0314, 0.0458] 0.0275 [0.0127, 0.0492] | 0.0166 0.0196 | 0.677 (0.429, 0.800) 0.561 (0.264, 0.716) | 0.616 |  | 0.0252 |  | 0.88 | **0.002** | **0.014** | ✓ |  | 0.03 -0.30 | 0.878 0.131 |  |
| Wake | | | | | | | | | | | | | | | | | |
|  | % | NT1: NT2: | 15.2 [7.40, 24.8] 11.8 [8.44, 19.0] | 4.79 3.33 | 0.722 (0.518, 0.825) 0.489 (0.154, 0.668) | 0.057 |  | 0.899 |  | 0.48 | 0.085 |  | — |  | 0.07 -0.49 | 0.700 **0.017** | 0.133 |
|  | Bout duration, min |  | 1.60 [1.02, 5.24] 2.01 [1.51, 4.11] | 0.642 0.869 | 0.612 (0.335, 0.755) 0.412 (0.027, 0.618) | 0.154 |  | 0.798 |  | -0.13 | 0.609 |  | — |  | -0.38 -0.16 | 0.059 0.490 |  |
|  | Wake→N1, Pr |  | 0.340 [0.131, 0.415] 0.172 [0.138, 0.229] | 0.126 0.0935 | 0.446 (0.038, 0.652) 0.363 (-0.097, 0.596) | 0.779 |  | 0.540 |  | 0.23 | 0.374 |  | — |  | 0.16 0.35 | 0.528 0.170 |  |
|  | Wake→N2, Pr |  | 0.0607 [0.0246, 0.0933] 0.0429 [0.0284, 0.0984] | 0.0199 0.0249 | 0.711 (0.496, 0.820) 0.347 (-0.075, 0.574) | **0.016** | 0.122 | 0.928 |  | -0.61 | **0.028** | 0.086 | ✓ |  | -0.32 0.39 | 0.083 0.099 |  |
|  | Wake→REM, Pr |  | 0.0162 [0.00168, 0.0311] 0.0164 [0.00847, 0.0376] | 0.0250 0.0429 | 0.533 (0.207, 0.704) -0.157 (-0.902, 0.247) | **0.025** | 0.130 | 0.555 |  | -0.17 | 0.473 |  | — |  | 0.34 0.31 | 0.123 0.332 |  |
|  | Wake→Wake, Pr |  | 0.590 [0.460, 0.831] 0.721 [0.535, 0.805] | 0.147 0.142 | 0.457 (0.059, 0.659) 0.126 (-0.405, 0.430) | 0.058 |  | 0.558 |  | 0.16 | 0.502 |  | — |  | -0.09 -0.40 | 0.725 0.141 |  |
| *Statistics*: Median and IQR of observations at baseline, Δ indicates median absolute difference between observations at baseline and week 4. *Reliability*: Estimated reliability with 95% confidence intervals; *p*, the *p*-value of Levene's test; Val., the estimated reliability in the validation cohort. *Diagnosis effect*: Effect size (NT2 versus NT1) in standardized units; *p*, the *p*-value of a t-test; Val., a check (✓) indicates the diagnosis effect is significant in the validation cohort, an ex (✗) indicates the diagnosis effect is not significant in the validation cohort, a dash (—) indicates validation of the diagnosis effect was not tested. *Study visit effect*: Comparison of values at baseline and while "on-placebo"; *p*, the *p*-value of a t-test; *FDR*: The false discovery rate (FDR) adjusted *p*-value. For each feature, results for NT1 are shown on the first line and NT2 are shown in the second line. | | | | | | | | | | | | | | | | | |

| **Hypnodensity features** | | | | | | | | | | | | | | | | | |
| --- | --- | --- | --- | --- | --- | --- | --- | --- | --- | --- | --- | --- | --- | --- | --- | --- | --- |
|  | **Feature** |  | **Statistics** | | **Reliability** | | | |  | **Diagnosis effect** | | | |  | **Study visit effect** | | |
|  |  |  | Median [IQR] | Δ | Est. (95% CI) | *p* | FDR | Val. |  | NT1 eff. (SD) | *p* | FDR | Val. |  | Est. | *p* | FDR |
| N1 | | | | | | | | | | | | | | | | | |
|  | AT05, min | NT1: NT2: | 25.0 [9.75, 85.8] 8.00 [3.00, 22.0] | 17.5 5.00 | 0.247 (-0.293, 0.524) 0.825 (0.699, 0.889) | **0.000** | **0.003** | 0.00 |  | 0.86 | **0.002** | **0.013** | ✓ |  | 0.03 -0.20 | 0.930 0.139 |  |
|  | AT10, min |  | 126 [88.3, 189] 80.0 [28.0, 143] | 44.5 41.0 | 0.473 (0.083, 0.669) 0.578 (0.294, 0.728) | 0.558 |  | 0.418 |  | 0.94 | **0.001** | **0.005** | ✓ |  | -0.15 -0.41 | 0.524 **0.038** | 0.980 |
|  | AT30, min |  | 371 [312, 437] 319 [234, 360] | 85.0 90.0 | 0.686 (0.463, 0.801) 0.321 (-0.118, 0.558) | **0.036** | 0.170 | 0.391 |  | 0.91 | **0.000** | **0.005** | ✓ |  | -0.11 -0.29 | 0.555 0.235 |  |
|  | AT50(w), min |  | 37.5 [22.3, 54.9] 22.3 [14.4, 34.1] | 5.81 9.62 | 0.823 (0.693, 0.889) 0.769 (0.614, 0.851) | 0.476 |  | 0.464 |  | 1.39 | **0.000** | **0.000** | ✓ |  | 0.09 -0.20 | 0.523 0.178 |  |
|  | AT50, min |  | 583 [524, 635] 500 [399, 612] | 70.5 134 | 0.718 (0.508, 0.824) 0.351 (-0.068, 0.577) | **0.003** | **0.045** | 0.374 |  | 0.96 | **0.000** | **0.003** | ✓ |  | -0.18 -0.23 | 0.314 0.337 |  |
|  | Entropy, nats |  | 4.02 [3.99, 4.03] 4.02 [3.98, 4.04] | 0.0265 0.0246 | 0.325 (-0.145, 0.574) 0.333 (-0.107, 0.568) | 0.678 |  | 0.516 |  | -0.13 | 0.635 |  | — |  | -0.31 0.04 | 0.239 0.878 |  |
|  | Max, % |  | 87.4 [86.6, 89.8] 84.4 [79.7, 88.5] | 2.61 6.56 | 0.410 (-0.031, 0.632) -0.197 (-1.054, 0.217) | **0.025** | 0.132 | 0.182 |  | 0.42 | 0.056 |  | — |  | -0.30 -0.15 | 0.242 0.643 |  |
|  | Prevalence, % |  | 6.14 [4.72, 8.49] 5.03 [3.86, 5.85] | 0.683 1.45 | 0.853 (0.745, 0.908) 0.708 (0.499, 0.814) | 0.128 |  | 0.629 |  | 1.18 | **0.000** | **0.002** | ✓ |  | 0.08 -0.19 | 0.555 0.251 |  |
| N2 | | | | | | | | | | | | | | | | | |
|  | AT05, min | NT1: NT2: | 46.5 [36.5, 67.5] 37.0 [32.0, 58.0] | 15.5 14.0 | -0.074 (-0.803, 0.319) 0.691 (0.474, 0.803) | **0.009** | 0.074 | 0.00 |  | 0.49 | 0.056 |  | — |  | -0.04 0.02 | 0.897 0.901 |  |
|  | AT10, min |  | 116 [96.8, 137] 92.0 [85.0, 120] | 15.5 33.0 | 0.618 (0.337, 0.760) 0.171 (-0.437, 0.461) | **0.047** | 0.205 | 0.0990 |  | 0.32 | 0.235 |  | — |  | -0.33 0.19 | 0.107 0.493 |  |
|  | AT30, min |  | 301 [287, 339] 326 [288, 341] | 32.5 53.0 | 0.421 (0.010, 0.634) 0.447 (0.083, 0.642) | 0.819 |  | 0.566 |  | -0.04 | 0.884 |  | — |  | -0.31 -0.29 | 0.218 0.191 |  |
|  | AT50(w), min |  | 229 [205, 264] 245 [225, 261] | 19.1 28.3 | 0.599 (0.310, 0.747) 0.673 (0.458, 0.788) | 0.420 |  | 0.768 |  | -0.50 | 0.076 |  | — |  | -0.18 0.22 | 0.391 0.191 |  |
|  | AT50, min |  | 498 [471, 554] 486 [456, 513] | 44.5 48.0 | 0.593 (0.280, 0.748) 0.307 (-0.147, 0.549) | 0.121 |  | 0.279 |  | 0.18 | 0.532 |  | — |  | -0.15 0.21 | 0.523 0.393 |  |
|  | Entropy, nats |  | 3.84 [3.81, 3.88] 3.89 [3.86, 3.92] | 0.0322 0.0252 | 0.567 (0.245, 0.729) 0.399 (0.007, 0.609) | 0.115 |  | 0.318 |  | -0.36 | 0.175 |  | — |  | 0.22 -0.21 | 0.339 0.355 |  |
|  | Max, % |  | 99.1 [98.9, 99.2] 99.1 [98.7, 99.1] | 0.153 0.283 | 0.702 (0.486, 0.812) 0.644 (0.399, 0.771) | 0.794 |  | 0.231 |  | -0.10 | 0.751 |  | — |  | -0.21 0.21 | 0.235 0.268 |  |
|  | Prevalence, % |  | 45.2 [41.2, 48.8] 51.5 [46.8, 55.0] | 5.56 3.07 | 0.539 (0.186, 0.714) 0.704 (0.508, 0.808) | 0.195 |  | 0.952 |  | -0.99 | **0.000** | **0.003** | ✓ |  | -0.03 0.10 | 0.887 0.529 |  |
| N3 | | | | | | | | | | | | | | | | | |
|  | AT05, min | NT1: NT2: | 49.0 [36.0, 64.8] 56.0 [37.0, 92.0] | 23.5 13.0 | 0.576 (0.255, 0.735) 0.375 (-0.065, 0.601) | 0.725 |  | 0.00 |  | -0.30 | 0.309 |  | — |  | 0.05 -0.18 | 0.834 0.457 |  |
|  | AT10, min |  | 58.0 [43.8, 78.0] 67.0 [53.0, 101] | 19.5 23.0 | 0.574 (0.247, 0.735) 0.379 (-0.059, 0.604) | 0.398 |  | 0.00 |  | -0.37 | 0.207 |  | — |  | 0.04 -0.19 | 0.863 0.450 |  |
|  | AT30, min |  | 117 [94.0, 135] 132 [106, 224] | 35.0 55.0 | 0.566 (0.241, 0.728) 0.367 (-0.067, 0.595) | 0.285 |  | 0.153 |  | -0.44 | 0.119 |  | — |  | -0.07 0.14 | 0.758 0.576 |  |
|  | AT50(w), min |  | 27.5 [22.0, 40.1] 32.4 [24.7, 51.0] | 18.4 28.8 | 0.443 (0.027, 0.650) 0.293 (-0.166, 0.540) | 0.319 |  | 0.156 |  | -0.63 | **0.011** | **0.040** | ✗ |  | -0.03 0.31 | 0.912 0.213 |  |
|  | AT50, min |  | 259 [146, 317] 298 [215, 367] | 86.0 117 | 0.266 (-0.276, 0.540) 0.216 (-0.230, 0.494) | 0.346 |  | 0.595 |  | -0.38 | 0.123 |  | — |  | -0.10 0.19 | 0.716 0.481 |  |
|  | Entropy, nats |  | 3.82 [3.77, 3.85] 3.85 [3.84, 3.87] | 0.0483 0.0422 | 0.376 (-0.072, 0.606) 0.406 (0.015, 0.614) | 0.735 |  | 0.462 |  | 0.11 | 0.675 |  | — |  | 0.51 -0.45 | **0.045** **0.042** | 0.838 0.980 |
|  | Max, % |  | 96.5 [95.3, 97.7] 96.6 [95.9, 98.2] | 1.33 1.86 | 0.629 (0.364, 0.766) 0.643 (0.395, 0.772) | 0.981 |  | 0.939 |  | -0.56 | 0.059 |  | — |  | -0.17 0.20 | 0.409 0.279 |  |
|  | Prevalence, % |  | 12.5 [10.0, 15.3] 13.2 [10.5, 17.5] | 3.85 2.95 | 0.594 (0.293, 0.745) 0.588 (0.319, 0.732) | 0.812 |  | 0.738 |  | -0.54 | 0.066 |  | — |  | -0.04 0.18 | 0.850 0.343 |  |
| REM | | | | | | | | | | | | | | | | | |
|  | AT05, min | NT1: NT2: | 116 [17.3, 185] 170 [135, 229] | 102 32.0 | 0.128 (-0.427, 0.448) 0.768 (0.608, 0.852) | **0.001** | **0.028** | 0.428 |  | -0.62 | **0.023** | 0.070 | ✓ |  | -0.05 -0.11 | 0.857 0.492 |  |
|  | AT10, min |  | 141 [30.8, 207] 227 [166, 261] | 107 52.0 | 0.235 (-0.309, 0.516) 0.696 (0.492, 0.804) | **0.003** | **0.046** | 0.00 |  | -0.65 | **0.018** | 0.056 | ✓ |  | 0.14 -0.24 | 0.624 0.142 |  |
|  | AT30, min |  | 325 [276, 490] 405 [302, 464] | 109 97.0 | 0.285 (-0.217, 0.547) 0.194 (-0.328, 0.475) | 0.670 |  | 0.00 |  | -0.04 | 0.888 |  | — |  | 0.05 -0.21 | 0.865 0.437 |  |
|  | AT50(w), min |  | 87.4 [64.9, 124] 88.5 [74.7, 135] | 26.1 24.5 | 0.468 (0.083, 0.664) 0.716 (0.527, 0.816) | 0.066 |  | 0.584 |  | -0.02 | 0.937 |  | — |  | 0.08 0.01 | 0.733 0.973 |  |
|  | AT50, min |  | 577 [487, 666] 605 [525, 644] | 91.5 50.0 | 0.252 (-0.281, 0.527) 0.518 (0.184, 0.691) | 0.110 |  | 0.00 |  | 0.16 | 0.558 |  | — |  | -0.14 -0.24 | 0.612 0.246 |  |
|  | Entropy, nats |  | 3.84 [3.83, 3.88] 3.84 [3.82, 3.86] | 0.0426 0.0219 | 0.375 (-0.068, 0.605) 0.320 (-0.136, 0.563) | 0.521 |  | 0.372 |  | 0.39 | 0.134 |  | — |  | -0.06 -0.23 | 0.833 0.359 |  |
|  | Max, % |  | 99.6 [99.5, 99.8] 99.8 [99.6, 99.9] | 0.152 0.0783 | 0.482 (0.103, 0.674) 0.719 (0.521, 0.821) | **0.032** | 0.157 | 0.675 |  | -0.65 | **0.027** | 0.077 | ✗ |  | -0.11 -0.38 | 0.630 **0.027** | 0.980 |
|  | Prevalence, % |  | 16.6 [14.1, 18.1] 16.9 [12.4, 21.3] | 4.99 4.42 | 0.565 (0.252, 0.726) 0.554 (0.258, 0.711) | 0.446 |  | 0.831 |  | -0.18 | 0.538 |  | — |  | 0.08 0.24 | 0.700 0.243 |  |
| Wake | | | | | | | | | | | | | | | | | |
|  | AT05, min | NT1: NT2: | 134 [60.5, 152] 69.0 [24.0, 147] | 32.5 59.0 | 0.342 (-0.170, 0.591) 0.279 (-0.177, 0.529) | 0.977 |  | 0.00 |  | 0.68 | **0.007** | **0.029** | ✓ |  | -0.21 -0.28 | 0.439 0.263 |  |
|  | AT10, min |  | 178 [162, 237] 155 [59.0, 185] | 56.0 58.0 | 0.384 (-0.054, 0.622) 0.308 (-0.162, 0.556) | 0.459 |  | 0.320 |  | 0.83 | **0.001** | **0.005** | ✓ |  | -0.43 -0.29 | 0.090 0.255 |  |
|  | AT30, min |  | 415 [324, 477] 319 [182, 386] | 129 80.0 | 0.493 (0.128, 0.682) 0.327 (-0.111, 0.562) | 0.324 |  | 0.891 |  | 0.55 | **0.031** | 0.081 | ✗ |  | -0.36 0.18 | 0.126 0.472 |  |
|  | AT50(w), min |  | 79.6 [51.2, 128] 42.4 [27.9, 61.8] | 39.3 16.0 | 0.616 (0.329, 0.759) 0.614 (0.352, 0.753) | 0.741 |  | 0.742 |  | 1.23 | **0.000** | **0.000** | ✓ |  | -0.31 -0.06 | 0.143 0.745 |  |
|  | AT50, min |  | 610 [495, 696] 482 [371, 593] | 154 171 | 0.598 (0.310, 0.747) 0.340 (-0.094, 0.571) | 0.209 |  | 0.604 |  | 0.58 | **0.028** | 0.077 | ✗ |  | -0.41 0.07 | 0.053 0.782 |  |
|  | Entropy, nats |  | 3.99 [3.83, 4.05] 3.95 [3.86, 3.96] | 0.0945 0.0945 | 0.381 (-0.058, 0.608) 0.255 (-0.225, 0.514) | 0.409 |  | 0.463 |  | 0.11 | 0.679 |  | — |  | -0.00 0.01 | 0.994 0.968 |  |
|  | Max, % |  | 99.8 [99.7, 99.9] 99.8 [99.7, 99.9] | 0.0393 0.0941 | 0.709 (0.486, 0.820) 0.251 (-0.240, 0.514) | **0.008** | 0.074 | 0.869 |  | 0.72 | **0.005** | **0.025** | ✗ |  | 0.14 -0.36 | 0.459 0.164 |  |
|  | Prevalence, % |  | 13.9 [8.16, 20.9] 9.04 [8.20, 15.2] | 5.09 3.06 | 0.716 (0.506, 0.822) 0.763 (0.598, 0.848) | 0.892 |  | 0.809 |  | 1.05 | **0.000** | **0.003** | ✓ |  | -0.12 -0.24 | 0.503 0.104 |  |
| N1•N2 | | | | | | | | | | | | | | | | | |
|  | AT05, min | NT1: NT2: | 50.5 [25.8, 64.0] 21.0 [15.0, 47.0] | 30.0 9.00 | 0.525 (0.178, 0.702) 0.467 (0.106, 0.658) | 0.848 |  | 0.0225 |  | 0.76 | **0.006** | **0.027** | ✓ |  | -0.27 -0.14 | 0.232 0.537 |  |
|  | AT10, min |  | 118 [103, 158] 89.0 [62.0, 134] | 20.0 37.0 | 0.729 (0.536, 0.829) 0.453 (0.099, 0.644) | 0.084 |  | 0.00 |  | 0.74 | **0.007** | **0.029** | ✓ |  | -0.07 -0.31 | 0.684 0.164 |  |
|  | AT30, min |  | 327 [307, 378] 310 [278, 335] | 51.5 91.0 | 0.553 (0.236, 0.717) 0.088 (-0.482, 0.405) | **0.015** | 0.106 | 0.429 |  | 0.63 | **0.011** | **0.041** | ✓ |  | -0.14 -0.19 | 0.516 0.495 |  |
|  | AT50(w), min |  | 51.5 [40.0, 60.5] 37.5 [31.3, 49.4] | 8.19 9.12 | 0.721 (0.511, 0.826) 0.771 (0.616, 0.853) | 0.824 |  | 0.310 |  | 0.97 | **0.002** | **0.011** | ✓ |  | 0.11 -0.12 | 0.540 0.439 |  |
|  | AT50, min |  | 528 [495, 563] 476 [417, 516] | 53.0 80.0 | 0.471 (0.093, 0.666) 0.151 (-0.265, 0.431) | 0.142 |  | 0.246 |  | 0.69 | **0.004** | **0.023** | ✓ |  | 0.01 -0.05 | 0.982 0.850 |  |
|  | Entropy, nats |  | 3.99 [3.96, 4.00] 4.00 [3.96, 4.01] | 0.0251 0.0393 | 0.253 (-0.249, 0.528) 0.092 (-0.498, 0.409) | 0.289 |  | 0.275 |  | -0.20 | 0.402 |  | — |  | -0.22 -0.25 | 0.435 0.371 |  |
|  | Max, % |  | 48.9 [48.2, 49.2] 48.4 [47.8, 48.7] | 0.398 0.755 | 0.316 (-0.165, 0.566) 0.124 (-0.425, 0.432) | 0.155 |  | 0.00 |  | 0.41 | 0.084 |  | — |  | -0.00 0.18 | 0.988 0.530 |  |
|  | Prevalence, % |  | 9.63 [7.76, 10.5] 8.01 [6.82, 10.3] | 1.25 1.19 | 0.744 (0.556, 0.840) 0.731 (0.547, 0.827) | 0.988 |  | 0.666 |  | 0.69 | **0.028** | 0.077 | ✗ |  | 0.17 -0.19 | 0.337 0.246 |  |
| N1•N3 | | | | | | | | | | | | | | | | | |
|  | AT05, min | NT1: NT2: | 50.0 [37.3, 63.8] 41.0 [35.0, 52.0] | 12.5 10.0 | 0.429 (-0.000, 0.643) 0.617 (0.358, 0.753) | 0.269 |  | 0.00 |  | 0.33 | 0.262 |  | — |  | -0.32 -0.13 | 0.206 0.501 |  |
|  | AT10, min |  | 80.5 [75.0, 100] 81.0 [73.0, 92.0] | 16.5 20.0 | 0.535 (0.190, 0.709) 0.503 (0.180, 0.676) | 0.520 |  | 0.00974 |  | 0.19 | 0.516 |  | — |  | -0.14 -0.19 | 0.543 0.375 |  |
|  | AT30, min |  | 231 [194, 272] 259 [212, 285] | 16.5 39.0 | 0.620 (0.336, 0.763) 0.475 (0.123, 0.660) | 0.690 |  | 0.592 |  | -0.16 | 0.584 |  | — |  | 0.10 -0.11 | 0.634 0.624 |  |
|  | AT50(w), min |  | 5.16 [4.51, 5.52] 5.14 [4.03, 6.91] | 1.03 1.20 | 0.729 (0.534, 0.829) 0.601 (0.334, 0.742) | 0.286 |  | 0.821 |  | -0.01 | 0.986 |  | — |  | 0.21 -0.08 | 0.217 0.675 |  |
|  | AT50, min |  | 413 [365, 448] 428 [383, 444] | 26.0 44.0 | 0.516 (0.167, 0.694) 0.268 (-0.211, 0.524) | 0.130 |  | 0.905 |  | 0.28 | 0.307 |  | — |  | 0.26 -0.13 | 0.254 0.615 |  |
|  | Entropy, nats |  | 3.87 [3.82, 3.89] 3.84 [3.82, 3.91] | 0.0742 0.0292 | 0.190 (-0.383, 0.487) 0.584 (0.306, 0.731) | **0.010** | 0.082 | 0.362 |  | -0.09 | 0.720 |  | — |  | 0.40 0.22 | 0.176 0.267 |  |
|  | Max, % |  | 7.55 [6.49, 11.7] 8.23 [6.38, 12.6] | 2.89 1.64 | 0.424 (0.004, 0.638) 0.434 (0.067, 0.631) | 0.905 |  | 0.739 |  | -0.26 | 0.339 |  | — |  | -0.11 -0.04 | 0.668 0.857 |  |
|  | Prevalence, % |  | 1.21 [1.10, 1.43] 1.15 [1.06, 1.73] | 0.150 0.136 | 0.768 (0.600, 0.853) 0.649 (0.414, 0.773) | 0.250 |  | 0.874 |  | -0.14 | 0.650 |  | — |  | 0.09 0.05 | 0.587 0.777 |  |
| N1•REM | | | | | | | | | | | | | | | | | |
|  | AT05, min | NT1: NT2: | 60.5 [13.8, 103] 131 [43.0, 164] | 49.5 18.0 | 0.294 (-0.218, 0.555) 0.533 (0.231, 0.695) | 0.363 |  | 0.164 |  | -0.61 | **0.022** | 0.067 | ✗ |  | 0.03 -0.11 | 0.914 0.607 |  |
|  | AT10, min |  | 138 [40.5, 199] 175 [140, 232] | 119 26.0 | 0.219 (-0.335, 0.505) 0.663 (0.432, 0.784) | **0.007** | 0.069 | 0.00 |  | -0.38 | 0.172 |  | — |  | -0.13 -0.19 | 0.663 0.298 |  |
|  | AT30, min |  | 386 [305, 474] 350 [296, 401] | 84.5 77.0 | 0.119 (-0.499, 0.441) 0.180 (-0.272, 0.472) | 0.921 |  | 0.00 |  | 0.19 | 0.419 |  | — |  | -0.25 0.10 | 0.408 0.718 |  |
|  | AT50(w), min |  | 24.2 [21.5, 36.2] 19.1 [17.2, 24.0] | 4.09 4.08 | 0.652 (0.403, 0.780) 0.696 (0.500, 0.802) | 0.508 |  | 0.646 |  | 1.10 | **0.000** | **0.002** | ✓ |  | 0.07 -0.06 | 0.707 0.696 |  |
|  | AT50, min |  | 626 [573, 662] 584 [544, 602] | 66.0 73.0 | 0.449 (0.056, 0.652) -0.172 (-0.978, 0.225) | **0.005** | 0.053 | 0.350 |  | 0.49 | **0.027** | 0.077 | ✗ |  | -0.56 -0.25 | **0.016** 0.403 | 0.838 |
|  | Entropy, nats |  | 3.86 [3.83, 3.89] 3.87 [3.84, 3.90] | 0.0300 0.0343 | 0.479 (0.070, 0.679) 0.173 (-0.360, 0.461) | 0.134 |  | 0.457 |  | -0.08 | 0.766 |  | — |  | 0.02 -0.47 | 0.934 0.082 |  |
|  | Max, % |  | 44.2 [39.9, 45.9] 41.6 [38.7, 45.5] | 1.19 4.17 | 0.640 (0.385, 0.772) 0.424 (0.035, 0.628) | 0.133 |  | 0.669 |  | 0.53 | 0.065 |  | — |  | -0.05 -0.03 | 0.784 0.897 |  |
|  | Prevalence, % |  | 4.30 [3.64, 5.56] 3.70 [3.17, 4.64] | 0.856 0.367 | 0.688 (0.460, 0.804) 0.838 (0.728, 0.896) | **0.045** | 0.201 | 0.767 |  | 1.13 | **0.000** | **0.002** | ✓ |  | 0.21 -0.12 | 0.278 0.323 |  |
| N2•N3 | | | | | | | | | | | | | | | | | |
|  | AT05, min | NT1: NT2: | 47.0 [33.3, 61.3] 45.0 [34.0, 70.0] | 23.0 14.0 | 0.448 (-0.012, 0.659) 0.722 (0.525, 0.823) | 0.053 |  | 0.00 |  | -0.02 | 0.949 |  | — |  | 0.08 0.22 | 0.753 0.195 |  |
|  | AT10, min |  | 64.0 [51.3, 81.5] 67.0 [52.0, 94.0] | 18.0 21.0 | 0.705 (0.469, 0.818) 0.514 (0.173, 0.690) | 0.454 |  | 0.00 |  | -0.32 | 0.312 |  | — |  | 0.06 -0.07 | 0.751 0.753 |  |
|  | AT30, min |  | 179 [141, 231] 211 [159, 291] | 42.5 53.0 | 0.198 (-0.351, 0.501) 0.370 (-0.070, 0.597) | 0.912 |  | 0.592 |  | -0.50 | 0.053 |  | — |  | 0.02 0.12 | 0.945 0.616 |  |
|  | AT50(w), min |  | 45.9 [38.5, 51.8] 47.7 [38.1, 60.6] | 9.45 13.9 | 0.593 (0.296, 0.743) 0.482 (0.132, 0.666) | 0.623 |  | 0.636 |  | -0.74 | **0.008** | **0.034** | ✓ |  | -0.14 0.19 | 0.502 0.395 |  |
|  | AT50, min |  | 349 [311, 398] 359 [292, 419] | 52.5 76.0 | 0.225 (-0.374, 0.517) 0.338 (-0.141, 0.579) | 0.957 |  | 0.347 |  | -0.05 | 0.845 |  | — |  | -0.15 0.15 | 0.612 0.551 |  |
|  | Entropy, nats |  | 3.81 [3.79, 3.82] 3.83 [3.79, 3.85] | 0.0415 0.0317 | 0.476 (0.099, 0.669) 0.454 (0.088, 0.647) | 0.602 |  | 0.249 |  | 0.21 | 0.448 |  | — |  | 0.47 -0.23 | **0.044** 0.314 | 0.838 |
|  | Max, % |  | 49.9 [49.8, 49.9] 49.8 [49.8, 49.9] | 0.0730 0.0645 | 0.309 (-0.188, 0.564) 0.201 (-0.311, 0.479) | 0.594 |  | 0.697 |  | -0.19 | 0.448 |  | — |  | -0.01 0.10 | 0.981 0.721 |  |
|  | Prevalence, % |  | 12.5 [10.7, 14.4] 13.1 [12.8, 15.1] | 2.37 2.01 | 0.631 (0.358, 0.768) 0.475 (0.135, 0.658) | 0.221 |  | 0.709 |  | -0.83 | **0.003** | **0.017** | ✓ |  | -0.04 0.29 | 0.839 0.175 |  |
| N2•REM | | | | | | | | | | | | | | | | | |
|  | AT05, min | NT1: NT2: | 38.0 [23.0, 105] 117 [63.0, 124] | 29.5 12.0 | 0.180 (-0.410, 0.483) 0.698 (0.499, 0.804) | **0.008** | 0.073 | 0.0501 |  | -0.67 | **0.014** | **0.045** | ✗ |  | -0.02 -0.08 | 0.957 0.646 |  |
|  | AT10, min |  | 81.5 [36.8, 157] 144 [126, 179] | 81.0 21.0 | 0.249 (-0.314, 0.530) 0.761 (0.599, 0.846) | **0.004** | **0.046** | 0.00 |  | -0.50 | 0.085 |  | — |  | 0.04 -0.00 | 0.884 0.990 |  |
|  | AT30, min |  | 340 [298, 406] 310 [277, 348] | 55.0 48.0 | 0.234 (-0.304, 0.515) 0.373 (-0.027, 0.591) | 0.583 |  | 0.0645 |  | 0.22 | 0.377 |  | — |  | -0.21 0.34 | 0.465 0.151 |  |
|  | AT50(w), min |  | 38.5 [35.6, 43.3] 33.9 [33.2, 37.5] | 6.65 7.42 | 0.579 (0.279, 0.733) 0.623 (0.370, 0.757) | 0.499 |  | 0.818 |  | -0.01 | 0.984 |  | — |  | 0.02 0.26 | 0.924 0.161 |  |
|  | AT50, min |  | 568 [506, 618] 504 [475, 539] | 56.5 63.0 | 0.367 (-0.101, 0.605) 0.228 (-0.241, 0.498) | 0.409 |  | 0.204 |  | 0.62 | **0.013** | **0.043** | ✗ |  | -0.43 0.28 | 0.105 0.285 |  |
|  | Entropy, nats |  | 3.89 [3.88, 3.95] 3.92 [3.89, 3.95] | 0.0285 0.0394 | 0.550 (0.226, 0.716) 0.487 (0.139, 0.669) | 0.591 |  | 0.560 |  | 0.30 | 0.287 |  | — |  | 0.22 -0.30 | 0.313 0.179 |  |
|  | Max, % |  | 47.8 [44.9, 48.4] 48.3 [47.7, 48.5] | 2.44 0.546 | -0.203 (-0.894, 0.236) 0.820 (0.686, 0.886) | **0.000** | **0.000** | 0.253 |  | -0.78 | **0.001** | **0.008** | ✓ |  | -0.29 -0.05 | 0.413 0.702 |  |
|  | Prevalence, % |  | 6.92 [6.38, 7.25] 7.02 [6.23, 7.91] | 0.804 0.688 | 0.731 (0.539, 0.830) 0.611 (0.349, 0.748) | 0.209 |  | 0.753 |  | -0.35 | 0.257 |  | — |  | 0.26 0.11 | 0.118 0.557 |  |
| N3•REM | | | | | | | | | | | | | | | | | |
|  | AT05, min | NT1: NT2: | 36.0 [26.3, 47.8] 68.0 [51.0, 94.0] | 19.5 20.0 | 0.585 (0.332, 0.736) 0.744 (0.575, 0.834) | 0.222 |  | 0.00 |  | -1.03 | **0.000** | **0.003** | ✓ |  | 0.17 -0.07 | 0.426 0.659 |  |
|  | AT10, min |  | 73.5 [54.0, 111] 122 [87.0, 139] | 22.0 31.0 | 0.503 (0.140, 0.688) 0.802 (0.671, 0.872) | **0.020** | 0.123 | 0.0347 |  | -0.85 | **0.003** | **0.019** | ✓ |  | 0.27 -0.15 | 0.248 0.261 |  |
|  | AT30, min |  | 273 [232, 316] 300 [256, 333] | 46.5 59.0 | 0.395 (-0.050, 0.620) 0.422 (0.049, 0.624) | 0.870 |  | 0.00 |  | -0.26 | 0.336 |  | — |  | 0.28 0.06 | 0.277 0.794 |  |
|  | AT50(w), min |  | 5.96 [4.42, 7.01] 6.32 [5.15, 6.99] | 1.15 1.78 | 0.733 (0.537, 0.832) 0.463 (0.096, 0.654) | **0.049** | 0.210 | 0.761 |  | -0.51 | 0.087 |  | — |  | 0.09 0.05 | 0.599 0.814 |  |
|  | AT50, min |  | 477 [413, 560] 463 [420, 488] | 70.5 48.0 | 0.354 (-0.118, 0.597) 0.428 (0.051, 0.629) | 0.636 |  | 0.0623 |  | 0.48 | 0.078 |  | — |  | 0.01 0.06 | 0.979 0.810 |  |
|  | Entropy, nats |  | 3.92 [3.88, 3.96] 3.93 [3.90, 3.98] | 0.0485 0.0234 | 0.457 (0.059, 0.659) 0.323 (-0.121, 0.561) | 0.443 |  | 0.670 |  | -0.43 | 0.104 |  | — |  | -0.18 -0.00 | 0.461 0.987 |  |
|  | Max, % |  | 8.43 [7.61, 9.74] 12.2 [10.6, 14.0] | 1.89 2.86 | 0.538 (0.299, 0.709) 0.382 (-0.052, 0.606) | 0.412 |  | 0.346 |  | -0.91 | **0.001** | **0.005** | ✓ |  | -0.02 -0.09 | 0.937 0.725 |  |
|  | Prevalence, % |  | 1.25 [1.05, 1.46] 1.29 [1.10, 1.53] | 0.269 0.315 | 0.679 (0.449, 0.797) 0.531 (0.212, 0.698) | 0.318 |  | 0.880 |  | -0.69 | **0.019** | 0.059 | ✓ |  | -0.07 0.11 | 0.706 0.597 |  |
| Wake•N1 | | | | | | | | | | | | | | | | | |
|  | AT05, min | NT1: NT2: | 91.5 [10.8, 111] 14.0 [8.00, 40.0] | 50.5 14.0 | 0.115 (-0.503, 0.440) 0.697 (0.482, 0.807) | **0.000** | **0.014** | 0.103 |  | 0.93 | **0.000** | **0.003** | ✓ |  | 0.02 -0.23 | 0.952 0.180 |  |
|  | AT10, min |  | 148 [106, 219] 79.0 [52.0, 155] | 61.5 53.0 | -0.055 (-0.907, 0.331) 0.419 (0.033, 0.625) | 0.105 |  | 0.0856 |  | 0.86 | **0.000** | **0.002** | ✓ |  | -0.06 -0.22 | 0.854 0.355 |  |
|  | AT30, min |  | 357 [303, 443] 315 [281, 364] | 77.5 83.0 | 0.693 (0.466, 0.807) 0.308 (-0.140, 0.549) | **0.006** | 0.062 | 0.625 |  | 0.87 | **0.001** | **0.006** | ✓ |  | -0.09 -0.30 | 0.617 0.220 |  |
|  | AT50(w), min |  | 23.5 [18.8, 38.7] 16.8 [10.3, 25.0] | 5.13 4.28 | 0.730 (0.521, 0.833) 0.873 (0.782, 0.920) | **0.013** | 0.100 | 0.693 |  | 1.35 | **0.000** | **0.000** | ✓ |  | 0.24 -0.08 | 0.191 0.469 |  |
|  | AT50, min |  | 575 [462, 676] 517 [381, 604] | 78.5 71.0 | 0.672 (0.436, 0.794) 0.553 (0.255, 0.711) | 0.517 |  | 0.624 |  | 0.92 | **0.001** | **0.005** | ✓ |  | -0.08 -0.16 | 0.679 0.447 |  |
|  | Entropy, nats |  | 3.98 [3.96, 4.01] 4.00 [3.96, 4.01] | 0.0344 0.0821 | 0.167 (-0.506, 0.472) -0.019 (-0.706, 0.344) | 0.651 |  | 0.00 |  | -0.03 | 0.884 |  | — |  | 0.03 -0.05 | 0.923 0.870 |  |
|  | Max, % |  | 46.5 [46.4, 47.7] 46.7 [45.9, 46.9] | 0.782 0.696 | 0.040 (-0.504, 0.403) 0.263 (-0.233, 0.525) | 0.794 |  | 0.925 |  | 0.28 | 0.232 |  | — |  | 0.10 0.06 | 0.769 0.817 |  |
|  | Prevalence, % |  | 4.79 [4.07, 6.76] 4.14 [2.58, 5.75] | 0.553 0.894 | 0.828 (0.701, 0.892) 0.745 (0.564, 0.837) | 0.194 |  | 0.939 |  | 1.14 | **0.000** | **0.002** | ✓ |  | 0.17 -0.25 | 0.239 0.107 |  |
| Wake•N2 | | | | | | | | | | | | | | | | | |
|  | AT05, min | NT1: NT2: | 60.5 [37.0, 87.5] 28.0 [22.0, 53.0] | 19.0 12.0 | 0.280 (-0.230, 0.544) 0.550 (0.247, 0.711) | 0.126 |  | 0.282 |  | 0.91 | **0.000** | **0.003** | ✓ |  | 0.07 -0.16 | 0.806 0.443 |  |
|  | AT10, min |  | 138 [107, 163] 95.0 [61.0, 122] | 24.5 30.0 | 0.668 (0.422, 0.793) 0.452 (0.083, 0.647) | 0.115 |  | 0.00 |  | 1.02 | **0.000** | **0.002** | ✗ |  | -0.39 -0.19 | **0.043** 0.400 | 0.838 |
|  | AT30, min |  | 317 [255, 367] 296 [253, 348] | 52.5 82.0 | 0.559 (0.242, 0.723) 0.257 (-0.221, 0.516) | **0.022** | 0.123 | 0.170 |  | 0.67 | **0.009** | **0.036** | ✗ |  | -0.08 -0.34 | 0.715 0.181 |  |
|  | AT50(w), min |  | 37.7 [29.6, 41.6] 28.7 [23.3, 49.4] | 5.18 6.95 | 0.750 (0.550, 0.847) 0.740 (0.557, 0.835) | 0.734 |  | 0.721 |  | 0.89 | **0.006** | **0.026** | ✓ |  | 0.38 0.11 | **0.032** 0.522 | 0.838 |
|  | AT50, min |  | 496 [444, 559] 476 [411, 505] | 36.5 43.0 | 0.623 (0.352, 0.763) 0.601 (0.331, 0.743) | 0.764 |  | 0.359 |  | 0.77 | **0.007** | **0.029** | ✗ |  | 0.02 -0.05 | 0.929 0.794 |  |
|  | Entropy, nats |  | 3.99 [3.96, 4.01] 3.96 [3.91, 3.99] | 0.0333 0.0253 | 0.425 (0.015, 0.636) 0.201 (-0.317, 0.479) | 0.139 |  | 0.447 |  | 0.45 | 0.076 |  | — |  | -0.08 -0.12 | 0.744 0.652 |  |
|  | Max, % |  | 47.0 [46.6, 47.9] 46.7 [45.9, 47.6] | 0.785 0.927 | -0.045 (-0.593, 0.309) 0.305 (-0.149, 0.549) | 0.361 |  | 0.453 |  | 0.02 | 0.939 |  | — |  | -0.35 0.37 | 0.288 0.140 |  |
|  | Prevalence, % |  | 7.39 [6.01, 8.09] 7.08 [5.38, 9.32] | 0.509 0.896 | 0.659 (0.392, 0.790) 0.646 (0.397, 0.774) | 0.747 |  | 0.951 |  | 0.54 | 0.090 |  | — |  | 0.40 -0.01 | **0.048** 0.967 | 0.838 |
| Wake•N3 | | | | | | | | | | | | | | | | | |
|  | AT05, min | NT1: NT2: | 56.5 [41.3, 70.5] 51.0 [37.0, 61.0] | 13.5 12.0 | 0.256 (-0.331, 0.538) 0.453 (0.101, 0.643) | 0.525 |  | 0.00 |  | 0.41 | 0.127 |  | — |  | -0.12 -0.26 | 0.673 0.239 |  |
|  | AT10, min |  | 81.5 [71.0, 91.8] 80.0 [70.0, 100] | 27.0 23.0 | 0.199 (-0.398, 0.496) 0.468 (0.117, 0.655) | 0.528 |  | 0.00 |  | 0.03 | 0.920 |  | — |  | -0.21 -0.32 | 0.486 0.136 |  |
|  | AT30, min |  | 180 [172, 251] 225 [175, 308] | 42.0 75.0 | 0.466 (0.087, 0.662) 0.110 (-0.477, 0.422) | 0.304 |  | 0.731 |  | -0.39 | 0.122 |  | — |  | 0.13 -0.05 | 0.579 0.864 |  |
|  | AT50(w), min |  | 7.04 [4.62, 7.99] 6.50 [5.04, 8.65] | 0.380 1.63 | 0.812 (0.673, 0.882) 0.587 (0.314, 0.733) | 0.071 |  | 0.711 |  | -0.24 | 0.427 |  | — |  | 0.23 0.06 | 0.124 0.781 |  |
|  | AT50, min |  | 386 [338, 413] 351 [306, 428] | 41.5 65.0 | 0.487 (0.124, 0.676) 0.204 (-0.318, 0.482) | 0.102 |  | 0.731 |  | 0.15 | 0.582 |  | — |  | 0.03 0.05 | 0.890 0.846 |  |
|  | Entropy, nats |  | 3.83 [3.80, 3.85] 3.84 [3.80, 3.86] | 0.0576 0.0210 | 0.288 (-0.218, 0.550) 0.523 (0.203, 0.692) | 0.120 |  | 0.0598 |  | 0.07 | 0.812 |  | — |  | 0.77 0.25 | **0.003** 0.239 | 0.831 |
|  | Max, % |  | 24.1 [15.4, 31.3] 24.1 [15.5, 31.4] | 5.36 6.58 | 0.598 (0.302, 0.748) 0.558 (0.274, 0.712) | 0.493 |  | 0.396 |  | -0.22 | 0.442 |  | — |  | -0.01 0.29 | 0.966 0.140 |  |
|  | Prevalence, % |  | 1.80 [1.42, 2.13] 1.68 [1.48, 2.32] | 0.237 0.221 | 0.770 (0.601, 0.856) 0.789 (0.653, 0.863) | 0.701 |  | 0.701 |  | -0.28 | 0.381 |  | — |  | 0.18 0.21 | 0.263 0.123 |  |
| Wake•REM | | | | | | | | | | | | | | | | | |
|  | AT05, min | NT1: NT2: | 25.0 [11.5, 160] 127 [40.0, 156] | 98.5 17.0 | -0.047 (-0.850, 0.327) 0.547 (0.253, 0.705) | **0.003** | **0.045** | 0.116 |  | -0.32 | 0.211 |  | — |  | 0.10 -0.16 | 0.758 0.429 |  |
|  | AT10, min |  | 138 [27.8, 216] 174 [113, 186] | 114 53.0 | -0.147 (-0.735, 0.223) 0.567 (0.280, 0.720) | **0.003** | **0.045** | 0.00 |  | -0.06 | 0.796 |  | — |  | 0.13 -0.09 | 0.705 0.647 |  |
|  | AT30, min |  | 373 [305, 472] 357 [326, 397] | 90.5 104 | 0.184 (-0.388, 0.482) 0.107 (-0.383, 0.419) | 0.593 |  | 0.00 |  | 0.30 | 0.178 |  | — |  | 0.03 -0.12 | 0.906 0.659 |  |
|  | AT50(w), min |  | 23.8 [16.1, 29.3] 16.1 [12.1, 21.2] | 2.83 3.61 | 0.840 (0.722, 0.899) 0.834 (0.721, 0.893) | 0.830 |  | 0.843 |  | 1.19 | **0.000** | **0.002** | ✓ |  | 0.28 -0.06 | **0.037** 0.643 | 0.838 |
|  | AT50, min |  | 565 [514, 587] 580 [503, 616] | 66.0 79.0 | 0.499 (0.137, 0.684) 0.072 (-0.418, 0.398) | 0.094 |  | 0.165 |  | 0.25 | 0.310 |  | — |  | 0.09 -0.23 | 0.692 0.430 |  |
|  | Entropy, nats |  | 3.85 [3.84, 3.87] 3.86 [3.84, 3.89] | 0.0288 0.0440 | 0.495 (0.121, 0.685) 0.152 (-0.390, 0.446) | **0.041** | 0.190 | 0.598 |  | -0.32 | 0.209 |  | — |  | -0.17 -0.30 | 0.470 0.266 |  |
|  | Max, % |  | 45.7 [43.6, 46.2] 42.8 [41.5, 45.9] | 1.50 2.02 | 0.770 (0.589, 0.858) 0.607 (0.354, 0.744) | 0.053 |  | 0.491 |  | 0.78 | **0.009** | **0.034** | ✓ |  | 0.23 0.32 | 0.157 0.084 |  |
|  | Prevalence, % |  | 4.15 [3.05, 5.19] 3.04 [2.78, 4.17] | 0.581 0.318 | 0.863 (0.763, 0.914) 0.811 (0.686, 0.877) | 0.882 |  | 0.768 |  | 1.03 | **0.001** | **0.005** | ✓ |  | 0.23 -0.07 | 0.061 0.613 |  |
| N1•N2•N3 | | | | | | | | | | | | | | | | | |
|  | AT05, min | NT1: NT2: | 54.5 [36.0, 61.5] 39.0 [33.0, 51.0] | 18.5 8.00 | 0.340 (-0.153, 0.587) 0.631 (0.384, 0.763) | 0.204 |  | 0.00 |  | 0.32 | 0.257 |  | — |  | -0.25 -0.04 | 0.359 0.851 |  |
|  | AT10, min |  | 90.5 [83.8, 112] 85.0 [80.0, 89.0] | 15.5 25.0 | 0.542 (0.203, 0.713) 0.448 (0.063, 0.648) | 0.351 |  | 0.00 |  | 0.36 | 0.212 |  | — |  | -0.14 0.06 | 0.539 0.794 |  |
|  | AT30, min |  | 256 [225, 276] 264 [251, 304] | 35.5 53.0 | 0.500 (0.118, 0.688) 0.400 (0.002, 0.612) | 0.720 |  | 0.649 |  | -0.20 | 0.486 |  | — |  | -0.01 -0.21 | 0.957 0.366 |  |
|  | AT50(w), min |  | 16.3 [14.4, 18.1] 16.1 [14.3, 21.5] | 1.82 2.77 | 0.683 (0.456, 0.799) 0.548 (0.244, 0.708) | 0.540 |  | 0.804 |  | -0.17 | 0.568 |  | — |  | 0.15 0.00 | 0.424 0.987 |  |
|  | AT50, min |  | 426 [403, 485] 445 [394, 460] | 41.5 37.0 | 0.303 (-0.156, 0.564) 0.348 (-0.087, 0.578) | 0.663 |  | 0.299 |  | 0.40 | 0.132 |  | — |  | 0.28 -0.05 | 0.317 0.843 |  |
|  | Entropy, nats |  | 3.83 [3.81, 3.84] 3.87 [3.82, 3.89] | 0.0573 0.0277 | 0.063 (-0.520, 0.409) 0.445 (0.081, 0.640) | 0.105 |  | 0.652 |  | -0.42 | 0.092 |  | — |  | 0.41 -0.11 | 0.188 0.628 |  |
|  | Max, % |  | 15.2 [13.2, 20.5] 15.8 [13.2, 19.7] | 3.72 2.15 | 0.275 (-0.237, 0.541) 0.411 (0.031, 0.616) | 0.520 |  | 0.722 |  | -0.17 | 0.502 |  | — |  | -0.28 -0.09 | 0.309 0.708 |  |
|  | Prevalence, % |  | 3.65 [3.52, 4.09] 3.62 [3.37, 4.78] | 0.365 0.427 | 0.721 (0.521, 0.823) 0.523 (0.205, 0.692) | 0.334 |  | 0.873 |  | -0.35 | 0.252 |  | — |  | 0.07 0.05 | 0.708 0.810 |  |
| N1•N2•REM | | | | | | | | | | | | | | | | | |
|  | AT05, min | NT1: NT2: | 26.0 [11.3, 81.8] 77.0 [33.0, 114] | 18.5 21.0 | -0.146 (-0.797, 0.277) 0.574 (0.300, 0.722) | **0.003** | **0.045** | 0.0817 |  | -0.23 | 0.364 |  | — |  | 0.27 -0.21 | 0.436 0.286 |  |
|  | AT10, min |  | 118 [62.0, 158] 135 [112, 164] | 43.0 21.0 | 0.184 (-0.396, 0.483) 0.487 (0.158, 0.665) | 0.198 |  | 0.253 |  | -0.09 | 0.742 |  | — |  | -0.05 -0.28 | 0.880 0.189 |  |
|  | AT30, min |  | 352 [298, 433] 319 [297, 357] | 49.5 62.0 | 0.388 (-0.048, 0.613) 0.286 (-0.164, 0.535) | 0.369 |  | 0.556 |  | 0.46 | 0.069 |  | — |  | -0.18 0.01 | 0.478 0.954 |  |
|  | AT50(w), min |  | 28.1 [23.7, 37.7] 20.4 [18.1, 25.9] | 2.45 3.48 | 0.699 (0.483, 0.810) 0.750 (0.582, 0.838) | 0.705 |  | 0.770 |  | 0.78 | **0.011** | **0.039** | ✓ |  | 0.15 0.04 | 0.421 0.794 |  |
|  | AT50, min |  | 588 [539, 646] 500 [469, 548] | 66.0 54.0 | 0.665 (0.406, 0.793) 0.208 (-0.336, 0.476) | **0.006** | 0.062 | 0.836 |  | 0.96 | **0.000** | **0.002** | ✓ |  | -0.40 0.15 | **0.038** 0.572 | 0.838 |
|  | Entropy, nats |  | 3.92 [3.90, 3.94] 3.94 [3.89, 3.97] | 0.0315 0.0461 | 0.540 (0.207, 0.710) 0.279 (-0.197, 0.531) | 0.073 |  | 0.765 |  | 0.33 | 0.208 |  | — |  | 0.04 -0.36 | 0.873 0.158 |  |
|  | Max, % |  | 32.3 [31.8, 32.7] 30.7 [29.9, 32.0] | 0.836 1.15 | 0.667 (0.424, 0.791) 0.170 (-0.374, 0.461) | **0.003** | **0.046** | 0.914 |  | 0.73 | **0.005** | **0.024** | ✓ |  | -0.34 0.17 | 0.071 0.528 |  |
|  | Prevalence, % |  | 5.18 [4.15, 5.49] 4.44 [3.70, 4.96] | 0.549 0.535 | 0.788 (0.632, 0.867) 0.763 (0.606, 0.847) | 0.798 |  | 0.838 |  | 0.59 | 0.067 |  | — |  | 0.23 -0.05 | 0.147 0.760 |  |
| N1•N3•REM | | | | | | | | | | | | | | | | | |
|  | AT05, min | NT1: NT2: | 24.0 [17.5, 79.3] 76.0 [38.0, 96.0] | 30.0 14.0 | 0.014 (-0.560, 0.380) 0.694 (0.491, 0.802) | **0.002** | **0.044** | 0.0913 |  | -0.59 | **0.026** | 0.075 | ✗ |  | 0.20 -0.13 | 0.540 0.449 |  |
|  | AT10, min |  | 96.0 [58.3, 146] 133 [105, 157] | 62.5 19.0 | 0.087 (-0.555, 0.422) 0.667 (0.444, 0.785) | **0.004** | **0.048** | 0.00 |  | -0.40 | 0.143 |  | — |  | 0.09 -0.22 | 0.771 0.216 |  |
|  | AT30, min |  | 310 [273, 367] 307 [266, 343] | 52.5 60.0 | 0.232 (-0.311, 0.514) 0.362 (-0.057, 0.585) | 0.574 |  | 0.438 |  | 0.29 | 0.254 |  | — |  | 0.08 0.12 | 0.786 0.626 |  |
|  | AT50(w), min |  | 6.77 [5.45, 7.52] 5.34 [4.71, 6.32] | 0.768 1.20 | 0.750 (0.570, 0.842) 0.613 (0.351, 0.750) | 0.646 |  | 0.863 |  | 0.39 | 0.218 |  | — |  | 0.16 0.02 | 0.335 0.934 |  |
|  | AT50, min |  | 537 [489, 579] 488 [433, 514] | 41.5 44.0 | 0.533 (0.182, 0.709) 0.110 (-0.501, 0.412) | 0.080 |  | 0.714 |  | 0.79 | **0.001** | **0.008** | ✓ |  | -0.19 0.01 | 0.423 0.972 |  |
|  | Entropy, nats |  | 3.90 [3.87, 3.93] 3.92 [3.88, 3.96] | 0.0258 0.0336 | 0.480 (0.103, 0.672) 0.134 (-0.430, 0.437) | **0.022** | 0.123 | 0.722 |  | -0.16 | 0.507 |  | — |  | -0.06 -0.27 | 0.793 0.338 |  |
|  | Max, % |  | 6.53 [6.04, 7.01] 6.94 [6.30, 8.08] | 0.650 1.85 | 0.712 (0.505, 0.818) 0.306 (-0.153, 0.551) | **0.001** | **0.021** | 0.618 |  | -0.50 | 0.076 |  | — |  | 0.15 -0.04 | 0.393 0.865 |  |
|  | Prevalence, % |  | 1.26 [1.09, 1.40] 1.12 [0.997, 1.42] | 0.183 0.146 | 0.779 (0.620, 0.861) 0.646 (0.409, 0.771) | 0.395 |  | 0.797 |  | 0.15 | 0.645 |  | — |  | 0.27 0.03 | 0.077 0.884 |  |
| N2•N3•REM | | | | | | | | | | | | | | | | | |
|  | AT05, min | NT1: NT2: | 37.0 [31.3, 58.8] 51.0 [42.0, 66.0] | 16.5 20.0 | 0.549 (0.214, 0.717) 0.561 (0.267, 0.717) | 0.782 |  | 0.00 |  | -0.66 | **0.022** | 0.068 | ✗ |  | 0.11 -0.05 | 0.637 0.824 |  |
|  | AT10, min |  | 83.0 [65.8, 111] 110 [80.0, 123] | 16.0 29.0 | 0.320 (-0.156, 0.570) 0.471 (0.115, 0.659) | 0.654 |  | 0.00 |  | -0.57 | **0.033** | 0.082 | ✓ |  | 0.07 0.09 | 0.805 0.682 |  |
|  | AT30, min |  | 262 [229, 310] 278 [253, 310] | 48.0 48.0 | 0.487 (0.107, 0.678) 0.451 (0.096, 0.642) | 0.904 |  | 0.627 |  | -0.34 | 0.233 |  | — |  | -0.04 0.09 | 0.867 0.703 |  |
|  | AT50(w), min |  | 14.7 [11.4, 16.7] 15.7 [13.1, 17.2] | 1.83 2.95 | 0.757 (0.579, 0.847) 0.566 (0.272, 0.719) | 0.219 |  | 0.686 |  | -0.66 | **0.031** | 0.081 | ✓ |  | 0.05 0.15 | 0.777 0.451 |  |
|  | AT50, min |  | 454 [411, 513] 452 [407, 473] | 48.0 48.0 | 0.386 (-0.069, 0.616) 0.432 (0.066, 0.630) | 0.554 |  | 0.172 |  | 0.33 | 0.232 |  | — |  | 0.04 0.30 | 0.884 0.180 |  |
|  | Entropy, nats |  | 3.91 [3.87, 3.94] 3.96 [3.85, 3.98] | 0.0346 0.0384 | 0.536 (0.210, 0.706) 0.578 (0.301, 0.727) | 0.440 |  | 0.544 |  | -0.44 | 0.126 |  | — |  | 0.31 0.01 | 0.160 0.963 |  |
|  | Max, % |  | 16.9 [14.8, 18.8] 19.8 [17.2, 23.0] | 4.07 3.35 | 0.281 (-0.271, 0.538) 0.534 (0.224, 0.699) | 0.087 |  | 0.617 |  | -0.90 | **0.000** | **0.004** | ✓ |  | -0.31 -0.06 | 0.244 0.790 |  |
|  | Prevalence, % |  | 3.21 [2.76, 3.50] 3.38 [3.00, 3.80] | 0.577 0.467 | 0.637 (0.376, 0.770) 0.612 (0.350, 0.750) | 0.948 |  | 0.887 |  | -0.84 | **0.004** | **0.023** | ✓ |  | -0.12 0.10 | 0.559 0.621 |  |
| Wake•N1•N2 | | | | | | | | | | | | | | | | | |
|  | AT05, min | NT1: NT2: | 58.0 [16.5, 81.5] 20.0 [12.0, 57.0] | 23.0 14.0 | 0.287 (-0.219, 0.549) 0.581 (0.271, 0.735) | 0.051 |  | 0.147 |  | 0.88 | **0.001** | **0.006** | ✓ |  | 0.08 -0.36 | 0.782 0.073 |  |
|  | AT10, min |  | 125 [90.3, 172] 86.0 [61.0, 126] | 27.5 32.0 | 0.444 (0.048, 0.652) 0.454 (0.086, 0.649) | 0.789 |  | 0.00 |  | 1.09 | **0.000** | **0.000** | ✓ |  | 0.02 -0.33 | 0.948 0.141 |  |
|  | AT30, min |  | 327 [282, 418] 299 [246, 353] | 55.5 116 | 0.596 (0.320, 0.749) 0.226 (-0.276, 0.496) | **0.015** | 0.105 | 0.260 |  | 0.97 | **0.000** | **0.002** | ✓ |  | -0.10 -0.36 | 0.650 0.167 |  |
|  | AT50(w), min |  | 29.4 [21.0, 37.0] 19.3 [15.1, 33.9] | 2.99 6.21 | 0.669 (0.410, 0.796) 0.804 (0.665, 0.875) | 0.210 |  | 0.585 |  | 1.25 | **0.000** | **0.001** | ✓ |  | 0.37 -0.03 | 0.069 0.816 |  |
|  | AT50, min |  | 533 [465, 588] 457 [400, 558] | 55.0 78.0 | 0.611 (0.332, 0.755) 0.515 (0.191, 0.686) | 0.521 |  | 0.429 |  | 0.85 | **0.002** | **0.011** | ✓ |  | 0.01 -0.13 | 0.978 0.548 |  |
|  | Entropy, nats |  | 3.99 [3.96, 4.02] 3.99 [3.96, 4.01] | 0.0340 0.0351 | 0.519 (0.181, 0.695) 0.042 (-0.585, 0.377) | **0.021** | 0.123 | 0.104 |  | 0.20 | 0.431 |  | — |  | -0.11 -0.21 | 0.639 0.471 |  |
|  | Max, % |  | 32.7 [32.1, 33.0] 32.5 [32.3, 32.8] | 0.478 0.718 | 0.008 (-0.754, 0.362) -0.046 (-0.598, 0.322) | 0.607 |  | 0.706 |  | 0.28 | 0.166 |  | — |  | 0.05 -0.15 | 0.864 0.624 |  |
|  | Prevalence, % |  | 5.59 [4.12, 6.72] 4.74 [3.33, 6.57] | 0.294 0.565 | 0.726 (0.510, 0.831) 0.668 (0.433, 0.788) | 0.715 |  | 0.777 |  | 0.92 | **0.004** | **0.020** | ✓ |  | 0.31 -0.10 | 0.100 0.598 |  |
| Wake•N1•N3 | | | | | | | | | | | | | | | | | |
|  | AT05, min | NT1: NT2: | 56.5 [26.5, 78.5] 32.0 [20.0, 56.0] | 15.0 17.0 | 0.245 (-0.300, 0.524) 0.597 (0.321, 0.741) | 0.083 |  | 0.538 |  | 0.87 | **0.001** | **0.006** | ✓ |  | 0.02 -0.21 | 0.944 0.291 |  |
|  | AT10, min |  | 100 [87.5, 119] 86.0 [58.0, 104] | 20.0 20.0 | 0.636 (0.364, 0.773) 0.382 (-0.034, 0.602) | 0.068 |  | 0.494 |  | 0.97 | **0.000** | **0.003** | ✓ |  | -0.24 -0.17 | 0.242 0.495 |  |
|  | AT30, min |  | 269 [243, 334] 258 [240, 313] | 36.0 72.0 | 0.680 (0.448, 0.799) 0.417 (0.035, 0.620) | **0.043** | 0.193 | 0.293 |  | 0.62 | **0.030** | 0.081 | ✓ |  | 0.03 -0.25 | 0.885 0.269 |  |
|  | AT50(w), min |  | 7.43 [5.13, 8.03] 5.22 [4.35, 8.99] | 1.14 1.48 | 0.633 (0.354, 0.771) 0.811 (0.681, 0.879) | 0.125 |  | 0.645 |  | 0.85 | **0.007** | **0.029** | ✓ |  | 0.50 0.14 | **0.011** 0.316 | 0.838 |
|  | AT50, min |  | 470 [416, 529] 438 [390, 509] | 38.5 33.0 | 0.553 (0.230, 0.719) 0.502 (0.179, 0.676) | 0.951 |  | 0.248 |  | 0.78 | **0.005** | **0.023** | ✓ |  | 0.11 -0.20 | 0.636 0.345 |  |
|  | Entropy, nats |  | 4.00 [3.93, 4.03] 3.97 [3.93, 4.01] | 0.0359 0.0507 | 0.260 (-0.337, 0.531) 0.234 (-0.263, 0.501) | 0.988 |  | 0.00 |  | 0.55 | **0.020** | 0.060 | ✗ |  | 0.36 0.06 | 0.188 0.812 |  |
|  | Max, % |  | 11.6 [10.2, 13.5] 11.3 [9.25, 15.1] | 3.18 1.96 | 0.336 (-0.161, 0.585) 0.402 (0.002, 0.613) | 0.751 |  | 0.594 |  | -0.07 | 0.788 |  | — |  | 0.01 -0.09 | 0.973 0.688 |  |
|  | Prevalence, % |  | 1.47 [1.17, 1.67] 1.29 [1.03, 1.88] | 0.134 0.251 | 0.807 (0.665, 0.879) 0.718 (0.522, 0.820) | 0.130 |  | 0.915 |  | 0.64 | **0.042** | 0.104 | ✓ |  | 0.21 0.06 | 0.156 0.715 |  |
| Wake•N1•REM | | | | | | | | | | | | | | | | | |
|  | AT05, min | NT1: NT2: | 28.5 [10.5, 111] 55.0 [21.0, 127] | 82.5 24.0 | -0.163 (-1.055, 0.253) 0.683 (0.474, 0.794) | **0.000** | **0.003** | 0.00 |  | 0.03 | 0.906 |  | — |  | 0.20 -0.05 | 0.570 0.790 |  |
|  | AT10, min |  | 121 [40.8, 201] 144 [107, 172] | 84.0 37.0 | 0.057 (-0.553, 0.408) 0.505 (0.185, 0.677) | **0.021** | 0.123 | 0.203 |  | -0.03 | 0.903 |  | — |  | -0.04 -0.28 | 0.903 0.183 |  |
|  | AT30, min |  | 376 [309, 458] 338 [301, 358] | 102 91.0 | 0.263 (-0.227, 0.533) 0.355 (-0.066, 0.580) | 0.952 |  | 0.268 |  | 0.53 | **0.031** | 0.081 | ✓ |  | -0.11 -0.11 | 0.703 0.663 |  |
|  | AT50(w), min |  | 20.1 [13.1, 24.9] 11.7 [9.66, 19.8] | 3.07 3.07 | 0.860 (0.758, 0.912) 0.829 (0.713, 0.890) | 0.773 |  | 0.768 |  | 1.27 | **0.000** | **0.001** | ✓ |  | 0.18 -0.10 | 0.160 0.459 |  |
|  | AT50, min |  | 565 [516, 644] 558 [435, 586] | 62.0 110 | 0.658 (0.413, 0.784) -0.085 (-0.831, 0.282) | **0.000** | **0.012** | 0.836 |  | 0.52 | **0.024** | 0.071 | ✓ |  | -0.05 -0.29 | 0.805 0.338 |  |
|  | Entropy, nats |  | 3.89 [3.85, 3.91] 3.91 [3.88, 3.94] | 0.0359 0.0399 | 0.479 (0.096, 0.674) 0.487 (0.153, 0.666) | 0.926 |  | 0.555 |  | -0.35 | 0.215 |  | — |  | 0.07 -0.29 | 0.779 0.171 |  |
|  | Max, % |  | 30.4 [29.5, 31.7] 30.4 [29.0, 31.2] | 0.751 1.73 | 0.776 (0.609, 0.860) 0.296 (-0.159, 0.541) | **0.001** | **0.020** | 0.113 |  | 0.93 | **0.001** | **0.005** | ✓ |  | 0.24 -0.02 | 0.151 0.945 |  |
|  | Prevalence, % |  | 3.40 [2.66, 4.51] 2.51 [2.00, 3.54] | 0.396 0.319 | 0.870 (0.773, 0.918) 0.842 (0.734, 0.899) | 0.961 |  | 0.882 |  | 1.22 | **0.000** | **0.002** | ✓ |  | 0.21 -0.08 | 0.087 0.526 |  |
| Wake•N2•N3 | | | | | | | | | | | | | | | | | |
|  | AT05, min | NT1: NT2: | 55.5 [36.3, 62.5] 45.0 [37.0, 56.0] | 17.5 9.00 | 0.266 (-0.294, 0.542) 0.583 (0.303, 0.731) | 0.064 |  | 0.00 |  | 0.27 | 0.347 |  | — |  | -0.16 -0.13 | 0.594 0.527 |  |
|  | AT10, min |  | 84.0 [73.0, 99.5] 84.0 [73.0, 97.0] | 15.0 18.0 | 0.361 (-0.127, 0.601) 0.549 (0.240, 0.711) | 0.617 |  | 0.00 |  | 0.17 | 0.564 |  | — |  | -0.10 -0.14 | 0.700 0.505 |  |
|  | AT30, min |  | 218 [195, 263] 258 [222, 306] | 27.0 47.0 | 0.570 (0.246, 0.731) 0.414 (0.022, 0.621) | 0.783 |  | 0.703 |  | -0.38 | 0.181 |  | — |  | 0.09 -0.30 | 0.694 0.198 |  |
|  | AT50(w), min |  | 16.2 [13.0, 17.9] 16.3 [14.7, 21.5] | 3.76 2.84 | 0.668 (0.425, 0.792) 0.561 (0.268, 0.716) | 0.593 |  | 0.853 |  | -0.47 | 0.115 |  | — |  | 0.32 0.01 | 0.097 0.954 |  |
|  | AT50, min |  | 394 [369, 418] 402 [375, 457] | 33.5 42.0 | 0.290 (-0.181, 0.558) 0.159 (-0.393, 0.453) | 0.377 |  | 0.766 |  | 0.15 | 0.541 |  | — |  | 0.26 -0.06 | 0.367 0.836 |  |
|  | Entropy, nats |  | 3.80 [3.77, 3.82] 3.82 [3.80, 3.86] | 0.0428 0.0209 | 0.193 (-0.328, 0.491) 0.465 (0.102, 0.654) | 0.165 |  | 0.00 |  | -0.10 | 0.709 |  | — |  | 0.71 0.04 | **0.013** 0.841 | 0.838 |
|  | Max, % |  | 27.2 [23.5, 32.1] 29.4 [21.8, 31.4] | 2.68 2.94 | 0.444 (0.044, 0.649) 0.468 (0.127, 0.653) | 0.870 |  | 0.137 |  | -0.27 | 0.336 |  | — |  | 0.01 0.17 | 0.971 0.433 |  |
|  | Prevalence, % |  | 3.95 [3.32, 4.58] 4.02 [3.71, 5.41] | 0.479 0.548 | 0.735 (0.541, 0.834) 0.621 (0.377, 0.753) | 0.531 |  | 0.765 |  | -0.53 | 0.075 |  | — |  | 0.12 0.21 | 0.505 0.260 |  |
| Wake•N2•REM | | | | | | | | | | | | | | | | | |
|  | AT05, min | NT1: NT2: | 26.5 [12.8, 96.5] 64.0 [31.0, 99.0] | 32.5 20.0 | -0.189 (-0.897, 0.249) 0.639 (0.407, 0.765) | **0.001** | **0.021** | 0.00 |  | -0.15 | 0.545 |  | — |  | 0.28 -0.10 | 0.437 0.589 |  |
|  | AT10, min |  | 121 [67.0, 176] 146 [123, 166] | 70.5 37.0 | -0.102 (-0.766, 0.300) 0.418 (0.043, 0.621) | **0.014** | 0.100 | 0.00 |  | -0.01 | 0.972 |  | — |  | 0.02 -0.38 | 0.943 0.094 |  |
|  | AT30, min |  | 334 [299, 383] 311 [283, 355] | 59.0 59.0 | 0.316 (-0.165, 0.567) 0.342 (-0.089, 0.572) | 0.723 |  | 0.646 |  | 0.53 | **0.033** | 0.084 | ✓ |  | 0.07 -0.03 | 0.805 0.888 |  |
|  | AT50(w), min |  | 23.0 [18.4, 26.0] 17.1 [13.6, 21.9] | 2.93 3.04 | 0.773 (0.602, 0.858) 0.845 (0.738, 0.901) | 0.284 |  | 0.851 |  | 0.83 | **0.011** | **0.039** | ✓ |  | 0.21 0.08 | 0.201 0.552 |  |
|  | AT50, min |  | 553 [497, 567] 503 [445, 532] | 46.5 64.0 | 0.589 (0.296, 0.740) 0.237 (-0.232, 0.502) | **0.020** | 0.123 | 0.785 |  | 0.64 | **0.012** | **0.042** | ✓ |  | 0.02 0.07 | 0.924 0.779 |  |
|  | Entropy, nats |  | 3.90 [3.87, 3.93] 3.91 [3.88, 3.95] | 0.0253 0.0437 | 0.573 (0.264, 0.730) 0.333 (-0.105, 0.567) | 0.180 |  | 0.719 |  | 0.05 | 0.859 |  | — |  | -0.04 -0.27 | 0.871 0.266 |  |
|  | Max, % |  | 27.7 [26.0, 28.2] 26.7 [23.9, 28.8] | 3.32 2.36 | 0.177 (-0.454, 0.471) 0.485 (0.142, 0.667) | 0.116 |  | 0.733 |  | -0.05 | 0.866 |  | — |  | -0.48 -0.03 | 0.087 0.880 |  |
|  | Prevalence, % |  | 3.99 [3.55, 4.49] 3.40 [3.04, 4.30] | 0.257 0.391 | 0.819 (0.686, 0.887) 0.809 (0.683, 0.877) | 0.910 |  | 0.906 |  | 0.49 | 0.130 |  | — |  | 0.31 -0.08 | **0.031** 0.572 | 0.838 |
| Wake•N3•REM | | | | | | | | | | | | | | | | | |
|  | AT05, min | NT1: NT2: | 31.5 [16.5, 73.5] 63.0 [44.0, 86.0] | 33.0 15.0 | -0.038 (-0.624, 0.346) 0.615 (0.365, 0.749) | **0.003** | **0.045** | 0.0392 |  | -0.37 | 0.149 |  | — |  | 0.18 -0.20 | 0.595 0.276 |  |
|  | AT10, min |  | 90.5 [60.5, 142] 119 [113, 131] | 40.5 25.0 | 0.023 (-0.661, 0.382) 0.603 (0.336, 0.743) | **0.014** | 0.100 | 0.0191 |  | -0.19 | 0.464 |  | — |  | 0.18 -0.33 | 0.582 0.080 |  |
|  | AT30, min |  | 282 [254, 347] 287 [262, 348] | 49.5 47.0 | 0.189 (-0.378, 0.486) 0.248 (-0.256, 0.513) | 0.448 |  | 0.219 |  | 0.29 | 0.233 |  | — |  | 0.25 -0.03 | 0.402 0.907 |  |
|  | AT50(w), min |  | 5.98 [5.42, 6.54] 4.80 [4.61, 5.82] | 1.06 1.21 | 0.798 (0.652, 0.872) 0.672 (0.453, 0.788) | 0.476 |  | 0.686 |  | 0.29 | 0.358 |  | — |  | 0.29 -0.01 | **0.042** 0.976 | 0.838 |
|  | AT50, min |  | 494 [427, 530] 470 [435, 505] | 34.5 52.0 | 0.680 (0.451, 0.798) 0.219 (-0.285, 0.491) | **0.004** | **0.048** | 0.0821 |  | 0.52 | **0.050** | 0.121 | ✓ |  | 0.30 -0.14 | 0.108 0.605 |  |
|  | Entropy, nats |  | 3.91 [3.88, 3.93] 3.92 [3.88, 3.96] | 0.0293 0.0588 | 0.479 (0.110, 0.670) 0.138 (-0.418, 0.439) | **0.031** | 0.157 | 0.416 |  | -0.38 | 0.128 |  | — |  | -0.32 -0.08 | 0.174 0.772 |  |
|  | Max, % |  | 7.80 [7.51, 8.44] 9.14 [7.80, 12.0] | 1.34 1.51 | 0.726 (0.522, 0.829) 0.338 (-0.090, 0.569) | **0.036** | 0.170 | 0.747 |  | -0.60 | **0.031** | 0.081 | ✗ |  | 0.02 -0.10 | 0.911 0.682 |  |
|  | Prevalence, % |  | 1.21 [0.993, 1.45] 1.08 [1.02, 1.46] | 0.153 0.211 | 0.801 (0.658, 0.874) 0.726 (0.543, 0.823) | 0.476 |  | 0.899 |  | 0.10 | 0.748 |  | — |  | 0.24 0.03 | 0.102 0.861 |  |
| N1•N2•N3•REM | | | | | | | | | | | | | | | | | |
|  | AT05, min | NT1: NT2: | 34.5 [27.5, 69.8] 50.0 [40.0, 74.0] | 16.0 14.0 | 0.206 (-0.361, 0.498) 0.631 (0.392, 0.760) | **0.028** | 0.144 | 0.265 |  | -0.42 | 0.128 |  | — |  | 0.01 -0.22 | 0.974 0.227 |  |
|  | AT10, min |  | 96.0 [74.8, 130] 117 [86.0, 132] | 27.5 29.0 | 0.321 (-0.160, 0.570) 0.584 (0.304, 0.731) | 0.210 |  | 0.615 |  | -0.16 | 0.566 |  | — |  | 0.08 -0.13 | 0.767 0.495 |  |
|  | AT30, min |  | 298 [268, 351] 289 [270, 321] | 42.5 45.0 | 0.530 (0.181, 0.706) 0.390 (-0.005, 0.602) | 0.141 |  | 0.751 |  | 0.19 | 0.506 |  | — |  | 0.00 0.06 | 1.000 0.784 |  |
|  | AT50(w), min |  | 12.6 [10.5, 13.7] 10.2 [9.56, 12.9] | 1.57 2.84 | 0.692 (0.473, 0.805) 0.641 (0.399, 0.768) | 0.831 |  | 0.873 |  | 0.19 | 0.555 |  | — |  | 0.16 0.03 | 0.381 0.862 |  |
|  | AT50, min |  | 497 [465, 562] 473 [429, 501] | 42.0 50.0 | 0.555 (0.222, 0.723) 0.266 (-0.207, 0.521) | 0.176 |  | 0.604 |  | 0.75 | **0.005** | **0.023** | ✓ |  | -0.00 0.08 | 0.999 0.751 |  |
|  | Entropy, nats |  | 3.96 [3.94, 3.98] 3.96 [3.93, 3.98] | 0.0285 0.0261 | 0.531 (0.141, 0.712) 0.420 (0.046, 0.622) | 0.470 |  | 0.793 |  | 0.17 | 0.564 |  | — |  | 0.05 0.17 | 0.849 0.463 |  |
|  | Max, % |  | 10.5 [9.69, 10.8] 10.9 [10.2, 12.2] | 1.29 1.06 | 0.718 (0.508, 0.824) 0.400 (0.004, 0.612) | **0.025** | 0.132 | 0.129 |  | -0.62 | **0.030** | 0.081 | ✗ |  | 0.14 -0.03 | 0.436 0.889 |  |
|  | Prevalence, % |  | 2.39 [2.26, 2.63] 2.35 [2.07, 2.62] | 0.318 0.302 | 0.754 (0.578, 0.845) 0.666 (0.442, 0.784) | 0.544 |  | 0.866 |  | -0.11 | 0.732 |  | — |  | 0.24 0.03 | 0.143 0.883 |  |
| Wake•N1•N2•N3 | | | | | | | | | | | | | | | | | |
|  | AT05, min | NT1: NT2: | 56.5 [31.0, 67.0] 33.0 [27.0, 44.0] | 23.5 10.0 | 0.318 (-0.176, 0.569) 0.683 (0.458, 0.799) | **0.009** | 0.074 | 0.369 |  | 0.84 | **0.002** | **0.011** | ✓ |  | -0.25 -0.01 | 0.356 0.974 |  |
|  | AT10, min |  | 105 [91.3, 121] 87.0 [77.0, 103] | 16.0 23.0 | 0.555 (0.240, 0.719) 0.185 (-0.295, 0.476) | 0.053 |  | 0.672 |  | 0.67 | **0.008** | **0.031** | ✗ |  | -0.13 -0.05 | 0.559 0.868 |  |
|  | AT30, min |  | 261 [248, 302] 276 [249, 314] | 35.5 60.0 | 0.655 (0.396, 0.785) 0.305 (-0.156, 0.550) | **0.016** | 0.110 | 0.205 |  | 0.36 | 0.211 |  | — |  | 0.11 -0.34 | 0.585 0.173 |  |
|  | AT50(w), min |  | 13.3 [10.9, 14.4] 11.0 [9.47, 17.0] | 1.77 2.65 | 0.835 (0.704, 0.899) 0.705 (0.510, 0.809) | 0.191 |  | 0.725 |  | 0.32 | 0.325 |  | — |  | 0.25 0.17 | 0.083 0.322 |  |
|  | AT50, min |  | 457 [415, 500] 459 [395, 491] | 36.5 21.0 | 0.478 (0.108, 0.670) 0.478 (0.131, 0.662) | 0.692 |  | 0.188 |  | 0.61 | **0.028** | 0.077 | ✓ |  | 0.22 -0.24 | 0.355 0.271 |  |
|  | Entropy, nats |  | 3.97 [3.91, 4.00] 3.94 [3.90, 3.96] | 0.0479 0.0233 | 0.175 (-0.408, 0.478) 0.082 (-0.576, 0.399) | 0.522 |  | 0.489 |  | 0.12 | 0.607 |  | — |  | -0.12 -0.00 | 0.682 0.989 |  |
|  | Max, % |  | 16.2 [13.8, 18.2] 16.2 [14.3, 19.1] | 3.26 2.39 | 0.423 (-0.003, 0.639) 0.433 (0.067, 0.630) | 0.791 |  | 0.764 |  | -0.09 | 0.734 |  | — |  | 0.00 -0.10 | 0.998 0.660 |  |
|  | Prevalence, % |  | 2.62 [2.39, 3.10] 2.61 [2.20, 3.69] | 0.275 0.415 | 0.816 (0.680, 0.884) 0.684 (0.474, 0.795) | 0.087 |  | 0.953 |  | 0.11 | 0.732 |  | — |  | 0.26 0.14 | 0.068 0.405 |  |
| Wake•N1•N2•REM | | | | | | | | | | | | | | | | | |
|  | AT05, min | NT1: NT2: | 28.0 [11.5, 88.3] 54.0 [21.0, 95.0] | 41.0 25.0 | -0.139 (-0.848, 0.280) 0.667 (0.451, 0.783) | **0.000** | **0.014** | 0.0374 |  | 0.09 | 0.717 |  | — |  | 0.34 -0.15 | 0.320 0.385 |  |
|  | AT10, min |  | 143 [69.5, 176] 146 [93.0, 162] | 63.0 26.0 | 0.068 (-0.560, 0.409) 0.392 (0.002, 0.604) | 0.093 |  | 0.409 |  | 0.18 | 0.460 |  | — |  | -0.05 -0.43 | 0.871 0.058 |  |
|  | AT30, min |  | 359 [301, 411] 318 [284, 350] | 66.5 91.0 | 0.450 (0.060, 0.652) 0.369 (-0.040, 0.589) | 0.584 |  | 0.604 |  | 0.64 | **0.013** | **0.043** | ✓ |  | -0.03 -0.14 | 0.913 0.551 |  |
|  | AT50(w), min |  | 22.3 [16.4, 24.8] 13.1 [11.6, 20.8] | 2.51 1.80 | 0.868 (0.761, 0.920) 0.831 (0.714, 0.892) | 0.802 |  | 0.845 |  | 1.02 | **0.002** | **0.012** | ✓ |  | 0.15 -0.11 | 0.248 0.423 |  |
|  | AT50, min |  | 551 [509, 616] 518 [431, 551] | 67.5 69.0 | 0.611 (0.334, 0.754) 0.257 (-0.214, 0.515) | 0.071 |  | 0.847 |  | 0.74 | **0.003** | **0.019** | ✓ |  | -0.00 -0.11 | 0.983 0.663 |  |
|  | Entropy, nats |  | 3.93 [3.91, 3.94] 3.93 [3.89, 3.98] | 0.0238 0.0554 | 0.477 (0.101, 0.670) 0.421 (0.042, 0.624) | 0.867 |  | 0.271 |  | 0.04 | 0.872 |  | — |  | 0.02 -0.24 | 0.950 0.292 |  |
|  | Max, % |  | 24.3 [23.7, 24.6] 22.8 [22.4, 24.3] | 0.649 0.910 | 0.631 (0.348, 0.771) 0.619 (0.364, 0.754) | 0.676 |  | 0.320 |  | 0.79 | **0.009** | **0.036** | ✗ |  | 0.05 0.16 | 0.815 0.402 |  |
|  | Prevalence, % |  | 4.00 [2.96, 4.44] 3.15 [2.70, 4.57] | 0.175 0.514 | 0.780 (0.608, 0.864) 0.761 (0.595, 0.847) | 0.647 |  | 0.874 |  | 0.90 | **0.006** | **0.026** | ✓ |  | 0.32 -0.12 | 0.051 0.427 |  |
| Wake•N1•N3•REM | | | | | | | | | | | | | | | | | |
|  | AT05, min | NT1: NT2: | 36.0 [12.8, 84.8] 54.0 [31.0, 85.0] | 38.5 18.0 | -0.167 (-0.776, 0.205) 0.659 (0.437, 0.778) | **0.000** | **0.012** | 0.00 |  | -0.04 | 0.862 |  | — |  | 0.19 -0.10 | 0.580 0.580 |  |
|  | AT10, min |  | 108 [59.8, 144] 136 [89.0, 147] | 54.5 27.0 | 0.025 (-0.582, 0.383) 0.437 (0.071, 0.633) | 0.079 |  | 0.322 |  | 0.07 | 0.789 |  | — |  | 0.13 -0.41 | 0.688 0.063 |  |
|  | AT30, min |  | 327 [278, 373] 307 [242, 344] | 56.5 43.0 | 0.280 (-0.224, 0.543) 0.393 (-0.006, 0.605) | 0.484 |  | 0.614 |  | 0.59 | **0.017** | 0.055 | ✓ |  | 0.19 -0.14 | 0.490 0.551 |  |
|  | AT50(w), min |  | 7.50 [5.80, 8.66] 5.47 [4.52, 6.34] | 1.15 1.34 | 0.863 (0.753, 0.916) 0.768 (0.607, 0.852) | 0.563 |  | 0.755 |  | 0.83 | **0.012** | **0.043** | ✓ |  | 0.21 0.07 | 0.123 0.642 |  |
|  | AT50, min |  | 541 [473, 562] 480 [420, 517] | 49.0 60.0 | 0.564 (0.252, 0.724) 0.255 (-0.198, 0.514) | 0.075 |  | 0.745 |  | 0.70 | **0.006** | **0.026** | ✓ |  | 0.04 -0.04 | 0.849 0.869 |  |
|  | Entropy, nats |  | 3.91 [3.90, 3.94] 3.93 [3.89, 3.97] | 0.0344 0.0579 | 0.494 (0.131, 0.681) 0.426 (0.051, 0.627) | 0.678 |  | 0.687 |  | -0.23 | 0.405 |  | — |  | -0.04 -0.18 | 0.867 0.428 |  |
|  | Max, % |  | 8.45 [8.29, 9.21] 7.89 [7.25, 9.13] | 0.549 1.57 | 0.612 (0.333, 0.755) 0.261 (-0.215, 0.518) | **0.018** | 0.119 | 0.709 |  | -0.08 | 0.775 |  | — |  | -0.36 0.05 | 0.080 0.859 |  |
|  | Prevalence, % |  | 1.42 [1.18, 1.63] 1.19 [0.993, 1.59] | 0.0977 0.187 | 0.842 (0.726, 0.901) 0.836 (0.720, 0.896) | 0.794 |  | 0.816 |  | 0.70 | **0.034** | 0.085 | ✓ |  | 0.24 -0.14 | 0.070 0.305 |  |
| Wake•N2•N3•REM | | | | | | | | | | | | | | | | | |
|  | AT05, min | NT1: NT2: | 39.0 [31.5, 57.5] 47.0 [40.0, 71.0] | 13.0 14.0 | 0.454 (0.049, 0.658) 0.553 (0.263, 0.709) | 0.579 |  | 0.340 |  | -0.40 | 0.161 |  | — |  | 0.01 -0.21 | 0.984 0.305 |  |
|  | AT10, min |  | 101 [77.3, 111] 108 [84.0, 122] | 15.5 41.0 | 0.384 (-0.055, 0.610) 0.329 (-0.105, 0.563) | 0.772 |  | 0.464 |  | -0.08 | 0.763 |  | — |  | 0.09 -0.24 | 0.734 0.330 |  |
|  | AT30, min |  | 289 [254, 309] 277 [265, 328] | 33.5 34.0 | 0.531 (0.182, 0.706) 0.321 (-0.120, 0.558) | 0.278 |  | 0.613 |  | 0.05 | 0.864 |  | — |  | 0.02 -0.07 | 0.940 0.771 |  |
|  | AT50(w), min |  | 10.9 [9.13, 12.0] 10.1 [9.12, 11.6] | 1.87 2.34 | 0.710 (0.502, 0.816) 0.669 (0.448, 0.786) | 0.818 |  | 0.819 |  | 0.02 | 0.961 |  | — |  | 0.22 0.03 | 0.219 0.865 |  |
|  | AT50, min |  | 468 [425, 520] 460 [421, 494] | 36.5 45.0 | 0.475 (0.088, 0.672) 0.355 (-0.061, 0.580) | 0.582 |  | 0.565 |  | 0.63 | **0.019** | 0.059 | ✗ |  | 0.21 -0.00 | 0.405 0.993 |  |
|  | Entropy, nats |  | 3.96 [3.92, 3.98] 3.93 [3.89, 3.97] | 0.0279 0.0262 | 0.406 (0.052, 0.631) 0.240 (-0.248, 0.504) | 0.433 |  | 0.620 |  | 0.24 | 0.374 |  | — |  | -0.01 0.36 | 0.984 0.168 |  |
|  | Max, % |  | 10.3 [10.1, 11.7] 12.9 [11.5, 15.8] | 1.89 2.79 | 0.339 (-0.169, 0.575) 0.557 (0.265, 0.713) | 0.071 |  | 0.586 |  | -0.80 | **0.002** | **0.011** | ✗ |  | 0.04 -0.16 | 0.880 0.432 |  |
|  | Prevalence, % |  | 2.18 [1.97, 2.56] 2.30 [1.99, 2.71] | 0.269 0.300 | 0.826 (0.698, 0.891) 0.705 (0.508, 0.809) | 0.275 |  | 0.857 |  | -0.26 | 0.425 |  | — |  | 0.15 0.07 | 0.265 0.674 |  |
| Wake•N1•N2•N3•REM | | | | | | | | | | | | | | | | | |
|  | AT05, min | NT1: NT2: | 34.5 [22.3, 59.8] 43.0 [31.0, 78.0] | 14.5 15.0 | 0.161 (-0.442, 0.471) 0.687 (0.478, 0.797) | **0.021** | 0.123 | 0.270 |  | -0.08 | 0.764 |  | — |  | 0.20 -0.14 | 0.503 0.395 |  |
|  | AT10, min |  | 109 [86.0, 132] 112 [91.0, 131] | 22.5 34.0 | 0.429 (0.010, 0.641) 0.406 (0.022, 0.613) | 0.649 |  | 0.456 |  | 0.27 | 0.321 |  | — |  | 0.15 -0.32 | 0.542 0.161 |  |
|  | AT30, min |  | 306 [270, 345] 298 [240, 332] | 35.5 56.0 | 0.436 (0.038, 0.643) 0.278 (-0.192, 0.530) | 0.208 |  | 0.662 |  | 0.55 | **0.032** | 0.081 | ✓ |  | 0.17 -0.23 | 0.493 0.365 |  |
|  | AT50(w), min |  | 11.4 [9.48, 12.3] 9.10 [7.56, 10.8] | 1.63 2.07 | 0.787 (0.630, 0.866) 0.773 (0.622, 0.853) | 0.738 |  | 0.842 |  | 0.51 | 0.110 |  | — |  | 0.17 -0.00 | 0.282 0.999 |  |
|  | AT50, min |  | 516 [460, 541] 462 [420, 504] | 42.0 58.0 | 0.604 (0.320, 0.750) 0.330 (-0.102, 0.563) | 0.082 |  | 0.631 |  | 0.67 | **0.012** | **0.043** | ✓ |  | 0.04 0.00 | 0.840 0.990 |  |
|  | Entropy, nats |  | 3.97 [3.92, 3.99] 3.97 [3.93, 3.98] | 0.0378 0.0379 | 0.349 (-0.163, 0.584) 0.261 (-0.218, 0.519) | 0.615 |  | 0.889 |  | -0.05 | 0.836 |  | — |  | -0.20 0.14 | 0.435 0.576 |  |
|  | Max, % |  | 10.6 [9.93, 11.2] 10.6 [8.72, 11.9] | 1.26 2.37 | 0.638 (0.369, 0.773) 0.293 (-0.174, 0.542) | 0.099 |  | 0.249 |  | -0.30 | 0.284 |  | — |  | -0.09 0.03 | 0.662 0.902 |  |
|  | Prevalence, % |  | 2.09 [1.91, 2.51] 1.86 [1.72, 2.72] | 0.167 0.442 | 0.884 (0.796, 0.928) 0.714 (0.524, 0.815) | 0.033 | 0.158 | 0.866 |  | 0.26 | 0.422 |  | — |  | 0.19 0.00 | 0.111 0.984 |  |
| AT, accumulation time. *Statistics*: Median and IQR of observations at baseline, Δ indicates median absolute difference between observations at baseline and week 4. *Reliability*: Estimated reliability with 95% confidence intervals; *p*, the *p*-value of Levene's test; Val., the estimated reliability in the validation cohort. *Diagnosis effect*: Effect size (NT2 versus NT1) in standardized units; *p*, the *p*-value of a t-test; Val., a check (✓) indicates the diagnosis effect is significant in the validation cohort, an ex (✗) indicates the diagnosis effect is not significant in the validation cohort, a dash (—) indicates validation of the diagnosis effect was not tested. *Study visit effect*: Comparison of values at baseline and while "on-placebo"; *p*, the *p*-value of a t-test; *FDR*: The false discovery rate (FDR) adjusted *p*-value. For each feature, results for NT1 are shown on the first line and NT2 are shown in the second line. | | | | | | | | | | | | | | | | | |

| **qEEG features** | | | | | | | | | | | | | | | | | |
| --- | --- | --- | --- | --- | --- | --- | --- | --- | --- | --- | --- | --- | --- | --- | --- | --- | --- |
|  | **Band** |  | **Statistics** | | **Reliability** | | | |  | **Diagnosis effect** | | | |  | **Study visit effect** | | |
|  |  |  | Median [IQR] | Δ | Est. (95% CI) | *p* | FDR | Val. |  | NT1 eff. (SD) | *p* | FDR | Val. |  | Est. | *p* | FDR |
| N1 - Baseline | | | | | | | | | | | | | | | | | |
|  | Alpha | NT1: NT2: | 0.777 [0.655, 2.53] 1.01 [0.770, 1.28] | 0.234 0.223 | 0.877 (0.785, 0.924) 0.883 (0.795, 0.927) | 0.946 |  | 0.908 |  | 0.12 | 0.733 |  | — |  | 0.14 0.12 | 0.260 0.282 |  |
|  | Beta |  | 0.175 [0.138, 0.353] 0.148 [0.119, 0.195] | 0.0313 0.0611 | 0.836 (0.713, 0.898) 0.386 (-0.083, 0.615) | **0.004** | 0.052 | 0.850 |  | -0.30 | 0.343 |  | — |  | -0.21 -0.08 | 0.133 0.753 |  |
|  | Delta |  | 4.12 [3.22, 5.63] 3.92 [3.67, 5.62] | 0.527 1.44 | 0.786 (0.626, 0.867) 0.569 (0.250, 0.729) | **0.007** | 0.061 | 0.531 |  | -0.25 | 0.444 |  | — |  | 0.05 0.10 | 0.779 0.650 |  |
|  | Gamma |  | 0.0493 [0.0361, 0.0697] 0.0345 [0.0232, 0.0533] | 0.0137 0.0181 | 0.704 (0.470, 0.818) 0.382 (-0.120, 0.619) | 0.071 |  | 0.557 |  | -0.27 | 0.396 |  | — |  | -0.32 0.08 | 0.087 0.745 |  |
|  | Sigma |  | 0.440 [0.341, 0.767] 0.490 [0.379, 0.715] | 0.103 0.0935 | 0.916 (0.852, 0.948) 0.793 (0.642, 0.869) | 0.110 |  | 0.858 |  | 0.00 | 0.992 |  | — |  | 0.09 -0.09 | 0.348 0.540 |  |
|  | Theta |  | 1.44 [1.08, 2.41] 1.67 [1.16, 2.02] | 0.265 0.208 | 0.816 (0.682, 0.885) 0.818 (0.688, 0.884) | 0.859 |  | 0.826 |  | 0.26 | 0.402 |  | — |  | -0.11 0.10 | 0.448 0.462 |  |
| N1 - Mean | | | | | | | | | | | | | | | | | |
|  | Alpha | NT1: NT2: | 0.118 [0.0989, 0.158] 0.119 [0.0915, 0.138] | 0.0128 0.0217 | 0.866 (0.765, 0.917) 0.753 (0.582, 0.842) | 0.282 |  | 0.935 |  | 0.65 | **0.031** | 0.318 | ✗ |  | 0.07 0.27 | 0.583 0.079 |  |
|  | Beta |  | 0.0952 [0.0818, 0.111] 0.0877 [0.0645, 0.112] | 0.0152 0.0315 | 0.718 (0.505, 0.824) 0.336 (-0.120, 0.575) | **0.038** | 0.132 | 0.812 |  | -0.06 | 0.838 |  | — |  | -0.12 0.10 | 0.521 0.678 |  |
|  | Delta |  | 0.478 [0.415, 0.559] 0.527 [0.459, 0.564] | 0.0360 0.0504 | 0.880 (0.789, 0.925) 0.626 (0.368, 0.761) | **0.004** | 0.052 | 0.948 |  | -0.82 | **0.005** | 0.266 | ✗ |  | 0.04 -0.30 | 0.735 0.113 |  |
|  | Gamma |  | 0.00852 [0.00421, 0.0130] 0.00771 [0.00511, 0.00840] | 0.00184 0.00229 | 0.828 (0.698, 0.893) 0.606 (0.333, 0.748) | 0.077 |  | 0.822 |  | -0.36 | 0.254 |  | — |  | -0.21 0.26 | 0.144 0.179 |  |
|  | Sigma |  | 0.0520 [0.0495, 0.0619] 0.0514 [0.0449, 0.0640] | 0.00605 0.00924 | 0.885 (0.798, 0.928) 0.607 (0.340, 0.748) | **0.007** | 0.061 | 0.942 |  | 0.37 | 0.228 |  | — |  | 0.23 0.16 | 0.055 0.393 |  |
|  | Theta |  | 0.210 [0.183, 0.218] 0.200 [0.149, 0.209] | 0.0179 0.0262 | 0.779 (0.612, 0.862) 0.756 (0.580, 0.845) | 0.254 |  | 0.865 |  | 0.66 | **0.043** | 0.352 | ✓ |  | 0.01 0.14 | 0.967 0.370 |  |
| N1 - Peak Amp | | | | | | | | | | | | | | | | | |
|  | Alpha | NT1: NT2: | 2.14 [1.90, 2.40] 2.12 [1.79, 2.28] | 0.236 0.220 | 0.845 (0.732, 0.902) 0.859 (0.759, 0.910) | 0.500 |  | 0.797 |  | 0.29 | 0.395 |  | — |  | -0.07 -0.00 | 0.584 0.971 |  |
|  | Beta |  | 3.04 [2.48, 4.34] 3.20 [2.78, 4.42] | 0.774 0.741 | 0.854 (0.748, 0.908) 0.709 (0.510, 0.813) | 0.144 |  | 0.887 |  | 0.21 | 0.514 |  | — |  | 0.11 -0.00 | 0.375 0.985 |  |
|  | Delta |  | 3.38 [2.33, 3.94] 3.66 [1.88, 6.35] | 0.672 3.11 | 0.722 (0.508, 0.827) -0.257 (-0.940, 0.201) | **0.001** | **0.035** | 0.969 |  | -0.59 | **0.011** | 0.266 | ✓ |  | -0.09 0.19 | 0.606 0.588 |  |
|  | Gamma |  | 2.39 [2.29, 2.83] 2.65 [2.31, 3.04] | 0.274 0.257 | 0.757 (0.572, 0.849) 0.788 (0.642, 0.865) | 0.281 |  | 0.503 |  | 0.28 | 0.380 |  | — |  | 0.22 -0.11 | 0.179 0.450 |  |
|  | Sigma |  | 1.66 [1.52, 1.77] 1.87 [1.64, 2.01] | 0.0905 0.187 | 0.706 (0.484, 0.817) 0.680 (0.463, 0.794) | 0.621 |  | 0.847 |  | -0.63 | **0.044** | 0.352 | ✓ |  | 0.45 0.09 | **0.011** 0.614 | 0.434 |
|  | Theta |  | 1.91 [1.80, 2.04] 2.12 [1.67, 2.26] | 0.161 0.157 | 0.857 (0.748, 0.911) 0.506 (0.176, 0.682) | **0.021** | 0.099 | 0.762 |  | -0.60 | **0.043** | 0.352 | ✗ |  | -0.10 0.03 | 0.435 0.878 |  |
| N1 - Peak Freq | | | | | | | | | | | | | | | | | |
|  | Alpha | NT1: NT2: | 8.62 [8.48, 8.82] 8.75 [8.56, 9.22] | 0.259 0.206 | 0.786 (0.630, 0.865) 0.746 (0.565, 0.839) | 0.712 |  | 0.927 |  | -0.57 | 0.084 |  | — |  | 0.23 0.13 | 0.126 0.424 |  |
|  | Beta |  | 17.8 [17.3, 18.8] 17.5 [17.2, 18.4] | 0.528 0.521 | 0.858 (0.756, 0.910) 0.522 (0.194, 0.693) | **0.003** | 0.051 | 0.847 |  | -0.39 | 0.194 |  | — |  | -0.10 0.47 | 0.437 **0.019** | 0.607 |
|  | Delta |  | 1.02 [0.930, 1.23] 1.00 [0.934, 1.19] | 0.0712 0.120 | 0.711 (0.481, 0.822) 0.726 (0.532, 0.826) | 0.663 |  | 0.745 |  | 0.19 | 0.549 |  | — |  | 0.08 0.04 | 0.688 0.822 |  |
|  | Gamma |  | 30.3 [30.2, 30.5] 30.5 [30.2, 30.7] | 0.0852 0.102 | 0.699 (0.453, 0.816) 0.615 (0.356, 0.752) | 0.417 |  | 0.620 |  | -0.73 | **0.016** | 0.316 | ✗ |  | 0.08 0.10 | 0.696 0.581 |  |
|  | Sigma |  | 13.0 [12.5, 13.5] 12.9 [12.5, 13.3] | 0.154 0.112 | 0.893 (0.814, 0.933) 0.875 (0.790, 0.919) | 0.502 |  | 0.771 |  | 0.06 | 0.851 |  | — |  | -0.35 -0.02 | **0.000** 0.887 | 0.060 |
|  | Theta |  | 4.70 [4.45, 5.27] 4.71 [4.37, 5.11] | 0.217 0.335 | 0.780 (0.622, 0.861) 0.835 (0.723, 0.894) | 0.464 |  | 0.871 |  | 0.43 | 0.163 |  | — |  | 0.21 0.31 | 0.172 **0.008** | 0.607 |
| N2 - Baseline | | | | | | | | | | | | | | | | | |
|  | Alpha | NT1: NT2: | 1.35 [0.799, 1.84] 1.40 [0.991, 1.95] | 0.196 0.199 | 0.902 (0.828, 0.939) 0.805 (0.665, 0.877) | 0.410 |  | 0.924 |  | 0.20 | 0.554 |  | — |  | 0.11 0.00 | 0.300 0.977 |  |
|  | Beta |  | 0.0733 [0.0510, 0.120] 0.0771 [0.0609, 0.121] | 0.0175 0.0152 | 0.792 (0.635, 0.871) 0.084 (-0.573, 0.419) | 0.182 |  | 0.810 |  | -0.19 | 0.521 |  | — |  | -0.03 0.18 | 0.826 0.540 |  |
|  | Delta |  | 9.90 [6.83, 11.6] 11.2 [8.27, 14.8] | 1.16 3.17 | 0.898 (0.821, 0.937) 0.782 (0.633, 0.861) | **0.014** | 0.080 | 0.752 |  | -0.40 | 0.228 |  | — |  | -0.08 -0.05 | 0.479 0.744 |  |
|  | Gamma |  | 0.0189 [0.0114, 0.0291] 0.0193 [0.0137, 0.0273] | 0.00306 0.00363 | 0.890 (0.805, 0.932) 0.163 (-0.436, 0.469) | **0.017** | 0.090 | 0.648 |  | -0.38 | 0.199 |  | — |  | -0.15 0.09 | 0.196 0.745 |  |
|  | Sigma |  | 0.660 [0.495, 1.17] 0.753 [0.430, 1.07] | 0.131 0.184 | 0.910 (0.841, 0.944) 0.644 (0.401, 0.772) | **0.044** | 0.138 | 0.811 |  | 0.09 | 0.787 |  | — |  | 0.07 0.09 | 0.487 0.638 |  |
|  | Theta |  | 2.37 [1.66, 3.47] 3.18 [1.75, 4.31] | 0.544 0.401 | 0.929 (0.876, 0.955) 0.946 (0.909, 0.965) | 0.515 |  | 0.932 |  | -0.14 | 0.673 |  | — |  | 0.07 -0.01 | 0.460 0.855 |  |
| N2 - Mean | | | | | | | | | | | | | | | | | |
|  | Alpha | NT1: NT2: | 0.0894 [0.0768, 0.124] 0.0885 [0.0791, 0.115] | 0.0121 0.0124 | 0.830 (0.704, 0.893) 0.579 (0.306, 0.727) | **0.038** | 0.132 | 0.970 |  | 0.50 | 0.078 |  | — |  | 0.28 0.15 | **0.045** 0.446 | 0.603 |
|  | Beta |  | 0.0409 [0.0344, 0.0479] 0.0338 [0.0242, 0.0406] | 0.00469 0.00546 | 0.670 (0.421, 0.793) 0.680 (0.453, 0.796) | 0.528 |  | 0.796 |  | 0.71 | **0.023** | 0.318 | ✗ |  | 0.03 -0.07 | 0.865 0.706 |  |
|  | Delta |  | 0.636 [0.567, 0.675] 0.628 [0.603, 0.672] | 0.0400 0.0519 | 0.884 (0.796, 0.928) 0.470 (0.125, 0.656) | **0.002** | 0.051 | 0.881 |  | -0.59 | **0.047** | 0.353 | ✗ |  | -0.21 -0.13 | 0.083 0.553 |  |
|  | Gamma |  | 0.00249 [0.00193, 0.00380] 0.00258 [0.00122, 0.00341] | 0.000421 0.000751 | 0.883 (0.793, 0.927) 0.688 (0.469, 0.801) | 0.080 |  | 0.857 |  | -0.08 | 0.791 |  | — |  | -0.11 -0.03 | 0.372 0.855 |  |
|  | Sigma |  | 0.0595 [0.0430, 0.0735] 0.0431 [0.0355, 0.0829] | 0.00957 0.00846 | 0.897 (0.820, 0.936) 0.841 (0.731, 0.898) | 0.288 |  | 0.807 |  | 0.30 | 0.382 |  | — |  | 0.23 0.08 | **0.032** 0.543 | 0.596 |
|  | Theta |  | 0.177 [0.169, 0.183] 0.176 [0.137, 0.191] | 0.0166 0.0215 | 0.773 (0.606, 0.858) 0.618 (0.357, 0.754) | 0.329 |  | 0.943 |  | 0.54 | 0.075 |  | — |  | 0.08 0.09 | 0.643 0.648 |  |
| N2 - Peak Amp | | | | | | | | | | | | | | | | | |
|  | Alpha | NT1: NT2: | 1.75 [1.62, 2.16] 2.24 [1.89, 2.57] | 0.0853 0.175 | 0.973 (0.952, 0.983) 0.887 (0.807, 0.928) | **0.006** | 0.061 | 0.938 |  | -0.29 | 0.426 |  | — |  | 0.04 0.07 | 0.439 0.492 |  |
|  | Beta |  | 5.20 [3.93, 6.71] 5.11 [3.61, 6.05] | 0.320 0.923 | 0.918 (0.856, 0.949) 0.761 (0.594, 0.848) | 0.124 |  | 0.804 |  | 0.17 | 0.631 |  | — |  | 0.14 0.10 | 0.166 0.512 |  |
|  | Delta |  | 3.40 [2.67, 3.80] 3.89 [2.74, 5.58] | 0.336 0.457 | 0.768 (0.599, 0.855) 0.718 (0.524, 0.820) | 0.703 |  | 0.910 |  | -0.84 | **0.005** | 0.266 | ✓ |  | 0.02 0.12 | 0.884 0.486 |  |
|  | Gamma |  | 2.45 [2.27, 2.55] 2.57 [2.34, 2.99] | 0.119 0.117 | 0.873 (0.777, 0.921) 0.879 (0.795, 0.922) | 0.851 |  | 0.790 |  | 0.01 | 0.984 |  | — |  | 0.23 0.20 | 0.063 0.063 |  |
|  | Sigma |  | 2.31 [1.81, 2.71] 2.07 [1.85, 2.52] | 0.229 0.637 | 0.851 (0.742, 0.907) 0.656 (0.410, 0.783) | **0.042** | 0.138 | 0.866 |  | -0.20 | 0.541 |  | — |  | 0.19 0.39 | 0.134 **0.037** | 0.607 |
|  | Theta |  | 2.32 [1.76, 2.79] 2.06 [1.85, 2.51] | 0.141 0.172 | 0.859 (0.755, 0.911) 0.781 (0.631, 0.860) | 0.596 |  | 0.927 |  | -0.35 | 0.248 |  | — |  | -0.15 0.15 | 0.249 0.311 |  |
| N2 - Peak Freq | | | | | | | | | | | | | | | | | |
|  | Alpha | NT1: NT2: | 9.00 [8.82, 9.15] 8.66 [8.50, 9.36] | 0.254 0.191 | 0.869 (0.771, 0.918) 0.860 (0.764, 0.911) | 0.958 |  | 0.959 |  | -0.02 | 0.950 |  | — |  | 0.18 0.15 | 0.142 0.178 |  |
|  | Beta |  | 16.6 [16.3, 17.0] 16.4 [16.3, 16.8] | 0.162 0.234 | 0.901 (0.828, 0.938) 0.603 (0.342, 0.743) | **0.007** | 0.061 | 0.815 |  | -0.32 | 0.302 |  | — |  | -0.09 0.30 | 0.379 0.116 |  |
|  | Delta |  | 1.07 [1.01, 1.29] 1.04 [0.959, 1.29] | 0.0868 0.0604 | 0.833 (0.711, 0.895) 0.726 (0.529, 0.826) | 0.961 |  | 0.966 |  | 0.51 | 0.117 |  | — |  | -0.10 0.07 | 0.487 0.667 |  |
|  | Gamma |  | 30.3 [30.2, 30.4] 30.3 [30.2, 30.4] | 0.0595 0.0551 | 0.945 (0.901, 0.966) 0.915 (0.854, 0.946) | 0.135 |  | 0.773 |  | -0.57 | 0.104 |  | — |  | -0.05 -0.00 | 0.592 0.963 |  |
|  | Sigma |  | 13.0 [12.8, 13.1] 12.8 [12.7, 13.2] | 0.224 0.164 | 0.665 (0.422, 0.788) 0.663 (0.437, 0.783) | 0.934 |  | 0.608 |  | -0.21 | 0.486 |  | — |  | -0.24 -0.00 | 0.216 0.997 |  |
|  | Theta |  | 4.38 [4.07, 4.56] 4.42 [4.15, 4.68] | 0.109 0.198 | 0.836 (0.711, 0.898) 0.827 (0.709, 0.889) | 0.844 |  | 0.770 |  | 0.19 | 0.551 |  | — |  | 0.11 0.13 | 0.433 0.291 |  |
| N3 - Baseline | | | | | | | | | | | | | | | | | |
|  | Alpha | NT1: NT2: | 1.63 [1.01, 2.29] 1.46 [0.901, 2.37] | 0.188 0.316 | 0.859 (0.753, 0.913) 0.891 (0.803, 0.933) | 0.589 |  | 0.931 |  | 0.45 | 0.200 |  | — |  | 0.04 0.06 | 0.785 0.605 |  |
|  | Beta |  | 0.0448 [0.0336, 0.0837] 0.0502 [0.0408, 0.0584] | 0.0175 0.0162 | 0.835 (0.714, 0.897) 0.644 (0.381, 0.777) | 0.316 |  | 0.766 |  | -0.28 | 0.400 |  | — |  | -0.02 0.22 | 0.861 0.255 |  |
|  | Delta |  | 25.3 [20.8, 29.4] 23.7 [15.8, 33.3] | 5.54 5.16 | 0.741 (0.550, 0.838) 0.809 (0.665, 0.881) | 0.323 |  | 0.899 |  | -0.18 | 0.580 |  | — |  | -0.16 -0.10 | 0.361 0.488 |  |
|  | Gamma |  | 0.0108 [0.00774, 0.0215] 0.0122 [0.0104, 0.0189] | 0.00191 0.00378 | 0.915 (0.850, 0.948) 0.670 (0.424, 0.793) | **0.003** | 0.052 | 0.778 |  | -0.44 | 0.186 |  | — |  | 0.02 0.16 | 0.836 0.402 |  |
|  | Sigma |  | 0.662 [0.413, 0.913] 0.532 [0.361, 0.827] | 0.111 0.181 | 0.889 (0.806, 0.931) 0.702 (0.485, 0.813) | 0.067 |  | 0.748 |  | 0.02 | 0.961 |  | — |  | -0.00 -0.03 | 0.980 0.872 |  |
|  | Theta |  | 3.85 [2.93, 6.31] 3.44 [2.35, 7.18] | 0.796 0.483 | 0.915 (0.851, 0.947) 0.937 (0.890, 0.960) | 0.199 |  | 0.939 |  | 0.06 | 0.861 |  | — |  | 0.02 -0.03 | 0.812 0.764 |  |
| N3 - Mean | | | | | | | | | | | | | | | | | |
|  | Alpha | NT1: NT2: | 0.0455 [0.0376, 0.0616] 0.0418 [0.0324, 0.0515] | 0.00535 0.00516 | 0.816 (0.680, 0.885) 0.580 (0.281, 0.734) | **0.014** | 0.080 | 0.923 |  | 0.62 | **0.032** | 0.318 | ✗ |  | 0.29 0.11 | **0.040** 0.604 | 0.603 |
|  | Beta |  | 0.00972 [0.00633, 0.0119] 0.00683 [0.00487, 0.00917] | 0.00198 0.00192 | 0.789 (0.629, 0.869) 0.332 (-0.151, 0.580) | **0.008** | 0.061 | 0.247 |  | 0.21 | 0.496 |  | — |  | 0.05 0.21 | 0.737 0.433 |  |
|  | Delta |  | 0.804 [0.761, 0.825] 0.821 [0.779, 0.839] | 0.0191 0.0132 | 0.821 (0.685, 0.889) 0.494 (0.134, 0.680) | **0.002** | 0.051 | 0.775 |  | -0.66 | **0.024** | 0.318 | ✗ |  | -0.15 -0.02 | 0.323 0.925 |  |
|  | Gamma |  | 0.000667 [0.000340, 0.000881] 0.000598 [0.000307, 0.000813] | 0.000119 0.000222 | 0.794 (0.642, 0.871) 0.389 (-0.058, 0.617) | **0.008** | 0.061 | 0.436 |  | -0.18 | 0.575 |  | — |  | 0.04 0.20 | 0.777 0.436 |  |
|  | Sigma |  | 0.0216 [0.0143, 0.0276] 0.0144 [0.0104, 0.0239] | 0.00352 0.00582 | 0.868 (0.769, 0.918) 0.717 (0.509, 0.823) | 0.073 |  | 0.871 |  | 0.33 | 0.312 |  | — |  | 0.08 0.26 | 0.532 0.139 |  |
|  | Theta |  | 0.116 [0.102, 0.129] 0.113 [0.104, 0.127] | 0.0120 0.0124 | 0.876 (0.780, 0.923) 0.658 (0.405, 0.785) | **0.031** | 0.121 | 0.794 |  | 0.53 | 0.098 |  | — |  | 0.02 -0.14 | 0.865 0.466 |  |
| N3 - Peak Amp | | | | | | | | | | | | | | | | | |
|  | Alpha | NT1: NT2: | 1.89 [1.69, 2.34] 2.27 [1.75, 3.03] | 0.179 0.190 | 0.881 (0.790, 0.926) 0.799 (0.651, 0.874) | 0.298 |  | 0.890 |  | -0.41 | 0.221 |  | — |  | -0.14 -0.21 | 0.229 0.150 |  |
|  | Beta |  | 5.37 [3.71, 7.47] 5.51 [4.70, 7.10] | 0.449 0.899 | 0.814 (0.673, 0.884) 0.680 (0.446, 0.799) | 0.403 |  | 0.927 |  | 0.48 | 0.151 |  | — |  | -0.14 -0.17 | 0.346 0.373 |  |
|  | Delta |  | 5.56 [3.40, 7.12] 6.05 [5.11, 7.55] | 0.631 1.55 | 0.847 (0.731, 0.905) 0.542 (0.201, 0.713) | **0.029** | 0.119 | 0.875 |  | -0.72 | **0.023** | 0.318 | ✗ |  | 0.00 0.10 | 0.983 0.664 |  |
|  | Gamma |  | 2.29 [2.15, 2.42] 2.40 [2.16, 2.61] | 0.197 0.148 | 0.829 (0.689, 0.896) 0.558 (0.233, 0.723) | 0.087 |  | 0.872 |  | -0.32 | 0.330 |  | — |  | 0.31 0.44 | **0.035** **0.037** | 0.596 0.607 |
|  | Sigma |  | 2.67 [2.09, 3.09] 2.03 [1.82, 2.44] | 0.338 0.245 | 0.872 (0.776, 0.920) 0.683 (0.456, 0.800) | **0.043** | 0.138 | 0.875 |  | 0.35 | 0.290 |  | — |  | 0.18 0.24 | 0.133 0.182 |  |
|  | Theta |  | 2.64 [2.17, 3.08] 2.39 [2.11, 3.23] | 0.248 0.296 | 0.902 (0.827, 0.939) 0.856 (0.752, 0.909) | 0.414 |  | 0.982 |  | -0.30 | 0.394 |  | — |  | -0.17 0.04 | 0.112 0.755 |  |
| N3 - Peak Freq | | | | | | | | | | | | | | | | | |
|  | Alpha | NT1: NT2: | 8.61 [8.18, 9.18] 8.40 [8.13, 9.03] | 0.162 0.109 | 0.922 (0.863, 0.952) 0.899 (0.826, 0.936) | 0.423 |  | 0.952 |  | 0.09 | 0.779 |  | — |  | -0.16 0.17 | 0.089 0.103 |  |
|  | Beta |  | 16.3 [16.1, 16.5] 16.3 [16.1, 16.4] | 0.119 0.192 | 0.675 (0.427, 0.798) 0.340 (-0.135, 0.585) | 0.165 |  | 0.810 |  | -0.28 | 0.350 |  | — |  | -0.01 0.57 | 0.949 **0.023** | 0.607 |
|  | Delta |  | 1.03 [0.990, 1.08] 0.985 [0.966, 1.04] | 0.0408 0.0191 | 0.726 (0.519, 0.830) 0.615 (0.325, 0.759) | 0.608 |  | 0.704 |  | 0.38 | 0.257 |  | — |  | -0.19 0.13 | 0.315 0.507 |  |
|  | Gamma |  | 30.3 [30.2, 30.4] 30.3 [30.2, 30.7] | 0.0676 0.0860 | 0.873 (0.777, 0.921) 0.889 (0.807, 0.930) | 0.971 |  | 0.688 |  | -0.20 | 0.563 |  | — |  | 0.05 -0.05 | 0.674 0.624 |  |
|  | Sigma |  | 12.5 [12.3, 12.7] 12.7 [12.4, 13.1] | 0.240 0.360 | 0.724 (0.521, 0.826) 0.661 (0.417, 0.786) | 0.323 |  | 0.443 |  | -0.75 | **0.010** | 0.266 | ✗ |  | 0.01 0.17 | 0.948 0.377 |  |
|  | Theta |  | 4.07 [4.02, 4.21] 4.08 [4.03, 4.39] | 0.0336 0.0961 | 0.789 (0.630, 0.868) 0.750 (0.567, 0.843) | 0.771 |  | 0.810 |  | -0.21 | 0.525 |  | — |  | 0.04 0.07 | 0.807 0.675 |  |
| REM - Baseline | | | | | | | | | | | | | | | | | |
|  | Alpha | NT1: NT2: | 0.657 [0.463, 0.749] 0.787 [0.531, 1.25] | 0.100 0.129 | 0.921 (0.861, 0.951) 0.870 (0.779, 0.917) | 0.448 |  | 0.957 |  | 0.03 | 0.924 |  | — |  | 0.01 0.09 | 0.934 0.429 |  |
|  | Beta |  | 0.100 [0.0472, 0.165] 0.124 [0.0887, 0.142] | 0.0133 0.0205 | 0.911 (0.844, 0.945) 0.823 (0.695, 0.888) | 0.122 |  | 0.936 |  | -0.29 | 0.393 |  | — |  | 0.05 0.05 | 0.633 0.684 |  |
|  | Delta |  | 3.91 [2.99, 4.97] 4.26 [3.14, 6.94] | 0.791 0.955 | 0.801 (0.659, 0.874) 0.690 (0.473, 0.803) | 0.224 |  | 0.840 |  | -0.53 | 0.093 |  | — |  | -0.06 -0.07 | 0.702 0.707 |  |
|  | Gamma |  | 0.0223 [0.00961, 0.0290] 0.0280 [0.0141, 0.0379] | 0.00265 0.00352 | 0.865 (0.763, 0.916) 0.833 (0.708, 0.895) | 0.544 |  | 0.975 |  | -0.53 | 0.135 |  | — |  | 0.09 -0.20 | 0.483 0.125 |  |
|  | Sigma |  | 0.265 [0.216, 0.390] 0.339 [0.257, 0.561] | 0.0447 0.0758 | 0.903 (0.832, 0.939) 0.864 (0.767, 0.914) | 0.255 |  | 0.955 |  | 0.09 | 0.785 |  | — |  | 0.15 -0.01 | 0.162 0.962 |  |
|  | Theta |  | 1.34 [0.956, 1.87] 1.62 [1.11, 2.09] | 0.145 0.267 | 0.893 (0.814, 0.933) 0.851 (0.747, 0.905) | 0.383 |  | 0.806 |  | -0.19 | 0.566 |  | — |  | -0.01 -0.06 | 0.906 0.645 |  |
| REM - Mean | | | | | | | | | | | | | | | | | |
|  | Alpha | NT1: NT2: | 0.117 [0.0996, 0.147] 0.103 [0.0863, 0.120] | 0.0149 0.0198 | 0.751 (0.564, 0.845) 0.838 (0.726, 0.896) | 0.522 |  | 0.813 |  | 0.55 | 0.079 |  | — |  | 0.23 0.25 | 0.169 **0.040** | 0.607 |
|  | Beta |  | 0.0723 [0.0587, 0.0980] 0.0775 [0.0504, 0.0953] | 0.0114 0.0127 | 0.850 (0.742, 0.905) 0.440 (0.045, 0.644) | **0.037** | 0.132 | 0.930 |  | 0.27 | 0.369 |  | — |  | 0.16 -0.06 | 0.211 0.800 |  |
|  | Delta |  | 0.543 [0.430, 0.609] 0.545 [0.483, 0.611] | 0.0486 0.0463 | 0.846 (0.731, 0.904) 0.711 (0.511, 0.815) | 0.091 |  | 0.804 |  | -0.37 | 0.242 |  | — |  | -0.03 -0.25 | 0.846 0.119 |  |
|  | Gamma |  | 0.00477 [0.00316, 0.00712] 0.00557 [0.00364, 0.0107] | 0.000747 0.00101 | 0.899 (0.823, 0.937) 0.754 (0.585, 0.843) | 0.146 |  | 0.868 |  | -0.40 | 0.229 |  | — |  | 0.08 -0.18 | 0.489 0.226 |  |
|  | Sigma |  | 0.0502 [0.0368, 0.0562] 0.0391 [0.0332, 0.0569] | 0.00767 0.0133 | 0.838 (0.718, 0.898) 0.646 (0.406, 0.773) | **0.030** | 0.119 | 0.974 |  | 0.47 | 0.115 |  | — |  | 0.30 0.11 | **0.022** 0.531 | 0.596 |
|  | Theta |  | 0.214 [0.165, 0.234] 0.198 [0.155, 0.234] | 0.0237 0.0113 | 0.801 (0.656, 0.875) 0.708 (0.502, 0.814) | 0.515 |  | 0.906 |  | 0.22 | 0.488 |  | — |  | 0.04 0.31 | 0.788 0.066 |  |
| REM - Peak Amp | | | | | | | | | | | | | | | | | |
|  | Alpha | NT1: NT2: | 2.35 [2.02, 2.69] 2.40 [1.67, 3.79] | 0.167 0.223 | 0.943 (0.902, 0.964) 0.928 (0.876, 0.954) | 0.705 |  | 0.930 |  | 0.13 | 0.701 |  | — |  | -0.06 0.06 | 0.484 0.522 |  |
|  | Beta |  | 3.35 [2.86, 5.35] 3.26 [2.60, 4.40] | 0.166 0.419 | 0.887 (0.801, 0.930) 0.884 (0.803, 0.926) | 0.345 |  | 0.986 |  | 0.12 | 0.747 |  | — |  | 0.03 0.12 | 0.792 0.261 |  |
|  | Delta |  | 2.77 [1.99, 4.16] 2.75 [2.30, 4.82] | 0.985 0.674 | 0.736 (0.537, 0.836) 0.262 (-0.265, 0.532) | 0.071 |  | 0.936 |  | -0.38 | 0.157 |  | — |  | -0.01 -0.22 | 0.943 0.421 |  |
|  | Gamma |  | 2.73 [2.58, 3.31] 2.83 [2.58, 3.44] | 0.178 0.149 | 0.906 (0.836, 0.941) 0.808 (0.673, 0.878) | 0.480 |  | 0.943 |  | 0.18 | 0.594 |  | — |  | -0.18 0.21 | 0.074 0.114 |  |
|  | Sigma |  | 1.58 [1.45, 1.85] 1.76 [1.59, 2.15] | 0.0625 0.0840 | 0.936 (0.887, 0.960) 0.899 (0.826, 0.936) | 0.196 |  | 0.898 |  | -0.46 | 0.187 |  | — |  | 0.16 0.07 | 0.055 0.519 |  |
|  | Theta |  | 1.84 [1.72, 2.05] 2.10 [1.95, 2.28] | 0.135 0.136 | 0.838 (0.714, 0.899) 0.758 (0.587, 0.846) | 0.535 |  | 0.907 |  | -0.81 | **0.011** | 0.266 | ✓ |  | -0.03 -0.05 | 0.852 0.763 |  |
| REM - Peak Freq | | | | | | | | | | | | | | | | | |
|  | Alpha | NT1: NT2: | 8.34 [8.15, 8.86] 8.63 [8.30, 8.99] | 0.129 0.121 | 0.924 (0.868, 0.952) 0.786 (0.639, 0.863) | **0.015** | 0.083 | 0.983 |  | -0.33 | 0.313 |  | — |  | 0.04 0.09 | 0.639 0.510 |  |
|  | Beta |  | 17.5 [17.0, 18.7] 17.6 [17.1, 19.9] | 0.147 0.512 | 0.842 (0.728, 0.900) 0.861 (0.762, 0.911) | 0.868 |  | 0.948 |  | -0.35 | 0.296 |  | — |  | -0.12 -0.09 | 0.351 0.443 |  |
|  | Delta |  | 1.04 [0.978, 1.25] 1.12 [0.945, 1.28] | 0.0848 0.187 | 0.695 (0.455, 0.812) 0.626 (0.365, 0.761) | 0.938 |  | 0.879 |  | -0.39 | 0.213 |  | — |  | 0.01 0.11 | 0.977 0.576 |  |
|  | Gamma |  | 30.3 [30.1, 30.4] 30.3 [30.2, 30.5] | 0.0423 0.103 | 0.903 (0.828, 0.940) 0.843 (0.733, 0.900) | **0.012** | 0.077 | 0.744 |  | -0.61 | 0.069 |  | — |  | -0.03 0.13 | 0.791 0.316 |  |
|  | Sigma |  | 12.7 [12.5, 13.0] 12.9 [12.3, 13.0] | 0.0746 0.188 | 0.899 (0.824, 0.936) 0.846 (0.742, 0.901) | 0.275 |  | 0.803 |  | -0.09 | 0.782 |  | — |  | -0.17 -0.01 | 0.111 0.936 |  |
|  | Theta |  | 4.59 [4.34, 5.53] 4.69 [4.23, 5.06] | 0.275 0.157 | 0.760 (0.588, 0.848) 0.931 (0.882, 0.956) | **0.004** | 0.052 | 0.972 |  | 0.58 | 0.075 |  | — |  | 0.28 0.01 | 0.084 0.909 |  |
| Wake - Baseline | | | | | | | | | | | | | | | | | |
|  | Alpha | NT1: NT2: | 1.79 [1.39, 3.01] 2.17 [1.39, 2.71] | 0.582 0.345 | 0.801 (0.655, 0.875) 0.741 (0.570, 0.832) | 0.337 |  | 0.824 |  | 0.22 | 0.494 |  | — |  | 0.03 0.27 | 0.858 0.073 |  |
|  | Beta |  | 0.714 [0.566, 1.37] 0.375 [0.204, 0.868] | 0.436 0.355 | 0.727 (0.526, 0.829) 0.227 (-0.272, 0.499) | **0.023** | 0.107 | 0.757 |  | 0.17 | 0.537 |  | — |  | -0.06 0.42 | 0.737 0.104 |  |
|  | Delta |  | 8.46 [4.51, 12.8] 5.46 [3.31, 6.26] | 4.43 1.73 | 0.436 (0.014, 0.649) 0.510 (0.183, 0.688) | 0.873 |  | 0.920 |  | 0.28 | 0.277 |  | — |  | -0.16 0.24 | 0.548 0.279 |  |
|  | Gamma |  | 0.312 [0.147, 0.550] 0.147 [0.0410, 0.361] | 0.190 0.176 | 0.660 (0.416, 0.785) 0.561 (0.265, 0.717) | 0.333 |  | 0.866 |  | 0.21 | 0.465 |  | — |  | 0.06 0.29 | 0.773 0.139 |  |
|  | Sigma |  | 1.24 [0.857, 1.80] 0.784 [0.608, 1.32] | 0.352 0.337 | 0.826 (0.695, 0.892) 0.208 (-0.285, 0.492) | **0.020** | 0.099 | 0.754 |  | 0.46 | 0.117 |  | — |  | 0.17 0.50 | 0.235 0.064 |  |
|  | Theta |  | 2.66 [1.39, 3.07] 1.66 [1.20, 2.18] | 0.798 0.347 | 0.718 (0.506, 0.824) 0.615 (0.351, 0.754) | 0.587 |  | 0.705 |  | 0.57 | 0.063 |  | — |  | 0.01 0.22 | 0.961 0.256 |  |
| Wake - Mean | | | | | | | | | | | | | | | | | |
|  | Alpha | NT1: NT2: | 0.117 [0.0802, 0.206] 0.165 [0.118, 0.204] | 0.0481 0.0447 | 0.822 (0.687, 0.889) 0.620 (0.372, 0.753) | **0.011** | 0.077 | 0.801 |  | 0.29 | 0.341 |  | — |  | 0.16 -0.05 | 0.278 0.799 |  |
|  | Beta |  | 0.111 [0.106, 0.138] 0.108 [0.0870, 0.134] | 0.0265 0.0263 | 0.326 (-0.148, 0.581) 0.447 (0.071, 0.644) | 0.653 |  | 0.687 |  | -0.08 | 0.779 |  | — |  | 0.15 0.48 | 0.596 **0.032** | 0.607 |
|  | Delta |  | 0.506 [0.411, 0.646] 0.499 [0.441, 0.558] | 0.0931 0.104 | 0.630 (0.360, 0.768) 0.554 (0.261, 0.711) | 0.532 |  | 0.898 |  | -0.44 | 0.085 |  | — |  | -0.25 0.04 | 0.228 0.847 |  |
|  | Gamma |  | 0.0146 [0.00989, 0.0207] 0.0108 [0.00772, 0.0152] | 0.00632 0.00365 | 0.693 (0.457, 0.811) 0.646 (0.411, 0.771) | 0.588 |  | 0.775 |  | -0.16 | 0.602 |  | — |  | -0.01 0.36 | 0.951 **0.039** | 0.607 |
|  | Sigma |  | 0.0537 [0.0436, 0.0684] 0.0536 [0.0509, 0.0731] | 0.00971 0.0120 | 0.576 (0.259, 0.734) 0.679 (0.460, 0.794) | 0.741 |  | 0.842 |  | -0.14 | 0.628 |  | — |  | 0.45 0.29 | **0.032** 0.087 | 0.596 |
|  | Theta |  | 0.116 [0.108, 0.152] 0.131 [0.0963, 0.157] | 0.0141 0.0263 | 0.842 (0.725, 0.901) 0.574 (0.282, 0.727) | **0.029** | 0.119 | 0.924 |  | 0.40 | 0.207 |  | — |  | 0.02 -0.15 | 0.893 0.463 |  |
| Wake - Peak Amp | | | | | | | | | | | | | | | | | |
|  | Alpha | NT1: NT2: | 2.19 [1.69, 2.74] 2.16 [1.82, 2.96] | 0.296 0.362 | 0.776 (0.606, 0.861) 0.662 (0.434, 0.782) | 0.138 |  | 0.828 |  | 0.32 | 0.342 |  | — |  | 0.01 -0.18 | 0.935 0.313 |  |
|  | Beta |  | 2.43 [2.12, 3.41] 3.05 [2.12, 3.54] | 0.436 0.510 | 0.723 (0.522, 0.825) 0.695 (0.497, 0.802) | 0.482 |  | 0.954 |  | 0.04 | 0.899 |  | — |  | -0.01 0.03 | 0.973 0.879 |  |
|  | Delta |  | 16.9 [10.4, 22.3] 10.6 [5.47, 18.1] | 5.32 6.73 | 0.682 (0.449, 0.801) 0.387 (-0.023, 0.605) | **0.027** | 0.118 | 0.933 |  | 0.10 | 0.722 |  | — |  | -0.19 0.19 | 0.334 0.423 |  |
|  | Gamma |  | 2.43 [2.17, 2.52] 2.49 [2.16, 2.74] | 0.264 0.521 | 0.650 (0.391, 0.780) 0.137 (-0.449, 0.447) | **0.000** | **0.035** | 0.868 |  | 0.06 | 0.811 |  | — |  | -0.21 -0.23 | 0.271 0.417 |  |
|  | Sigma |  | 1.61 [1.47, 1.73] 1.94 [1.59, 2.30] | 0.115 0.285 | 0.631 (0.358, 0.770) 0.257 (-0.264, 0.526) | 0.078 |  | 0.941 |  | -0.63 | **0.027** | 0.318 | ✓ |  | 0.10 -0.34 | 0.616 0.216 |  |
|  | Theta |  | 2.14 [1.95, 2.87] 2.21 [2.04, 2.47] | 0.449 0.447 | 0.515 (0.163, 0.694) 0.562 (0.260, 0.720) | 0.451 |  | 0.512 |  | 0.46 | 0.120 |  | — |  | -0.19 -0.30 | 0.408 0.148 |  |
| Wake - Peak Freq | | | | | | | | | | | | | | | | | |
|  | Alpha | NT1: NT2: | 9.12 [8.75, 9.36] 9.40 [8.84, 9.90] | 0.131 0.230 | 0.885 (0.800, 0.928) 0.597 (0.324, 0.741) | **0.001** | **0.035** | 0.933 |  | -0.54 | 0.079 |  | — |  | 0.16 0.31 | 0.168 0.116 |  |
|  | Beta |  | 20.0 [18.9, 20.9] 19.6 [18.2, 20.2] | 0.768 0.772 | 0.792 (0.638, 0.869) 0.689 (0.487, 0.798) | 0.126 |  | 0.922 |  | -0.12 | 0.710 |  | — |  | -0.07 0.16 | 0.627 0.345 |  |
|  | Delta |  | 0.827 [0.812, 0.919] 0.873 [0.835, 0.967] | 0.0446 0.0613 | 0.882 (0.796, 0.926) 0.805 (0.677, 0.874) | 0.064 |  | 0.959 |  | 0.00 | 0.990 |  | — |  | -0.09 -0.12 | 0.473 0.379 |  |
|  | Gamma |  | 30.5 [30.4, 30.6] 30.4 [30.3, 30.6] | 0.113 0.0768 | 0.426 (0.012, 0.637) 0.446 (0.080, 0.641) | 0.812 |  | 0.793 |  | -0.09 | 0.749 |  | — |  | -0.20 0.39 | 0.431 0.080 |  |
|  | Sigma |  | 13.4 [12.7, 13.6] 12.9 [12.4, 13.4] | 0.246 0.286 | 0.940 (0.895, 0.963) 0.887 (0.809, 0.928) | **0.013** | 0.080 | 0.891 |  | 0.37 | 0.255 |  | — |  | -0.21 0.03 | **0.007** 0.761 | 0.433 |
|  | Theta |  | 5.01 [4.45, 5.95] 5.33 [4.63, 6.47] | 0.428 0.252 | 0.887 (0.802, 0.930) 0.743 (0.573, 0.834) | **0.036** | 0.132 | 0.932 |  | 0.48 | 0.098 |  | — |  | 0.06 0.08 | 0.623 0.613 |  |
| *Statistics*: Median and IQR of observations at baseline, Δ indicates median absolute difference between observations at baseline and week 4. *Reliability*: Estimated reliability with 95% confidence intervals; *p*, the *p*-value of Levene's test; Val., the estimated reliability in the validation cohort. *Diagnosis effect*: Effect size (NT2 versus NT1) in standardized units; *p*, the *p*-value of a t-test; Val., a check (✓) indicates the diagnosis effect is significant in the validation cohort, an ex (✗) indicates the diagnosis effect is not significant in the validation cohort, a dash (—) indicates validation of the diagnosis effect was not tested. *Study visit effect*: Comparison of values at baseline and while "on-placebo"; *p*, the *p*-value of a t-test; *FDR*: The false discovery rate (FDR) adjusted *p*-value. For each feature, results for NT1 are shown on the first line and NT2 are shown in the second line. | | | | | | | | | | | | | | | | | |

| **Spindle features** | | | | | | | | | | | | | | | | | |
| --- | --- | --- | --- | --- | --- | --- | --- | --- | --- | --- | --- | --- | --- | --- | --- | --- | --- |
|  | **Feature** |  | **Statistics** | | **Reliability** | | | |  | **Diagnosis effect** | | | |  | **Study visit effect** | | |
|  |  |  | Median [IQR] | Δ | Est. (95% CI) | *p* | FDR | Val. |  | NT1 eff. (SD) | *p* | FDR | Val. |  | Est. | *p* | FDR |
| N2 - HighF | | | | | | | | | | | | | | | | | |
|  | Density, /min | NT1: NT2: | 2.24 [1.74, 2.75] 1.42 [0.614, 2.45] | 0.606 0.440 | 0.819 (0.681, 0.887) 0.913 (0.856, 0.944) | 0.051 |  | 0.809 |  | 0.37 | 0.256 |  | — |  | 0.18 0.15 | 0.237 0.103 |  |
|  | Duration, s |  | 0.955 [0.883, 1.02] 0.921 [0.799, 0.968] | 0.0538 0.0361 | 0.805 (0.656, 0.878) 0.846 (0.744, 0.900) | 0.406 |  | 0.818 |  | 0.19 | 0.550 |  | — |  | -0.16 0.00 | 0.303 0.989 |  |
|  | Frequency, Hz |  | 13.1 [13.0, 13.4] 13.4 [13.0, 13.5] | 0.114 0.0921 | 0.811 (0.676, 0.881) 0.782 (0.637, 0.859) | 0.892 |  | 0.711 |  | -0.25 | 0.434 |  | — |  | -0.17 0.13 | 0.242 0.354 |  |
|  | RMS, |  | 8.74 [7.68, 11.0] 7.65 [6.85, 10.9] | 0.679 0.582 | 0.916 (0.854, 0.947) 0.951 (0.918, 0.969) | 0.063 |  | 0.871 |  | 0.22 | 0.508 |  | — |  | 0.04 0.10 | 0.662 0.146 |  |
|  | RelPower, Pr |  | 0.477 [0.460, 0.507] 0.498 [0.428, 0.531] | 0.0222 0.0332 | 0.751 (0.568, 0.843) 0.858 (0.762, 0.909) | 0.069 |  | 0.636 |  | -0.50 | 0.116 |  | — |  | -0.13 0.11 | 0.436 0.351 |  |
| N2 - LowF | | | | | | | | | | | | | | | | | |
|  | Density, /min | NT1: NT2: | 1.99 [1.54, 2.42] 1.50 [1.27, 1.94] | 0.416 0.247 | 0.601 (0.305, 0.750) 0.947 (0.912, 0.966) | **0.000** | **0.008** | 0.936 |  | 1.04 | **0.001** | **0.014** | ✗ |  | 0.37 0.10 | 0.082 0.163 |  |
|  | Duration, s |  | 1.01 [0.983, 1.03] 1.00 [0.986, 1.01] | 0.0371 0.0191 | 0.340 (-0.146, 0.589) 0.709 (0.516, 0.812) | 0.118 |  | 0.0326 |  | 0.03 | 0.914 |  | — |  | -0.46 0.03 | 0.080 0.864 |  |
|  | Frequency, Hz |  | 10.5 [10.2, 10.8] 10.1 [9.91, 10.5] | 0.0899 0.0648 | 0.944 (0.903, 0.965) 0.929 (0.883, 0.954) | 0.668 |  | 0.888 |  | 0.37 | 0.252 |  | — |  | 0.02 -0.03 | 0.810 0.727 |  |
|  | RMS, |  | 9.31 [8.18, 11.2] 9.11 [8.60, 12.2] | 0.967 0.718 | 0.904 (0.833, 0.940) 0.956 (0.927, 0.972) | **0.010** | 0.069 | 0.937 |  | 0.22 | 0.499 |  | — |  | 0.04 0.06 | 0.723 0.375 |  |
|  | RelPower, Pr |  | 0.449 [0.433, 0.470] 0.442 [0.419, 0.446] | 0.00965 0.0104 | 0.831 (0.705, 0.894) 0.874 (0.792, 0.918) | 0.551 |  | 0.884 |  | 0.35 | 0.296 |  | — |  | -0.01 0.07 | 0.963 0.504 |  |
| N3 - HighF | | | | | | | | | | | | | | | | | |
|  | Density, /min | NT1: NT2: | 0.962 [0.378, 1.49] 0.417 [0.240, 0.604] | 0.416 0.253 | 0.787 (0.622, 0.869) 0.931 (0.873, 0.959) | **0.017** | 0.072 | 0.735 |  | 0.61 | 0.093 |  | — |  | 0.23 0.14 | 0.151 0.139 |  |
|  | Duration, s |  | 0.864 [0.770, 0.892] 0.780 [0.750, 0.800] | 0.0299 0.0653 | 0.741 (0.545, 0.839) 0.483 (0.080, 0.681) | **0.021** | 0.072 | 0.439 |  | 0.24 | 0.441 |  | — |  | -0.18 -0.13 | 0.285 0.597 |  |
|  | Frequency, Hz |  | 12.9 [12.8, 13.3] 13.2 [12.7, 13.4] | 0.0732 0.238 | 0.828 (0.698, 0.893) 0.841 (0.711, 0.904) | 0.792 |  | 0.609 |  | -0.59 | 0.088 |  | — |  | -0.17 0.02 | 0.220 0.920 |  |
|  | RMS, |  | 9.22 [7.60, 10.7] 9.47 [7.09, 12.9] | 0.712 0.693 | 0.817 (0.681, 0.885) 0.921 (0.858, 0.952) | **0.022** | 0.072 | 0.840 |  | 0.14 | 0.662 |  | — |  | 0.20 -0.03 | 0.164 0.783 |  |
|  | RelPower, Pr |  | 0.418 [0.405, 0.452] 0.462 [0.401, 0.505] | 0.0282 0.0718 | 0.743 (0.554, 0.839) 0.515 (0.131, 0.703) | 0.073 |  | 0.316 |  | -0.84 | **0.006** | 0.082 | ✗ |  | -0.23 -0.10 | 0.170 0.701 |  |
|  | SW amplitude, µV |  | 29.9 [23.4, 36.6] 34.9 [28.4, 40.8] | 4.28 7.90 | 0.622 (0.336, 0.765) 0.344 (-0.175, 0.598) | 0.074 |  | 0.345 |  | -0.44 | 0.144 |  | — |  | 0.09 -0.03 | 0.679 0.909 |  |
|  | SW coupling, % |  | 68.4 [56.9, 79.1] 66.7 [57.7, 86.7] | 10.6 15.5 | 0.341 (-0.093, 0.596) 0.029 (-0.697, 0.397) | 0.262 |  | 0.0655 |  | -0.25 | 0.348 |  | — |  | -0.08 -0.05 | 0.787 0.892 |  |
|  | SW(z) amplitude, z-score |  | 4.21 [4.02, 4.58] 4.55 [3.79, 5.58] | 0.386 1.23 | 0.454 (0.034, 0.661) 0.093 (-0.602, 0.441) | 0.162 |  | 0.326 |  | -0.41 | 0.132 |  | — |  | 0.19 0.28 | 0.452 0.409 |  |
| N3 - LowF | | | | | | | | | | | | | | | | | |
|  | Density, /min | NT1: NT2: | 2.41 [1.17, 2.77] 1.19 [0.617, 2.42] | 0.729 0.502 | 0.593 (0.294, 0.744) 0.901 (0.829, 0.937) | **0.003** | **0.038** | 0.979 |  | 0.69 | **0.026** | 0.222 | ✗ |  | 0.23 0.17 | 0.282 0.099 |  |
|  | Duration, s |  | 0.994 [0.935, 1.02] 1.01 [0.985, 1.05] | 0.0355 0.0699 | 0.633 (0.357, 0.770) 0.019 (-0.587, 0.381) | **0.036** | 0.104 | 0.314 |  | -0.06 | 0.808 |  | — |  | -0.28 -0.37 | 0.166 0.242 |  |
|  | Frequency, Hz |  | 10.4 [10.1, 10.7] 10.1 [9.77, 10.6] | 0.192 0.151 | 0.919 (0.857, 0.949) 0.907 (0.839, 0.941) | 0.662 |  | 0.956 |  | 0.43 | 0.214 |  | — |  | 0.05 -0.01 | 0.608 0.916 |  |
|  | RMS, |  | 9.21 [8.08, 11.1] 8.56 [8.07, 11.2] | 0.674 0.717 | 0.885 (0.801, 0.928) 0.929 (0.877, 0.956) | 0.202 |  | 0.847 |  | 0.19 | 0.562 |  | — |  | 0.08 0.12 | 0.505 0.187 |  |
|  | RelPower, Pr |  | 0.445 [0.399, 0.471] 0.439 [0.406, 0.478] | 0.0204 0.0253 | 0.848 (0.732, 0.906) 0.821 (0.695, 0.886) | 0.637 |  | 0.716 |  | 0.05 | 0.877 |  | — |  | -0.00 -0.09 | 0.997 0.491 |  |
|  | SW amplitude, µV |  | 36.0 [26.3, 37.1] 32.8 [27.1, 39.8] | 4.41 6.75 | 0.769 (0.600, 0.855) 0.416 (-0.003, 0.630) | **0.022** | 0.072 | 0.870 |  | -0.25 | 0.410 |  | — |  | -0.14 -0.20 | 0.380 0.395 |  |
|  | SW coupling, % |  | 70.9 [59.6, 74.4] 77.0 [67.8, 84.7] | 8.77 10.6 | 0.713 (0.503, 0.820) 0.287 (-0.205, 0.545) | **0.011** | 0.069 | 0.603 |  | -0.16 | 0.593 |  | — |  | 0.32 -0.15 | 0.069 0.565 |  |
|  | SW(z) amplitude, z-score |  | 4.43 [4.10, 5.36] 4.81 [4.20, 5.82] | 0.374 1.01 | 0.743 (0.541, 0.843) 0.573 (0.267, 0.730) | 0.200 |  | 0.248 |  | -0.42 | 0.194 |  | — |  | -0.29 -0.10 | 0.097 0.634 |  |
| *Statistics*: Median and IQR of observations at baseline, Δ indicates median absolute difference between observations at baseline and week 4. *Reliability*: Estimated reliability with 95% confidence intervals; *p*, the *p*-value of Levene's test; Val., the estimated reliability in the validation cohort. *Diagnosis effect*: Effect size (NT2 versus NT1) in standardized units; *p*, the *p*-value of a t-test; Val., a check (✓) indicates the diagnosis effect is significant in the validation cohort, an ex (✗) indicates the diagnosis effect is not significant in the validation cohort, a dash (—) indicates validation of the diagnosis effect was not tested. *Study visit effect*: Comparison of values at baseline and while "on-placebo"; *p*, the *p*-value of a t-test; *FDR*: The false discovery rate (FDR) adjusted *p*-value. For each feature, results for NT1 are shown on the first line and NT2 are shown in the second line. | | | | | | | | | | | | | | | | | |

| **Periodic limb movements (PLMs) and eye movements (EMs)** | | | | | | | | | | | | | | | | | |
| --- | --- | --- | --- | --- | --- | --- | --- | --- | --- | --- | --- | --- | --- | --- | --- | --- | --- |
|  | **Feature** |  | **Statistics** | | **Reliability** | | | |  | **Diagnosis effect** | | | |  | **Study visit effect** | | |
|  |  |  | Median [IQR] | Δ | Est. (95% CI) | *p* | FDR | Val. |  | NT1 eff. (SD) | *p* | FDR | Val. |  | Est. | *p* | FDR |
|  | PLMI, /Hr | NT1: NT2: | 23.6 [0.00, 45.6] 0.00 [0.00, 18.5] | 33.8 0.00 | 0.817 (0.674, 0.887) 0.873 (0.789, 0.918) | 0.077 |  | NA*^1^* |  | 0.52 | 0.116 |  | — |  | -0.04 0.06 | 0.772 0.584 |  |
|  | PLM Bouts, N |  | 1.50 [0.00, 2.75] 0.00 [0.00, 1.00] | 2.00 0.00 | 0.824 (0.687, 0.891) 0.858 (0.764, 0.908) | 0.132 |  | NA*^1^* |  | 0.50 | 0.130 |  | — |  | -0.07 0.08 | 0.647 0.506 |  |
|  | EM Density, /min |  | 2.88 [1.43, 4.90] 1.86 [0.818, 3.76] | 0.917 0.452 | 0.790 (0.639, 0.867) 0.849 (0.749, 0.902) | **0.028** | 0.057 | 0.674 |  | 0.79 | **0.012** | **0.047** | ✓ |  | 0.03 0.01 | 0.861 0.944 |  |
|  | EM Duration, ms |  | 742 [724, 762] 735 [721, 744] | 17.9 19.0 | 0.881 (0.792, 0.925) 0.657 (0.428, 0.779) | **0.005** | **0.018** | 0.962 |  | 0.23 | 0.486 |  | — |  | -0.22 -0.10 | **0.049** 0.597 | 0.196 |
| *Statistics*: Median and IQR of observations at baseline, Δ indicates median absolute difference between observations at baseline and week 4. *Reliability*: Estimated reliability with 95% confidence intervals; *p*, the *p*-value of Levene's test; Val., the estimated reliability in the validation cohort. *Diagnosis effect*: Effect size (NT2 versus NT1) in standardized units; *p*, the *p*-value of a t-test; Val., a check (✓) indicates the diagnosis effect is significant in the validation cohort, an ex (✗) indicates the diagnosis effect is not significant in the validation cohort, a dash (—) indicates validation of the diagnosis effect was not tested. *Study visit effect*: Comparison of values at baseline and while "on-placebo"; *p*, the *p*-value of a t-test; *FDR*: The false discovery rate (FDR) adjusted *p*-value. For each feature, results for NT1 are shown on the first line and NT2 are shown in the second line. *^1^* PLMS were not annotated in the validation cohort. | | | | | | | | | | | | | | | | | |

| **MWT sleep onset latencies** | | | | | | | | | | | | | | | | | |
| --- | --- | --- | --- | --- | --- | --- | --- | --- | --- | --- | --- | --- | --- | --- | --- | --- | --- |
|  | **Test time** |  | **Statistics** | | **Reliability** | | | |  | **Diagnosis effect** | | | |  | **Study visit effect** | | |
|  |  |  | Median [IQR] | Δ | Est. (95% CI) | *p* | FDR | Val. |  | NT1 eff. (SD) | *p* | FDR | Val. |  | Est. | *p* | FDR |
|  | 10 am, min | NT1: NT2: | 2.50 [1.00, 5.50] 6.00 [2.75, 14.8] | 1.00 4.50 | 0.796 (0.652, 0.870) 0.248 (-0.223, 0.505) | **0.000** | **0.000** | 0.00 |  | -0.61 | **0.010** | **0.013** | ✗ |  | -0.10 -0.42 | 0.469 0.082 |  |
|  | 12 pm, min |  | 1.50 [0.500, 5.00] 5.50 [2.00, 10.2] | 2.00 5.00 | 0.609 (0.340, 0.750) 0.487 (0.161, 0.664) | 0.074 |  | 0.874 |  | -0.59 | **0.026** | **0.026** | ✓ |  | -0.10 0.33 | 0.601 0.106 |  |
|  | 2 pm, min |  | 2.50 [0.500, 5.00] 5.00 [4.25, 8.75] | 1.50 3.00 | 0.823 (0.693, 0.888) 0.325 (-0.115, 0.559) | **0.000** | **0.000** | 0.800 |  | -0.83 | **0.003** | **0.004** | ✓ |  | -0.10 0.30 | 0.460 0.206 |  |
|  | 4 pm, min |  | 2.00 [0.500, 5.00] 6.00 [3.00, 19.0] | 1.50 4.50 | 0.887 (0.805, 0.929) 0.288 (-0.174, 0.535) | **0.000** | **0.000** | 0.656 |  | -0.99 | **0.001** | **0.003** | ✓ |  | 0.02 0.25 | 0.822 0.306 |  |
|  | Mean, min |  | 2.00 [1.12, 6.38] 7.50 [4.25, 14.7] | 0.875 5.12 | 0.869 (0.777, 0.917) 0.570 (0.300, 0.717) | **0.000** | **0.000** | 0.483 |  | -0.85 | **0.001** | **0.003** | ✓ |  | -0.02 0.12 | 0.833 0.516 |  |
| *Statistics*: Median and IQR of observations at baseline, Δ indicates median absolute difference between observations at baseline and week 4. *Reliability*: Estimated reliability with 95% confidence intervals; *p*, the *p*-value of Levene's test; Val., the estimated reliability in the validation cohort. *Diagnosis effect*: Effect size (NT2 versus NT1) in standardized units; *p*, the *p*-value of a t-test; Val., a check (✓) indicates the diagnosis effect is significant in the validation cohort, an ex (✗) indicates the diagnosis effect is not significant in the validation cohort, a dash (—) indicates validation of the diagnosis effect was not tested. *Study visit effect*: Comparison of values at baseline and while "on-placebo"; *p*, the *p*-value of a t-test; *FDR*: The false discovery rate (FDR) adjusted *p*-value. For each feature, results for NT1 are shown on the first line and NT2 are shown in the second line. | | | | | | | | | | | | | | | | | |

**Supplementary Table 2.** Comparison of test-retest reliability in NT1 vs NT2 for different features (N=119, Levene’s test, p<0.05).

|  | **Hypnogram (N=8)** | **Hypnodensity (N=58)** | **qEEG (N=38)** | **Spindle (N=9)** | **Other (N=2)** | **MWT (N=4)** |
| --- | --- | --- | --- | --- | --- | --- |
| **NT1 higher** | REM Bout duration (.70 vs .40) %NREM (.71 vs .51) N1→REM (.73 vs .37) N1→Wake (.68 vs .08) Wake→N2 (.71 vs .35) Wake→REM (.53 vs 0) | N2 AT10 (.62 vs .17) N1 AT30 (.69 vs .32) N1•N2 AT30 (.55 vs .09) Wake•N1 AT30 (.69 vs .31) Wake•N1•N2 AT30 (.60 vs .23) Wake•N1•N2•N3 AT30 (.66 vs .30) Wake•N1•N3 AT30 (.68 vs .42) Wake•N2 AT30 (.56 vs .26) **N1 AT50 (.72 vs .35)** N1•N2•REM AT50 (.66 vs .21) N1•REM AT50 (.45 vs 0) **Wake•N1•REM AT50 (.66 vs 0)** Wake•N2•REM AT50 (.59 vs .24) **Wake•N3•REM AT50 (.68 vs .22)** N3•REM AT50(w) (.73 vs .46) N1 Max (.41 vs 0) N1•N2•N3•REM Max (.72 vs .40) **N1•N2•REM Max (.67 vs .17)** **N1•N3•REM Max (.71 vs .31)** Wake Max (.71 vs .25) Wake•N1•N3•REM Max (.61 vs .26) **Wake•N1•REM Max (.78 vs .30)** Wake•N3•REM Max (.73 vs .34) Wake•N1•N2•N3•REM Prevalence (.88 vs .71) N1•N3•REM Entropy (.48 vs .13) Wake•N1•N2 Entropy (.52 vs .04) Wake•N3•REM Entropy (.48 vs .14) Wake•REM Entropy (.49 vs .15) | N2 Alpha, Mean (.83 vs .58) N3 Alpha, Mean (.82 vs .58) Wake Alpha, Mean (.82 vs .62) N1 Beta, Mean (.72 vs .34) N3 Beta, Mean (.79 vs .33) REM Beta, Mean (.85 vs .44) N1 Delta, Mean (.88 vs .63) N2 Delta, Mean (.88 vs .47) N3 Delta, Mean (.82 vs .49) N3 Gamma, Mean (.79 vs .39) N1 Sigma, Mean (.88 vs .61) REM Sigma, Mean (.84 vs .65) N3 Theta, Mean (.88 vs .66) Wake Theta, Mean (.84 vs .57) N2 Alpha, Peak Amp (.97 vs .89) **N1 Delta, Peak Amp (.72 vs 0)** N3 Delta, Peak Amp (.85 vs .54) Wake Delta, Peak Amp (.68 vs .39) **Wake Gamma, Peak Amp (.65 vs .14)** N2 Sigma, Peak Amp (.85 vs .66) N3 Sigma, Peak Amp (.87 vs .68) N1 Theta, Peak Amp (.86 vs .51) N1 Beta, Baseline (.84 vs .39) Wake Beta, Baseline (.73 vs .23) N1 Delta, Baseline (.79 vs .57) N2 Delta, Baseline (.90 vs .78) N2 Gamma, Baseline (.89 vs .16) N3 Gamma, Baseline (.92 vs .67) N2 Sigma, Baseline (.91 vs .64) Wake Sigma, Baseline (.83 vs .21) REM Alpha, Peak Freq (.92 vs .79) **Wake Alpha, Peak Freq (.89 vs .60)** N1 Beta, Peak Freq (.86 vs .52) N2 Beta, Peak Freq (.90 vs .60) REM Gamma, Peak Freq (.90 vs .84) Wake Sigma, Peak Freq (.94 vs .89) Wake Theta, Peak Freq (.89 vs .74) | N3 HighF, Dur (.74 vs .48) N3 LowF, Dur (.63 vs .02) N3 LowF, SW coupling (.71 vs .29) N3 LowF, SW amplitude (.77 vs .42) | **REM EM Duration (.88 vs .66)** | **10 am SOL (.80 vs .25)** **2 pm SOL (.82 vs .32)** **4 pm SOL (.89 vs .29)** **Mean SOL (.87 vs .57)** |
| **NT2 higher** | **N2→N2 (.54 vs .83)** REM→N2 (.22 vs .61) | **N1 AT05 (.25 vs .83)** N1•N2•N3•REM AT05 (.21 vs .63) **N1•N2•REM AT05 (0 vs .57)** **N1•N3•REM AT05 (.01 vs .69)** N2 AT05 (0 vs .69) N2•REM AT05 (.18 vs .70) **REM AT05 (.13 vs .77)** **Wake•N1 AT05 (.12 vs .70)** Wake•N1•N2•N3 AT05 (.32 vs .68) Wake•N1•N2•N3•REM AT05 (.16 vs .69) **Wake•N1•N2•REM AT05 (0 vs .67)** **Wake•N1•N3•REM AT05 (0 vs .66)** **Wake•N1•REM AT05 (0 vs .68)** **Wake•N2•REM AT05 (0 vs .64)** **Wake•N3•REM AT05 (0 vs .61)** **Wake•REM AT05 (0 vs .55)** **N1•N3•REM AT10 (.09 vs .67)** N1•REM AT10 (.22 vs .66) **N2•REM AT10 (.25 vs .76)** N3•REM AT10 (.50 vs .80) **REM AT10 (.23 vs .70)** Wake•N1•REM AT10 (.06 vs .50) Wake•N2•REM AT10 (0 vs .42) Wake•N3•REM AT10 (.02 vs .60) **Wake•REM AT10 (0 vs .57)** Wake•N1 AT50(w) (.73 vs .87) **N2•REM Max (0 vs .82)** REM Max (.48 vs .72) N1•REM Prevalence (.69 vs .84) N1•N3 Entropy (.19 vs .58) | REM Theta, Peak Freq (.76 vs .93) | N3 HighF, Dens (.79 vs .93) **N2 LowF, Dens (.60 vs .95)** **N3 LowF, Dens (.59 vs .90)** N3 HighF, RMS (.82 vs .92) N2 LowF, RMS (.90 vs .96) | REM EM Density (.79 vs .85) |  |

EM, eye movement; N1/N2/N3, sleep stage N1/N2/N3; NT1, narcolepsy type 1; NT2, narcolepsy type 2; qEEG, quantitative electroencephalography; REM, rapid eye movement. Entries are shown as *Feature (estimated reliability in NT1 vs NT2)*. **Bold** entries indicate features with p<0.05 after false discovery rate adjustment.

**Supplementary Table 3.** Study visit effects for hypnodensity, qEEG, spindle, and movement features.

| Feature | Effect size | *p* | False Discovery Rate adjusted p-value |
| --- | --- | --- | --- |
| Study visit effects in NT1 (N=23) | | | |
| Hypnodensity (N=13) | | | |
| Wake•N2 AT10 | −0.387 | 0.043 | 0.838 |
| N1•N2•REM AT50 | −0.401 | 0.038 | 0.838 |
| N1•REM AT50 | −0.563 | 0.016 | 0.838 |
| Wake•N1•N3 AT50(w) | 0.502 | 0.011 | 0.838 |
| Wake•N2 AT50(w) | 0.379 | 0.032 | 0.838 |
| Wake•N3•REM AT50(w) | 0.294 | 0.042 | 0.838 |
| Wake•REM AT50(w) | 0.279 | 0.037 | 0.838 |
| Wake•N2 Prevalence | 0.405 | 0.048 | 0.838 |
| Wake•N2•REM Prevalence | 0.306 | 0.031 | 0.838 |
| N2•N3 Entropy | 0.472 | 0.044 | 0.838 |
| N3 Entropy | 0.514 | 0.045 | 0.838 |
| Wake•N2•N3 Entropy | 0.706 | 0.013 | 0.838 |
| Wake•N3 Entropy | 0.771 | **0.003** | 0.831 |
| qEEG (N=9) | | | |
| N2 Alpha, Mean | 0.279 | 0.045 | 0.603 |
| N3 Alpha, Mean | 0.288 | 0.040 | 0.603 |
| N2 Sigma, Mean | 0.229 | 0.032 | 0.596 |
| REM Sigma, Mean | 0.300 | 0.022 | 0.596 |
| Wake Sigma, Mean | 0.453 | 0.032 | 0.596 |
| N3 Gamma, Peak Amp | 0.309 | 0.035 | 0.596 |
| N1 Sigma, Peak Amp | 0.452 | 0.011 | 0.434 |
| N1 Sigma, Peak Freq | −0.348 | **0.000** | 0.060 |
| Wake Sigma, Peak Freq | −0.210 | **0.007** | 0.433 |
| Other (N=1) | | | |
| REM EM Duration | −0.221 | 0.049 | 0.196 |
| Study effect visits in NT2 (N=13) | | | |
| Hypnogram (N=2) | | | |
| N1→Wake | −0.609 | 0.027 | 0.165 |
| N2→Wake | −0.495 | **0.007** | 0.084 |
| Hypnodensity (N=3) | | | |
| N1 AT10 | −0.407 | 0.038 | 0.980 |
| REM Max | −0.377 | 0.027 | 0.980 |
| N3 Entropy | −0.453 | 0.042 | 0.980 |
| qEEG (N=8) | | | |
| REM Alpha, Mean | 0.246 | 0.040 | 0.607 |
| Wake Beta, Mean | 0.478 | 0.032 | 0.607 |
| Wake Gamma, Mean | 0.362 | 0.039 | 0.607 |
| N3 Gamma, Peak Amp | 0.445 | 0.037 | 0.607 |
| N2 Sigma, Peak Amp | 0.386 | 0.037 | 0.607 |
| N1 Beta, Peak Freq | 0.473 | 0.019 | 0.607 |
| N3 Beta, Peak Freq | 0.575 | 0.023 | 0.607 |
| N1 Theta, Peak Freq | 0.310 | **0.008** | 0.607 |

EM, eye movement; NT1, narcolepsy type 1; NT2, narcolepsy type 2; qEEG, quantitative electroencephalography; REM, rapid eye movement. Comparison of observations while ‘on-placebo’ versus baseline (t-test). Effect sizes are reported as standard deviations.

**Supplementary Figure 1.** qEEG feature reliability estimated from the combined NT1 and NT2 cohorts.

IQR, interquartile range; NT1, narcolepsy type 1; NT2, narcolepsy type 2; qEEG, quantitative electroencephalography.

Reliability in each feature, band and stage in qEEG features. Reliability estimates are based on the combined NT1 and NT2 cohorts. Black lines trace the reliability in the qEEG measure indicated along the left; colored boxes are repeated in each row and gray boxes indicate the median and IQR for the measure and stage in the corresponding panel.

**Supplementary Figure 2.** Spindle feature reliability estimated from the combined NT1 and NT2 cohorts.

Dens, mean density; Dur, mean duration; F, frequency; n2/n3, sleep stage N2/N3; PctCoupled, per cent of spindles couples to slow waves; RelPower, mean relative power; RMS, mean absolute power (measured as signal root mean square); SWAmpZ, mean amplitude (in standardized units) of the slow wave at the spindle peak.

**Supplementary Figure 3.** Comparison of qEEG feature reliability in NT1 versus NT2.

NT1, narcolepsy type 1; NT2, narcolepsy type 2; qEEG, quantitative electroencephalography.

The horizontal axis indicates sleep stage; the vertical axis indicates frequency band; each panel indicates a different measure of qEEG activity; color indicates the difference in reliability between NT1 and NT2 with red indicating higher reliability in NT1. *p<0.05, **p<0.01, ***p<0.001 for Levene’s test for homogeneity of variance.

**Supplementary Figure 4.** Change from baseline in hypnogram stage percentages in each quarter-night.

BSL, baseline; IQR, interquartile range; N1/N2/N3, sleep stage N1/N2/N3; NT1, narcolepsy type 1; NT2, narcolepsy type 2; REM, rapid eye movement; Wk4/8, Week 4/Week 8; wn, whole-night.

Values are shown as changes from baseline in units of standard deviations computed based on transformed observations (see Methods). Boxplots indicate median, first, and third quartiles; whiskers extend no further than the end of the box plus 1.5 IQR. Thin gray lines indicate values observed in each participant. Thick colored bands indicate 95% CI of model-estimated contrasts with baseline: lighter colored bands correspond to models with a three-level visit factor (baseline, Week 4, Week 8), and darker colored bands correspond to models with a two-level visit factor (baseline vs. follow-up). Color indicates the quarter-night or whole-night. °p < 0.1, *p < 0.05, **p < 0.01, ***p < 0.001; p-values for three-level visit factor models are adjusted within each feature and quarter using the Dunnett’s method for two tests.

**Supplementary Figure 5.** MWT SOL in each participant and test.

BSL, baseline; MWT, maintenance of wakefulness test; SOL, sleep onset latency; Wk4/8, Week 4/Week 8.

(A) Values indicate SOL in minutes; color indicates shorter MWT SOL. (B) Values indicate difference from baseline in minutes with color indicating larger absolute differences. Each row corresponds to an individual participant. Participants are ordered by mean SOL at baseline.

**Supplementary Figure 6.** Maintenance of wakefulness test sleep onset latency in the NT1 validation cohort.

BSL, baseline; MWT, Maintenance of Wakefulness Test; NT1, narcolepsy type 1; SOL, sleep onset latency; Wk8, Week 8.

(A) Classification consistency based on a cutoff of 8 minutes for the NT1 validation cohort. The top row shows the proportion of participants with MWT SOL <8 minutes at both visits; the middle row shows the proportion of participants with MWT SOL ≥8 minutes at both visits; the bottom row shows the proportion of participants with mixed classification (above threshold at one visit, below threshold at the other). (B) Same as A, but for a cutoff of 20 minutes. (C) MWT SOL in minutes in each participant and test; color indicates shorter MWT SOL. (D) Values indicate change in MWT SOL (relative to baseline, in minutes) with color indicating larger absolute differences. Each row corresponds to a participant.
